# Deoxyhalogenation with Cu(III) Halide Complexes

**DOI:** 10.1021/acs.inorgchem.6c03041

**Published:** 2026-09-14

**Authors:** Joshua A. Queener, Ashleigh N. Sherman, Abigail M. Houser, Shiyu Zhang

**Affiliations:** Department of Chemistry and Biochemistry, 2647 The Ohio State University, Columbus, Ohio 43210, United States

## Abstract

While high-valent metal–halide complexes are widely studied as oxidants that promote C–H activation, their Lewis acidic reactivity has received little attention. Herein, we report a new mode of Cu­(III) reactivity in which Lewis acidic Cu­(III) complexes activate C­(sp^3^)–OH bond in alcohols and convert them to the corresponding alkyl fluorides, chlorides, and bromides. A series of formal Cu­(III) halide complexes (X = F, Cl, Br) supported by the tris­(2-pyridylthio)­methanide (TPTM, **L**) ligand were synthesized. These trigonal-bipyramidal [**L**Cu^III^–X]^+^ complexes feature an anionic carbon donor *trans* to the halide, resulting in weakened Cu–X bonds. Unlike conventional Cu­(III) complexes that typically promote hydrogen atom abstraction, [**L**Cu^III^–X]^+^ instead activates the C­(sp^3^)–OH bond for deoxyhalogenation. Mechanistic studies suggest that the reaction proceeds through initial activation of the C­(sp^3^)–OH bond by the Lewis acidic Cu^III^ center to generate a carbocation intermediate, which is subsequently trapped by halides to form the C­(sp^3^)–X bond. While Lewis acid-promoted deoxychlorination and deoxybromination reactions have been reported previously, this study represents the first example of a metal complex capable of mediating a general deoxyhalogenation series including F, Cl, and Br transfer.

## Graphical abstract

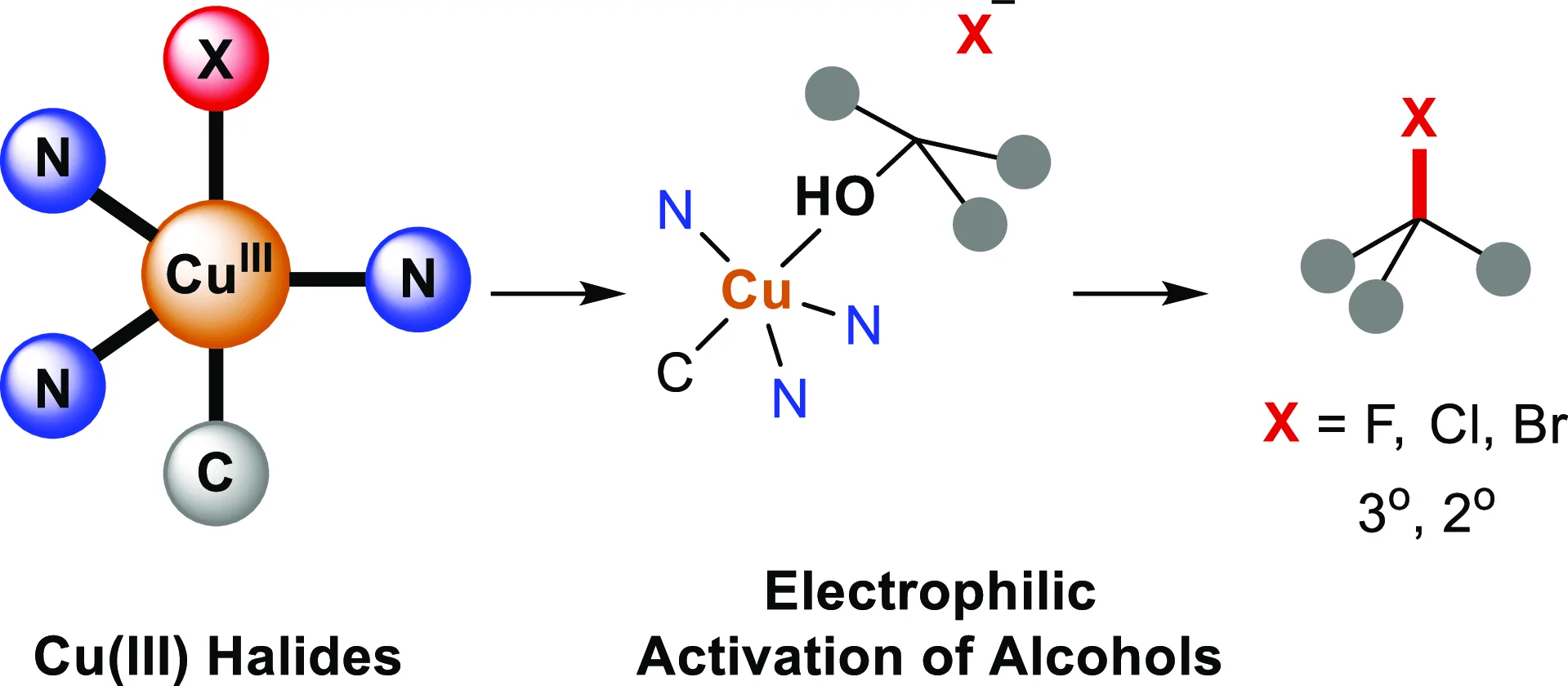

## Introduction

The development of metal complexes in unusual oxidation states has opened new avenues for challenging bond activation and functionalization.^1–4^ For example, a wide range of high-valent metal–fluoride complexes, such as Pd­(IV),^5^ Ni­(IV),^6^ Ni­(III),^7^ and Cu­(III)^8–11^ (Scheme 1A), have been developed to construct C–F bonds. Studies by Ritter, Sanford, and Hartwig demonstrated C­(sp^2^)–F bond formation through reductive elimination at Pd­(IV), Ni­(IV), and Cu­(III) fluoride centers.^5,6,8^ Additionally, McDonald, Cho, Groves, and our laboratory demonstrated that formal Cu­(III),^8–11^ Ni­(III),^7^ Fe­(III),^12^ Co­(IV),^13,14^ and Mn­(IV)^15^ fluoride complexes can convert C­(sp^3^)–H to C­(sp^3^)–F bonds via sequential hydrogen atom transfer (HAT) and halogen radical rebound (or radical capture, RC, Scheme 1B).

**Scheme 1:**
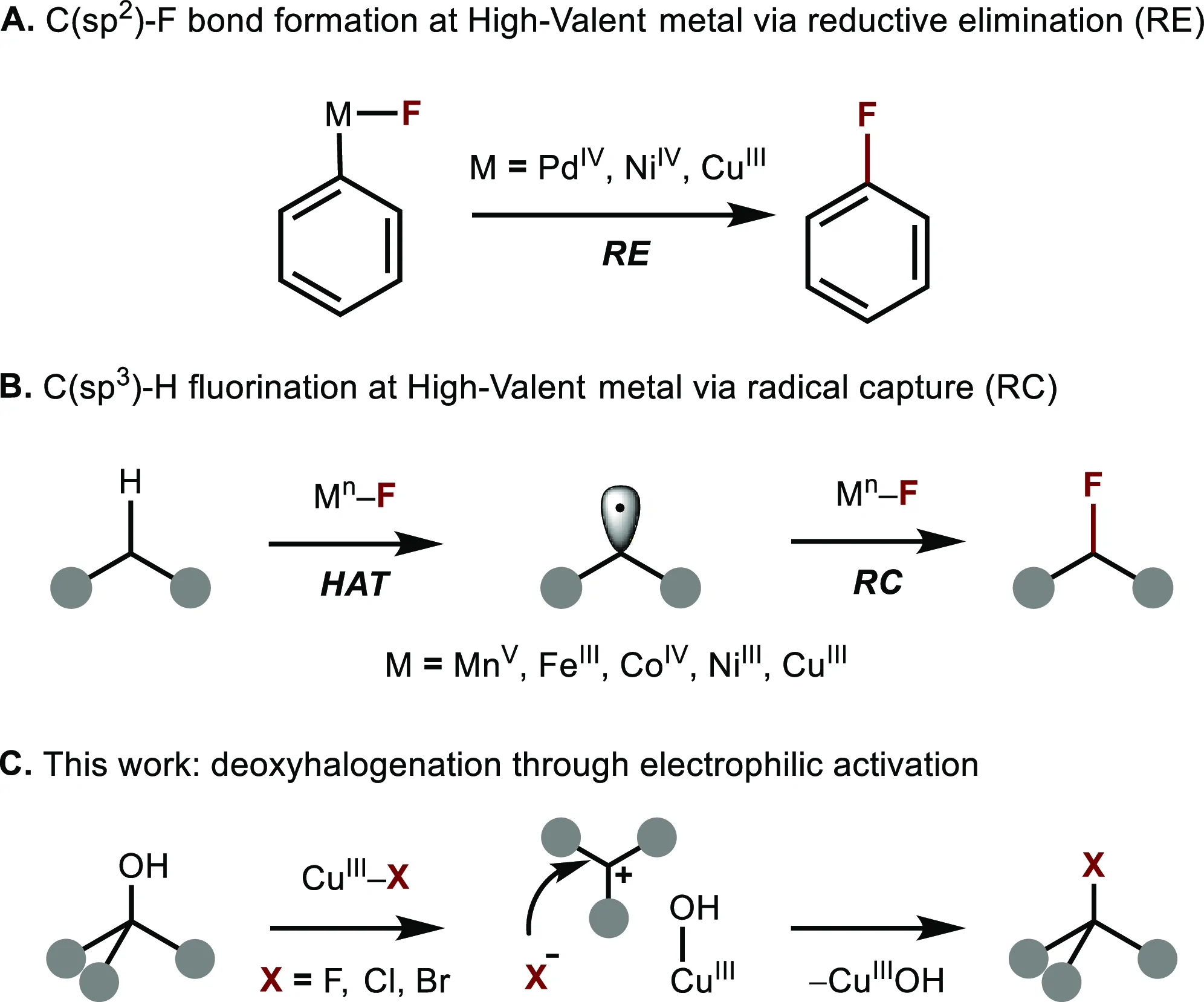
C–F Bond Formation via Metal–Fluoride Complexes

While the oxidative reactivities of high-valent metal-halide complexes have been explored extensively,^16–26^ their Lewis acidic properties remain underutilized in organometallic chemistry. Recent studies have shown that a wide range of +III metal centers, such as B­(III),^27–31^ Fe­(III),^32–35^ Bi­(III),^36^ In­(III),^37,38^ and Ga­(III),^39,40^ can promote C­(sp^3^)–OH bond activation. However, these reactions are largely limited to deoxychlorination and deoxybromination, and deoxyfluorination has not been achieved with high-valent metal–fluoride complexes. Given the well-known propensity of Cu­(III) at promoting C­(sp^3^)–F bond formation,^8–11^ we reasoned that a formal Cu­(III) complex, with its high positive charge and expected strong Lewis acidity, should be capable of activating C­(sp^3^)–OH bonds toward general deoxyhalogenation, including deoxyfluorination.^41^

Herein, we illustrate this concept using a series of electrophilic Cu­(III)–halide (X = F, Cl, Br) complexes (Scheme 1C). These formal Cu­(III) complexes are supported by the tris­(2-pyridylthio)­methanide (TPTM, L) ligand,^42,43^ featuring an anionic carbon donor *trans* to the halides. Unlike typical formal Cu­(III)–X species, which act as oxidants to promote C­(sp^3^)–H halogenation, the formal Cu­(III) center in LCu^III^–X functions as a Lewis acid, activating the C­(sp^3^)–OH bonds of alcohols and enabling halide capture by the resulting carbocation to afford alkyl halides (X = F, Cl, Br), completing a deoxyhalogenation sequence.

## Results and Discussion

### Synthesis and Characterization of Trigonal Bipyramidal LCu^II^–X Complexes

The tetradentate ligand **L** was synthesized using 2-mercaptopyridine and bromoform in high yield (Figure 1A). While the synthesis of **L**Cu^II^–X (X = F, Cl, Br, I) has been previously reported, the reported metalation methods require multiple days of reaction time and manual separation of the crystal mixtures, which are impractical for large-scale synthesis.^42,43^ We developed a new gram-scale synthesis of **L**Cu^II^–F by deprotonating LH with (trimethylsilyl)­methyllithium (TMSLi), followed by treatment with CuCl_2_, along with four equivalents of CsF. The analogous **L**Cu^II^–Cl and **L**Cu^II^–Br were synthesized by metalating the lithiated ligand **L**Li with CuCl_2_ and CuBr_2_. The identities of all **L**Cu^II^–X complexes were confirmed with single-crystal X-ray diffraction (XRD), electron paramagnetic resonance (EPR), and UV–vis analysis.

**Figure 1:**
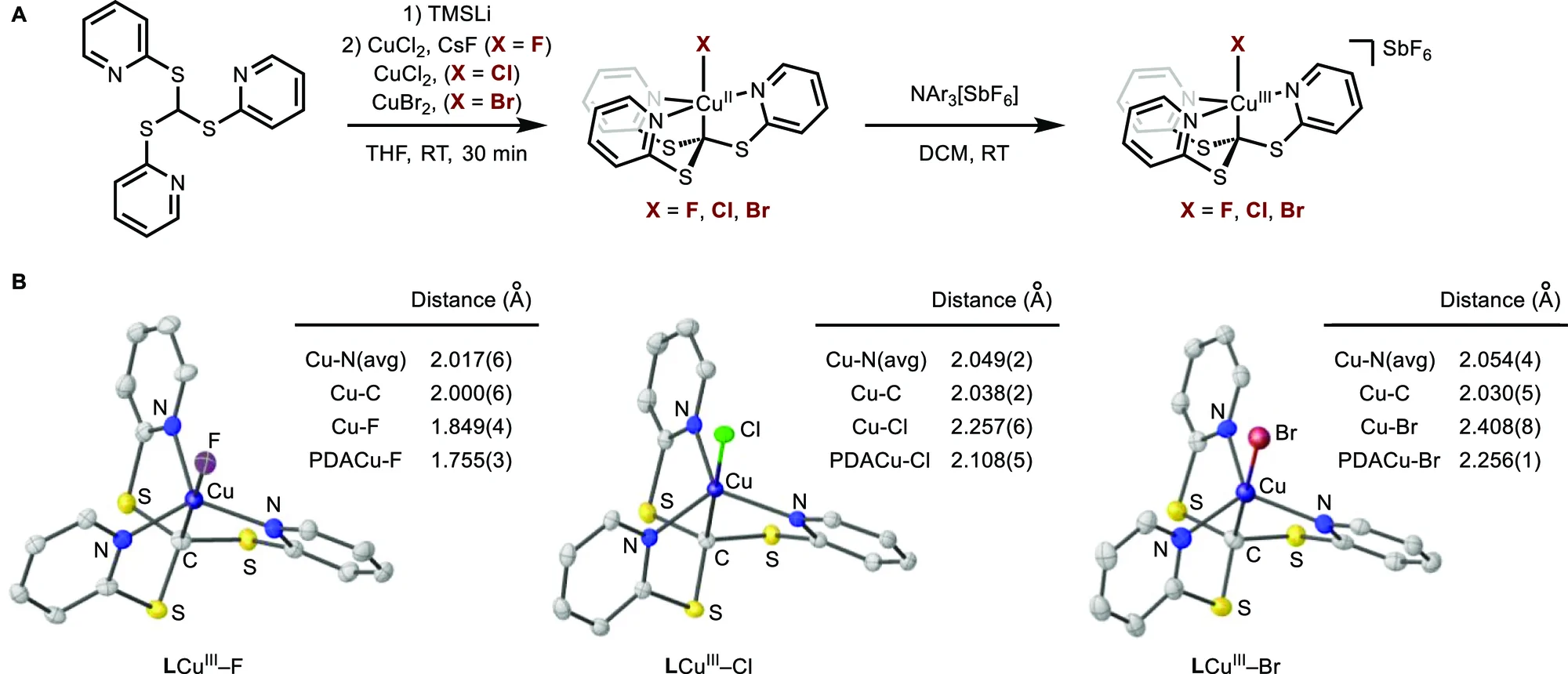
(A) Synthesis of formal copper­(III) halide complexes by chemical oxidation of the copper­(II) halide precursors with [NAr_3_]­SbF_6_ (Ar = 4-bromophenyl). (B) Solid-state structures of [**L**Cu^III^–F]^+^, [**L**Cu^III^–Cl]^+^, and [**L**Cu^III^–Br]^+^ with thermal ellipsoids shown at 50% level of probability. Cocrystallized solvents and minor components of disorder are omitted for clarity. Selected bond lengths (Å) in comparison to PDACu^III^–X complexes are previously reported by our laboratory.^9^

Cyclic voltammetry (CV) of **L**Cu^II^–X complexes shows reversible oxidations to the formal Cu­(III) state (*E*_1/2_; F: 31 mV, Cl: 40 mV, Br: 50 mV vs ferrocene/ferrocenium (Fc/Fc^+^)) and irreversible reductions to **L**Cu^I^–X (peak potentials; F: −1.66 V, Cl: −1.21 V, Br: –1.06 V vs Fc/Fc^+^). The redox potentials of Cu­(II)/Cu­(III) for all **L**Cu^II^–X complexes are essentially identical, suggesting minimal halide contribution to the singly occupied molecular orbital (SOMO) and large contribution to the SOMO from the ligand. The redox behaviors of **L**Cu^II^–X are in contrast to those of square-planar PDACu^II^–X complexes possessing only Cu­(II)/Cu­(III) couples (PDA = pyridinediamide, F: 465 mV, Cl: 525 mV, Br: 525 mV vs Ag/AgNO_3_), which can vary with halide identity.^9^ This difference in the electronic structures of trigonal-bipyramidal and square-planar Cu­(III)–X complexes will be further discussed in the context of their divergent HAT reactivities.

### Synthesis and Characterization of Trigonal Bipyramidal LCu^III^–X Complexes

Chemical oxidation of **L**Cu^II^–X to the formal [**L**Cu^III^–X]^+^ species was achieved using a triarylammonium radical cation (Magic blue), affording [**L**Cu^III^–X]­[SbF_6_] in up to a 96% yield. Upon oxidation of [**L**Cu^III^–F]­[SbF_6_], the color of the solution changes from green to dark red with strong UV–vis absorption bands from 400 to 600 nm (ε = 5770 M^–1^ cm^–1^, Figure S4). The oxidation of **L**Cu^II^–X causes a contraction of the Cu–X and the Cu–N bonds while leaving the Cu–C bond lengths relatively unchanged (see Supporting Information).

The [**L**Cu^III^–F]^+^ complex represents a rare example of structurally characterized formal Cu­(III)–F species.^9,44–46^ Notably, the Cu­(III)–F bond in [**L**Cu^III^–F]^+^ is longer (1.849(2) Å) than those in the square-planar PDACu^III^–F complex (1.755(3) Å) and (CF_3_)_3_Cu^III^–F (1.792(1) Å). This elongation likely reflects the *trans* influence of the anionic carbon ligand, which weakens the Cu–F bond. The single-crystal XRD structures of [**L**Cu^III^–Cl]­[SbF_6_] and [**L**Cu^III^–Br]­[SbF_6_] also reveal a similar *trans* influence of the anionic C donor, with elongated Cu–Cl (2.257(6) Å) and Cu–Br (2.408(8) Å) bonds (Figure 1B).

### Electronic Structure of [LCu^III^–F]^+^, Hydrogen Atom Transfer, and Radical Capture Reactivity

The electronic structure of the [**L**Cu^III^–F]^+^ was analyzed via density functional theory (DFT) calculations at the B3LYP/TZVP level of theory. The LUMO of [**L**Cu^III^–F]^+^ is primarily located on the ligand L (62.0%) with minor contributions from Cu­(d_z2_) (32.2%) and F­(p) orbitals (5.8%), consistent with the description of an inverted ligand field.^47–49^ Compared to the previously reported PDACu^III^–F species, the contribution of the Cu center to LUMO is reduced from 36.6% for PDACu^III^–F to 32.2% for [**L**Cu^III^–F]^+^ (Figure 2A).

**Figure 2:**
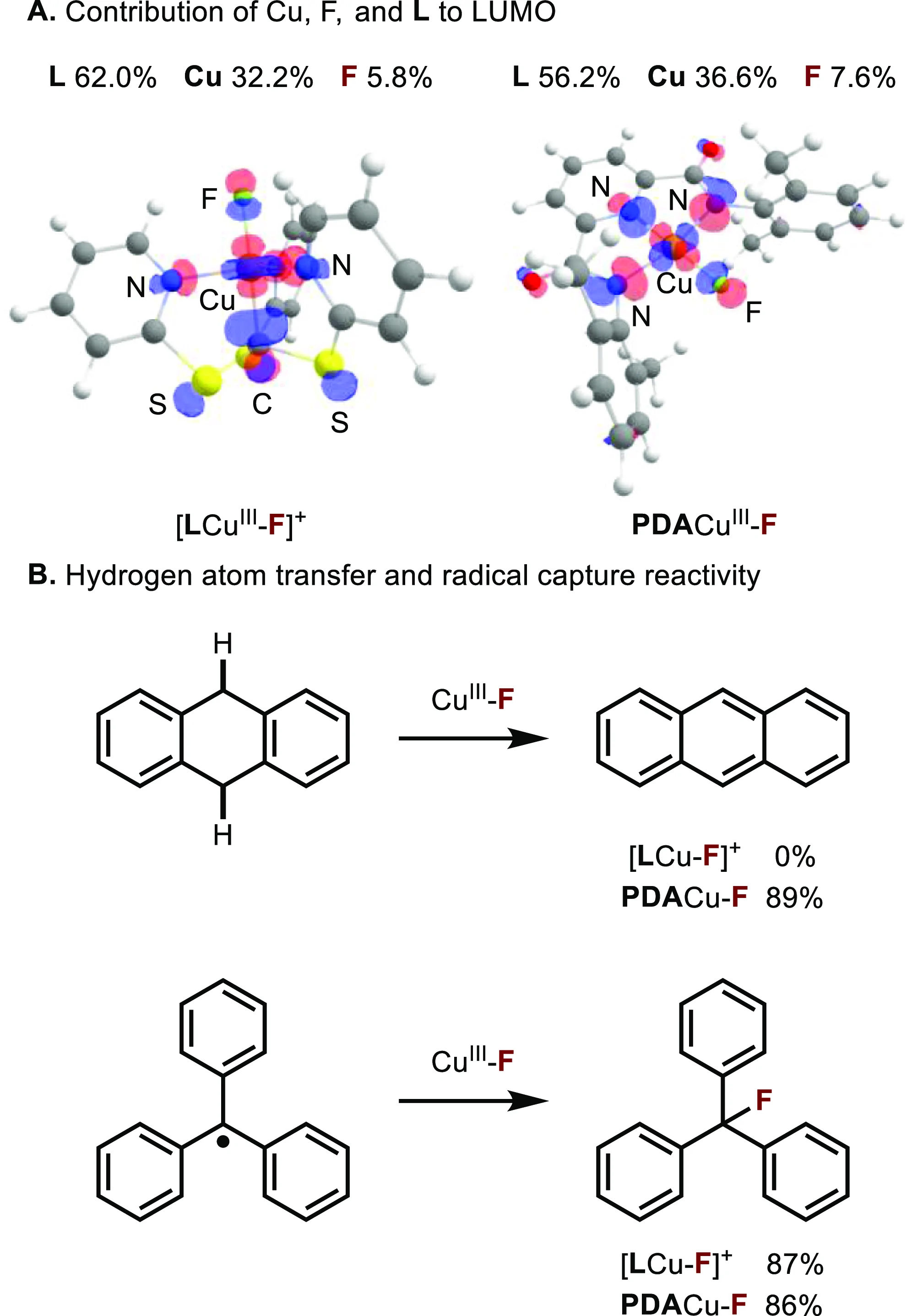
(A) SOMO plots of [**L**Cu^III^–F]^+^ (left) vs PDACu^III^–F (right) at the B3LYP/TZVP level of theory. (B) HAT and radical capture reactivity of [**L**Cu^III^–F]^+^ vs PDACu^III^–F.

Whereas the PDACu^III^–F complex abstracts hydrogen atom (HAT) from dihydroanthracene efficiently to afford anthracene in 89% yield, [**L**Cu^III^–F]^+^ exhibits no detectable HAT reactivity. Notably, the low halide-hole character in [**L**Cu^III^–F]^+^ does not preclude radical capture. Similar to PDACu^III^–F, which traps the trityl radical released from Gomberg’s dimer to form Ph_3_C–F in an 86% yield, [**L**Cu^III^–F]^+^ also captures a trityl radical to deliver Ph_3_C–F in an 87% yield (Figure 2B).

### Reactivity of [**L**Cu^III^–X]^+^ toward Deoxyhalogenation

Although [**L**Cu^III^–X]^+^ complexes lack HAT reactivity seen in the PDACu^III^ system,^10,50–55^ the cationic nature of [**L**Cu^III^–X]^+^ suggests that they might function as a Lewis acid for C­(sp^3^)–OH activation. Electrophilic metal centers are known to activate C­(sp^3^)–OH bonds toward nucleophilic substitution.^33–35,56–59^ Such direct substitution of the C­(sp^3^)–OH group with halides is a valuable reaction given the ubiquity of alcohols and the fact that water is the only stoichiometric byproduct.

To evaluate the ability of the [**L**Cu^III^–X]^+^ complexes to activate alcohols, three formal Cu­(III) halide complexes were treated with 1-adamantanol (**1**, Table 1). Excitingly, each reaction afforded the corresponding deoxyhalogenation product: 1-fluoroadamantane (**1a**, 15%), 1-chloroadamantane (**1b**, 31%), and 1-bromoadamantane (**1c**, 57%).

**Table 1:**
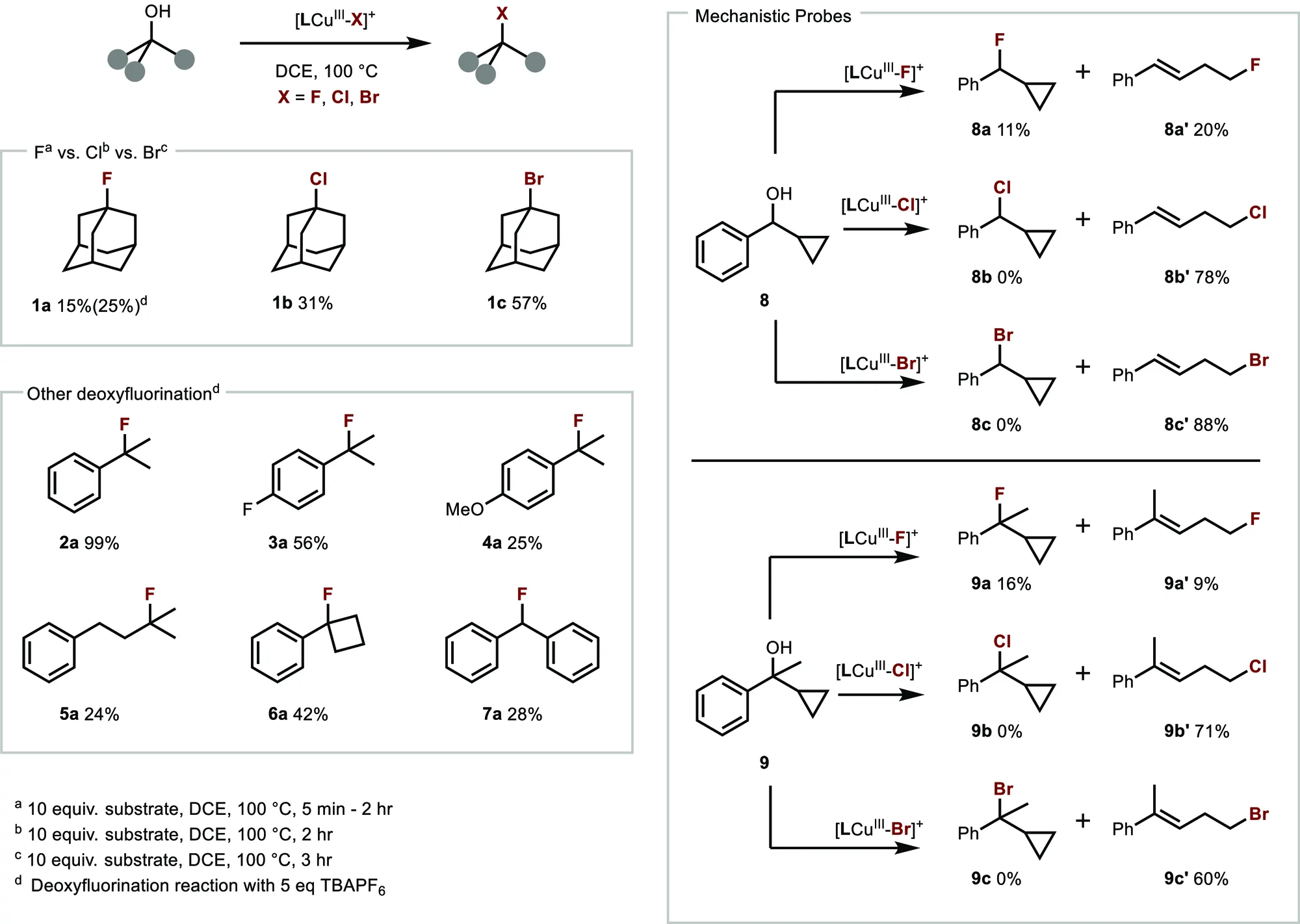
Deoxyhalogenation Reactivity of LCu^III^–X Complexes (X = F, Cl, Br)

The enhanced deoxyhalogenation efficiency observed in the order F < Cl < Br is likely a consequence of the decreasing Cu–X binding affinity across the halide series. As the halide becomes more weakly bound, dissociation of the halide ligand and subsequent coordination of the alcohol substrate become increasingly favorable, thereby facilitating activation of the C­(sp^3^)–OH bond. Treatment of PDACu^III^–F and **L**Cu^II^–X with alcohol 1 under similar conditions showed both complexes were unable to perform the deoxyfluorination reaction.

Next, we evaluated the Lewis acidity of the [**L**Cu^III^–X]^+^ series using a modified Gutmann–Beckett method.^60–62^ This approach involves ^31^P NMR analysis of mixtures of [**L**Cu^III^–X]^+^ with triphenylphosphine oxide (OPPh_3_). Coordination of the O atom of OPPh_3_ to the formal Cu­(III) center induces deshielding of the ^31^P nucleus, resulting in a downfield shift. The magnitude of the chemical shift change (Δδ) serves as a qualitative measure of Lewis acidity, with larger downfield shifts corresponding to stronger Lewis acids.

Using this method, the coordination-induced chemical shift changes (Δδ) for the [**L**Cu^III^–X]^+^ complexes were determined to be 0.0, 0.73, and 1.45 ppm for X = F, Cl, and Br, respectively (see Figures S14 and S15). These results indicate a modest increase in Lewis acidity across the halide series, following the trend [**L**Cu^III^–F]^+^ < [**L**Cu^III^–Cl]^+^ < [**L**Cu^III^–Br]^+^. However, the small Δδ values indicate that all three [**L**Cu^III^–X]^+^ complexes are relatively weak Lewis acids. Consequently, we consider them unlikely to be the species responsible for activation of the C­(sp^3^)–OH bond during deoxyhalogenation.

To evaluate the possibility of halide substitution as the mechanistic pathway, we measured the Lewis acidity of [**L**Cu^III^–MeCN]^2+^ under these conditions and observed a shift in ^31^P NMR resonance (Δδ) of 14.2 ppm. This value is comparable to the Δδ of 17.65 ppm induced by the strong Lewis acid tris­(pentafluorophenyl)­borane (BCF), indicating that [**L**Cu^III^–MeCN]^2+^ is a highly Lewis acidic species capable of activating the alcohol substrate through coordination.

The reaction of [**L**Cu^III^–F]^+^ with alcohols is expected to form [**L**Cu^III^–OH]^+^, which could reintroduce the hydroxide through substitution of the fluoride. To bias this equilibrium toward C­(sp^3^)–F bond formation, we employed additional equivalents of PF_6_^–^ as a hydroxide trap and exogenous fluoride source, drawing inspiration from Marek’s work,^56^ which demonstrated that BF_4_^–^ can serve as an effective fluoride donor. Accordingly, the addition of five equivalents of TBAPF_6_ to the deoxyfluorination reaction increased the yield of **2a** from 40% to 99%. A full optimization study, including the effects of temperature, solvent, and reaction time, is provided in the Supporting Information.

Given the importance of fluorinated organic compounds in pharmaceutical applications, we briefly explored the substrate scope of this deoxyfluorination protocol (**2**–**7**, Table 1). Several secondary and tertiary alcohols were successfully converted to the corresponding alkyl fluorides in 24–99% yields (2a–7a). Despite the success of these conditions, we observe the formation of the corresponding alkene via elimination, a common decomposition pathway for tertiary halides (see Supporting Information).

To further elucidate the reaction mechanism, we investigated the deoxyhalogenation of substrates bearing cyclopropyl motifs. Treatment of cyclopropyl carbinol **8** with [**L**Cu^III^–F]^+^ under standard conditions afforded a mixture of the direct deoxyfluorination product **8a** and the ring-opening deoxyfluorination product **8a′** in a 1:2 ratio (Table 1, right).

The formation of the ring-opened product **8a′** could, in principle, arise from either a carbon-centered radical or a carbocation intermediate.^63^ However, a radical pathway would require access to a formal Cu­(IV) species, which is highly unlikely. Instead, the observed reactivity is most consistent with a carbocation intermediate that undergoes rapid ring-opening rearrangement.^56^

Additional support for the carbocation intermediate was obtained from the deoxyfluorination of the analogous tertiary alcohol **9**. In this case, the ring-opening product 9a′ was formed in a lower yield than that of the unrearranged product **9a** (Table 1). This reversed product distribution is consistent with increased stability of tertiary carbocations, which reduces the driving force for rearrangement.

To gain insights into the fate of the copper center, we performed spectroscopic analysis of the reaction mixture upon completion of the deoxyhalogenation. ^1^H NMR spectroscopy showed no detectable Cu­(III) species, suggesting that the copper is perhaps converted to a paramagnetic Cu­(II) state. Although deoxyhalogenation with [**L**Cu^III^–X]^+^ is expected to generate [**L**Cu^III^–OH]^+^, this intermediate appears to be unstable and rapidly decomposes to Cu­(II) (see Supporting Information).

With this information in hand, we performed X-band EPR analysis of the post-deoxyhalogenation mixture. Upon addition of MeCN, [**L**Cu^II^(MeCN)]^+^ was detected in a 34% yield. This observation is consistent with the initial formation of an unobserved [**L**Cu^III^–OH]^+^ intermediate that decomposes under the reaction conditions to afford **L**Cu^II^–OH_2_ species. Attempts to synthesize and isolate [**L**Cu^III^–OH]^+^ were unsuccessful (see Supporting Information).

Taken together, these mechanistic studies support a deoxyhalogenation pathway that begins with dissociation of the halide ligand, followed by coordination of the alcohol substrate to the highly Lewis acidic Cu­(III) center. This coordination activates the C­(sp^3^)–OH bond, enabling its conversion to a carbocation-like intermediate, which is subsequently trapped by the halide to afford the deoxyhalogenation product. The resulting copper species is proposed to be [**L**Cu^III^–OH]^+^; however, based on our experimental observations, this species is highly unstable and undergoes rapid decomposition in solution (Figure 3).

**Figure 3:**
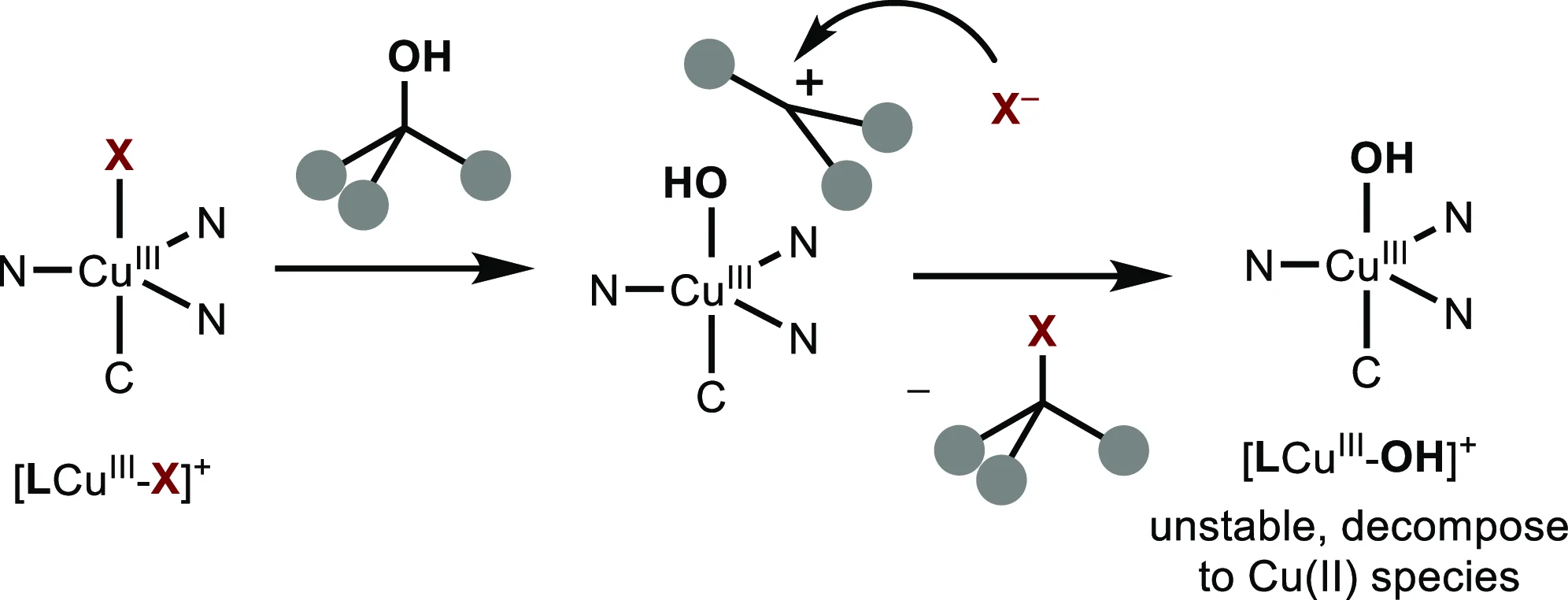
Proposed mechanism for deoxyhalogenation by the [LCu^III^–X]^+^ complex.

## Conclusion

In summary, we have synthesized and fully characterized a series of cationic copper­(III) halide complexes, [**L**Cu^III^–X]^+^ (X = F, Cl, Br), and demonstrated that these complexes can activate C­(sp^3^)–OH bonds toward halide substitution in a 25–99% yield. Mechanistic studies indicate that the reaction proceeds through initial activation of the C­(sp^3^)–OH bond by the Lewis acidic Cu^III^ center to generate a carbocation intermediate, which is subsequently trapped by the Cu^III^–halide complex to form the C­(sp^3^)–X bond.

While Lewis acid-promoted deoxychlorination and deoxybromination reactions have been reported previously, this study represents the first example of a metal complex capable of mediating a general deoxyhalogenation series including F, Cl, and Br transfer. This reactivity uncovers a previously unrecognized reaction manifold for high-valent copper complexes beyond oxidative C–H functionalization and establishes a new conceptual framework for halogen-transfer chemistry. Our laboratory is currently investigating the mechanistic relevance of this reactivity in the context of newly discovered biological copper-mediated halogenation reactions.^18^

## Experimental Section

### General

All syntheses and experiments were performed under a nitrogen atmosphere in an MBraun glovebox or using standard Schlenk techniques unless otherwise noted. Dichloromethane, tetrahydrofuran, acetonitrile, pentane, and diethyl ether were dried and degassed under nitrogen using a Pure Process Technologies (PPT, Nashua, NH) solvent purification system and stored over 4 Å molecular sieves. Dichloromethane-*d*_2_ and chloroform-*d*_1_ (Cambridge Isotope Laboratories, Inc.) were dried over 4 Å molecular sieves prior to use. All glassware was dried at 120 °C prior to use. NAr_3_[SbF_6_] and [**L**CuOH·TEAH]­[PF_6_] were synthesized as previously described.^64,65^ All other reagents were obtained from reputable suppliers and used without further purification. NMR spectra were recorded on a Bruker Avance NEO 400 MHz instrument, a Bruker Avance III HD 600 MHz instrument, or a Bruker Ascend 700 MHz instrument and referenced to residual solvent peaks (or α,α,α-trifluorotoluene or fluorobenzene for ^19^F NMR). NMR multiplicities are reported as follows: singlet (s), doublet (d), triplet (t), quartet (q), multiplet (m), and broad signal (br). For quantitative NMR experiments, a multi-pulse sequence was used with increased relaxation delays. For ^19^F NMR, a relaxation delay of 5 s was used; and for ^1^H NMR, a relaxation delay of 3 s was used. UV–vis spectra were collected on an Agilent Cary 60 spectrophotometer outfitted with an UnisokuUnispeks cryostat (–100 °C to +100 °C). High-resolution mass spectra were recorded on a Bruker MicrOTOF (ESI). ESI-MS samples were run in chloroform solvent at concentrations <1 μM. GC analysis was performed on an Agilent 7890B GC equipped with an HP-5 column and FID detector. Elemental analysis was performed by Midwest Micro Lab (Indianapolis, IN, http://midwestlab.com/). Low-temperature EPR spectra were recorded on a Magnettech ESR5000 benchtop EPR (continuous-wave, X-band) spectrometer equipped with a LN2 finger dewar at 77 K. Cyclic voltammograms were recorded under a nitrogen atmosphere with a Biologic SP-150 potentiostat using a three-electrode system comprised of a glassy carbon button working electrode, a platinum wire counter electrode, and a Ag/AgNO_3_ pseudo-reference electrode. All cyclic voltammograms were referenced internally to Fc/Fc+. Compounds were dissolved in an electrolyte solution consisting of 0.100 M electrochemical grade tetrabutylammonium perchlorate. ATR-IR spectra were measured using a Bruker Alpha II spectrometer with a diamond ATR accessory.

### Computational Details

DFT calculations were performed in ORCA,^66,67^ and geometry optimizations were performed at the B3LYP/6-31G­(d) (SDD for Cu, Br) level of theory with D3BJ dispersion correction. A frequency calculation was then performed on the optimized structures at the same level of theory, which confirmed the absence of imaginary frequencies. A single-point energy calculation on the optimized structure was then performed at the B3LYP/def2-TZVP (SDD for Cu, Br) level of theory, with CPCM solvent modeling (dichloromethane) and D3BJ dispersion correction.

## Supplementary Material



## Data Availability

Crystallographic data for [LCu^III^–F]­[SbF6] (2533594); (CIF) Crystallographic data for [LCu^III^–Cl]­[SbF_6_] (2533595); (CIF) Crystallographic data for [LCu^III^–Br]­[SbF_6_] (2533603).

## Notes

Experimental details, including characterization data, spectra, kinetic data, computational procedures, and results (PDF)
