# Supplementary material for: Deoxyhalogenation with Cu(III) Halide Complexes
Source: Inorg Chem. 2026 Sep 14;65(38):22453–9. doi: 10.1021/acs.inorgchem.6c03041 (PMC13625957; doi:10.1021/acs.inorgchem.6c03041)
Supplement: Supplementary file 1 [file ic6c03041_si_001.pdf]

Supporting Information

Deoxyhalogenation with Cu(III) Halide Complexes

Joshua A. Queener, Ashleigh N. Sherman, Abigail M. Houser, Shiyu Zhang*

Department of Chemistry and Biochemistry, The Ohio State University, 151 W Woodruff Avenue, Columbus, OH 43210, United States

*zhang.8941@osu.edu

Contents

General experimental details....................2
Synthesis of compounds....................3
Electrochemical measurements....................8
UV-vis spectroscopic studies....................10
Estimation of Lewis acidity using a modified Guttman-Beckett method....................14
Procedure for trityl radical capture with $[LCu^{III}X]^+$ complexes....................18
Procedures for deoxyhalogentation of alcohols....................20
EPR spin quantization of $[LCu^{II}MeCN]^+$ after deoxyfluorination....................32
Computational details....................35
Other NMR spectra....................38
X-Ray crystallographic details....................44
References....................86

General experimental details

All syntheses and experiments were performed under a nitrogen atmosphere in an MBraun glovebox or using standard Schlenk techniques unless otherwise noted. Dichloromethane, tetrahydrofuran, acetonitrile, pentane, and diethyl ether were dried and degassed under nitrogen using a Pure Process Technologies (PPT, Nashua, NH) solvent purification system and stored over 4 Å molecular sieves. Dichloromethane-d_2 and chloroform-d_1 (Cambridge Isotope Laboratories, Inc.) were dried over 4 Å molecular sieves prior to use. All glassware was dried at 120 °C prior to use. $NAr_3[SbF_6]$, and [**L**CuOH•TEAH][PF_6] were synthesized as previously described.[1,2] All other reagents were obtained from reputable suppliers and used without further purification. NMR spectra were recorded on a Bruker Avance NEO 400 MHz instrument, a Bruker Avance III HD 600 MHz instrument, or a Bruker Ascend 700 MHz instrument and referenced to residual solvent peaks (or α,α,α-trifluorotoluene or fluorobenzene for ^{19}F NMR). NMR multiplicities are reported as follows: singlet (s), doublet (d), triplet (t), quartet (q), multiplet (m), broad signal (br). For quantitative NMR experiments, a multi-pulse sequence was used with increased relaxation delays. For ^{19}F NMR, a relaxation delay of 5 seconds was used; and for ^{1}H NMR, a relaxation delay of 3 seconds was used. UV-vis spectra were collected on an Agilent Cary 60 spectrophotometer outfitted with an UnisokuUnispeks cryostat (−100 °C to + 100 °C). High-resolution mass spectra were recorded on a Bruker MicrOTOF (ESI). ESI-MS samples were run in chloroform solvent at concentrations < 1 μM. GC analysis was performed on an Agilent 7890B GC equipped with an HP-5 column and FID detector. Elemental analysis was performed by Midwest Micro Lab (Indianapolis, IN, http://midwestlab.com/). Low Temperature EPR spectra were recorded on a Magnettech ESR5000 benchtop EPR (continuous-wave, X-band) spectrometer equipped with a LN2 finger dewar at 77K. Cyclic voltammograms were recorded under a nitrogen atmosphere with a Biologic SP-150 potentiostat using a three-electrode system comprised of a glassy carbon button working electrode, a platinum wire counter electrode, and a Ag/AgNO3 pseudo-reference electrode. All cyclic voltammograms were referenced internally to Fc/Fc+ . Compounds were dissolved in an electrolyte solution consisting of 0.100 M electrochemical grade tetrabutylammonium perchlorate. ATR-IR spectra were measured using a Bruker Alpha II spectrometer with a diamond ATR accessory.

Synthesis of compounds

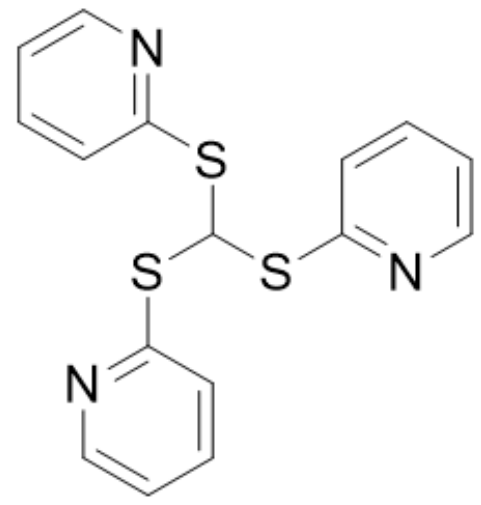

2,2'-(((pyridin-3-ylthio)methylene)bis(sulfanediyl))dipyridine (TPTM, LH) The synthetic protocol is based on a modified literature procedure.[3] To a dry round bottom flask equipped with a stir bar, K_2CO_3 (5.5282 g, 40 mmol, 4.0 equiv.) and 2-mercaptopyridine (3.4459 g, 31 mmol, 3.1 equiv.) were added. The solids were dissolved in acetone (300 mL). The reaction was then stirred for 2 hours, after which bromoform (0.874 mL, 10 mmol, 1.0 equiv.) was added. The reaction was heated to reflux for 48 hours. After cooling, the reaction was filtered. The solution was dried in vacuo, and the resulting residue was dissolved in ethanol (50 mL). The ethanol solution was concentrated until yellow crystals began to form. The saturated ethanol solution was then placed in the freezer overnight. The yellow crystals formed overnight were collected via vacuum filtration and then rinsed with water (50 mL, three times). The product was isolated as a yellow crystalline solid (2.3597 g, 68%). Spectroscopic characterization of the product is consistent with the literature report.[3]

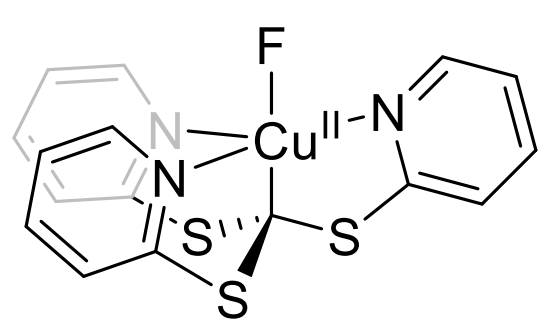

TPTMCu^{II}F To a 20 mL scintillation vial 2,2'-(((pyridin-3-ylthio)methylene)bis(sulfanediyl))dipyridine (**LH**, 2.00 g, 5.8 mmol, 1.0 equiv.) was added and dissolved in THF (4 mL). To a separate vial, TMSLi (546 mg, 5.8 mmol, 1.0 equiv.) was added and dissolved in THF (4 mL). Both vials were cooled to –35 °C, and the TMSLi solution was added to the **LH** solution. Immediately after the addition, the resulting solution was then added to a vial containing solid $Cu^{II}Cl_2$ (780 mg, 5.8 mmol, 1.0 equiv.). The reaction was stirred for 2 hours prior to being dried *in vacuo*. The resulting solids were dissolved in acetonitrile (10 mL) and then added to a vial containing CsF (3.50 g, 23.2 mmol, 4.0 equiv.) and stirred for 24 hours. The resulting solution was then filtered before being dried *in vacuo* and then being redissolved in DCM (5 mL). The DCM solution was then filtered and then dried *in vacuo*, yielding green crystals. The resulting crystals were rinsed with cold acetonitrile (5 mL, three times). The product was isolated as green crystals (1.20 g, 49%). The UV-vis and EPR spectroscopic characterization of the title compound match those reported by Kinoshita.[4]

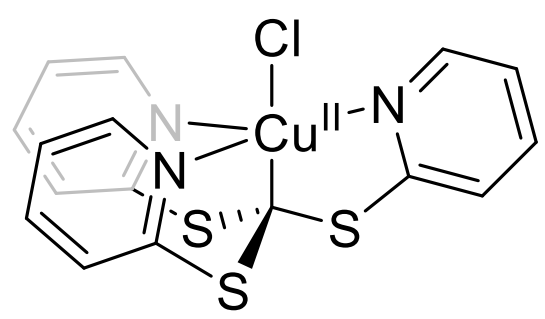

TPTMCu^{II}Cl To a 20 mL scintillation vial 2,2'-(((pyridin-3-ylthio)methylene)bis(sulfanediyl))dipyridine (**LH**, 1.00 g, 2.9 mmol, 1.00 equiv.) was added and dissolved in THF (6 mL). To a separate vial, TMSLi (274 mg, 2.9 mmol, 1.00 equiv.) was added and dissolved in THF (6 mL). Both vials were cooled to –35 °C, and the TMSLi solution was added to the **LH** solution. Immediately after the addition, the resulting solution was then added to a vial containing $Cu^{II}Cl_2$ (391 mg, 2.9 mmol, 1.00 equiv.). The reaction was stirred for 2 hours prior to being dried *in vacuo*. The resulting solids were dissolved in DCM (10 mL) and then filtered. The solution was then layered with pentane and stored at –35 °C The purple crystals formed overnight were collected and rinsed with diethyl ether (5 mL, three times), yielding a purple solid (768 mg, 60%). The UV-vis and EPR spectroscopic characterization of the title compound match those reported by Kinoshita.[4]

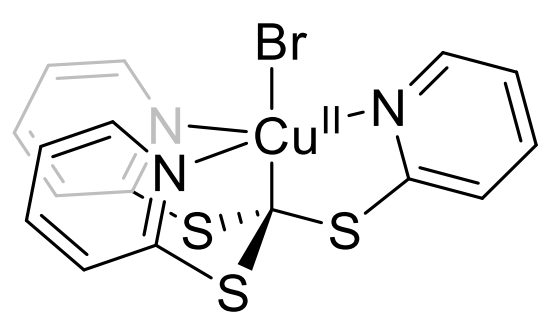

TPTMCu^{II}Br To a 20 mL scintillation vial 2,2'-(((pyridin-3-ylthio)methylene)bis(sulfanediyl))dipyridine (**LH**, 1.00 g, 2.9 mmol, 1.00 equiv.) was added and dissolved in THF (6 mL). To a separate vial TMSLi (274 mg, 2.9 mmol, 1.00 equiv.) was added and dissolved in THF (6 mL). Both vials were cooled to –35 °C prior to the TMSLi solution being added to the **LH** solution. After addition the resulting solution was then added to a vial containing $Cu^{II}Br_2$ (648 mg, 2.9 mmol, 1.00 equiv.). The reaction was stirred for 2 hours prior to being dried *in vacuo*. The resulting solids were dissolved in DCM (10 mL) and then filtered. The solution then layered with pentane and stored at –35 °C. After overnight purple crystals had formed, the solution was decanted, and the resulting crystals were rinsed with diethyl ether (5 mL, three times). The product was isolated as purple crystals (873 mg, 62%). The UV-vis and EPR spectroscopic characterization of the title compound match those reported by Kinoshita.[4]

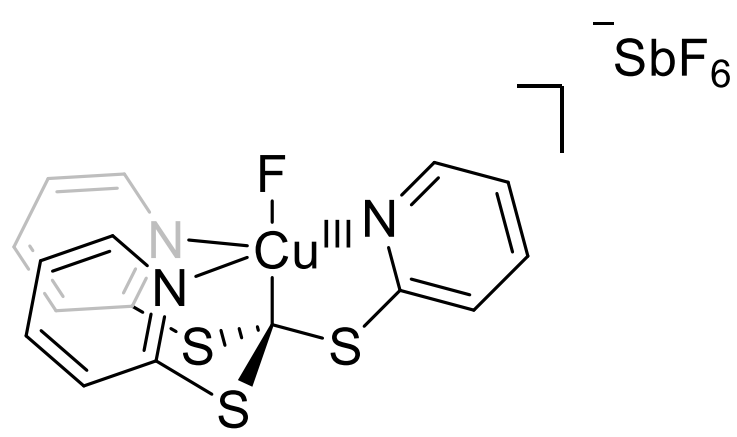

TPTMCu^{III}F[SbF$_6$] To a 100 mL round bottom flask, **L**$Cu^{II}F$ (1.20 g, 2.82 mmol, 1.00 equiv.) was added and dissolved in DCM (20 mL). $NAr_3[SbF_6]$ (2.00 g, 2.80 mmol, 0.999 equiv.) was then added to a 20 mL scintillation vial and dissolved in DCM (20 mL). The solution of $NAr_3[SbF_6]$ was then added to the solution of **L**$Cu^{II}F$. After the addition, the solution was filtered through a frit with celite into a 250 mL round bottom flask. The round bottom flask was then layered with pentane (100 mL). The dark red solids formed overnight were collected via vacuum filtration and rinsed with diethyl ether (100 mL, three times) to yield [**L**$Cu^{III}F$][SbF_6] as a red solid (1.82 g, 96%). Crystals suitable for SC-XRD were obtained from vapor diffusion of pentane into a DCM solution, resulting in needle-like crystals.

^{1}H NMR (600 MHz, CD_2Cl_2) δ 8.90 (br, 3H), 8.12 (t, J = 7.7 Hz, 3H), 7.94 (br, 3H), 7.75 (br, 3H).

^{13}C NMR (176 MHz, CD_2Cl_2) δ 162.43, 152.41, 142.39, 125.25, 123.74.

^{19}F NMR (377 MHz, CD_2Cl_2) δ −306.65.

Anal. Calcd. For $C_{16}H_{12}CuF_7N_3S_3Sb•CH_2Cl_2$: C, 27.28; H, 1.89; N, 5.64. Found: C, 27.28; H, 1.85; N, 5.51.

UV-vis: CH_2Cl_2 @ 20 °C: λ_{max} = 335 nm (ε = 1750 M^{-1} cm^{-1}), 426 nm (ε = 1660 M^{-1} cm^{-1}), 532 nm (ε = 5770 M^{-1} cm^{-1})

ATR-IR: (neat) cm^{-1} = 3085w, 3050w, 2993w, 1583w, 1559w, 1449w, 1427w, 1282w, 1265w, 1250w, 1158w, 1133w, 1089w, 1045w, 1016w, 855w, 839w, 783w, 767m, 729w, 704w, 650m, 500m, 478w, 461w, 417w.

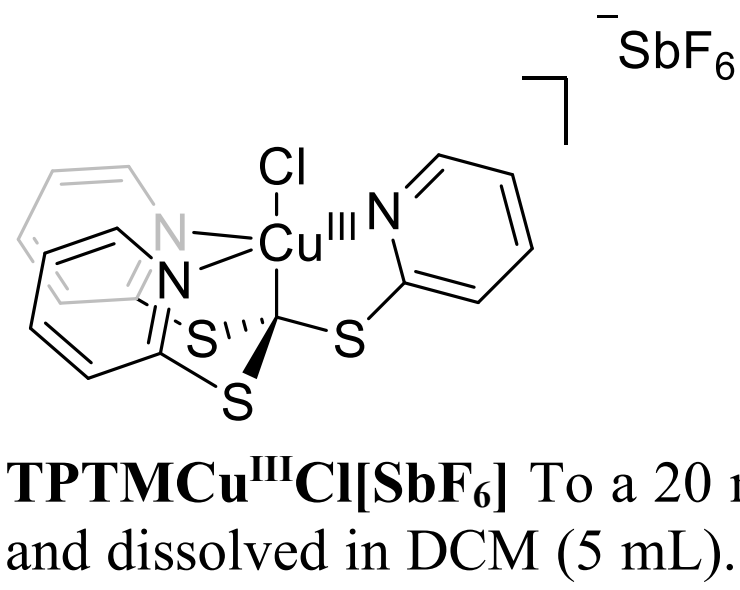

TPTMCu^{III}Cl[SbF$_6$] To a 20 mL scintillation vial **L**Cu^{II}Cl (500 mg, 1.13 mmol, 1.00 equiv.) was added and dissolved in DCM (5 mL). NAr$_3$[SbF$_6$] (803 mg, 1.12 mmol, 0.99 equiv.) was then added to a 20 mL scintillation vial and dissolved in DCM (10 mL). The solution of NAr$_3$[SbF$_6$] was then added to the solution of **L**Cu^{II}Cl. After the addition, the solution was filtered and then concentrated *in vacuo* to ca. 10 mL. Then the solution was layered with pentane. After overnight deep purple solids had crashed out of solution. The solids were collected via vacuum filtration and rinsed with diethyl ether (100 mL, three times) to yield [**L**Cu^{III}Cl][SbF$_6$] as a purple solid (704 mg, 92%). Single crystals suitable for SC-XRD were obtained from pentane vapor diffusion into DCM resulting in needle-like crystals.

^{1}H NMR (400 MHz, CD_2Cl_2) δ 9.32 (ddd, J = 5.6, 1.7, 0.8 Hz, 3H), 8.11 (td, J = 7.8, 1.7 Hz, 3H), 7.93 (dt, J = 8.0, 1.0 Hz, 3H), 7.76 (ddd, J = 7.6, 5.6, 1.2 Hz, 3H).

^{13}C NMR (176 MHz, CD_2Cl_2) δ 162.93, 153.42, 142.20, 124.43.

Anal. Calcd. For $C_{16}H_{12}ClCuF_6N_3S_3Sb$: C, 28.38; H, 1.79; N, 6.20. Found: C, 28.41; H, 1.83; N, 6.16.

UV-vis: CH_2Cl_2 @ 20 °C: λ_{max} = 351 nm (ε = 3470 M^{-1} cm^{-1}), 469 nm (ε = 6310 M^{-1} cm^{-1}), 552 nm (ε = 7320 M^{-1} cm^{-1})

ATR-IR: (neat) cm^{-1} = 3084w, 2970w, 1583w, 1559w, 1449w, 1428w, 1285w, 1158w, 1132w, 1116w, 1090w, 1013w, 856w, 766m, 726w, 653m, 570w, 496w, 462w, 413w.

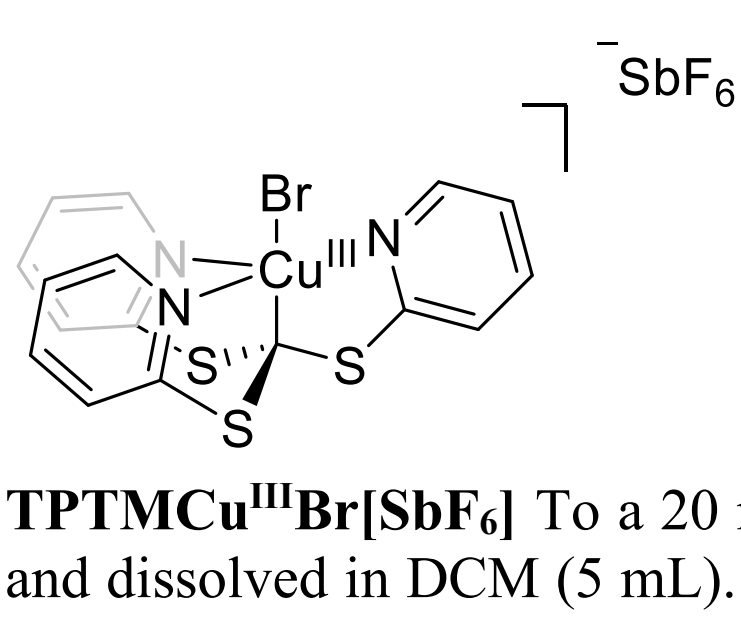

TPTMCu^{III}Br[SbF$_6$] To a 20 mL scintillation vial, **L**Cu^{II}Br (500 mg, 1.03 mmol, 1.0 equiv.) was added and dissolved in DCM (5 mL). NAr$_3$[SbF$_6$] (732 mg, 1.02 mmol, 0.99 equiv.) was then added to a 20 mL scintillation vial and dissolved in DCM (10 mL). The solution of NAr$_3$[SbF$_6$] was then added to the solution of **L**Cu^{II}Br. After the addition the solution was filtered and then concentrated in vacuo to ca. 10 mL. Then the solution was layered with pentane. After overnight deep purple solids had crashed out of solution. The solids were collected via vacuum filtration and rinsed with diethyl ether (100 mL, three times) to yield

[**L**Cu^{III}Br][SbF$_6$] as a purple solid (698 mg, 94%). Crystals Suitable for SC-XRD were obtained from pentane vapor diffusion into DCM resulting in needle-like crystals.

^{1}H NMR (400 MHz, CD$_2$Cl$_2$) δ 9.58 (s, 3H), 8.11 (td, *J* = 7.7, 1.6 Hz, 3H), 7.97 (d, *J* = 7.9 Hz, 3H), 7.76 (dd, *J* = 7.5, 5.6 Hz, 3H).

^{13}C NMR (176 MHz, CD$_2$Cl$_2$) δ 162.64, 154.58, 142.23, 124.49.

Anal. Calcd. For $C_{16}H_{12}BrCuF_6N_3S_3Sb$: C, 26.63; H, 1.68; N, 5.82. Found: C, 26.55; H, 1.62; N, 5.67.

UV-vis: CH_2Cl_2 @ 20 °C: λ_{max} = 360 nm (ε = 3460 M^{-1} cm^{-1}), 493 nm (ε = 9860 M^{-1} cm^{-1}), 566 nm (ε = 7210 M^{-1} cm^{-1})

ATR-IR: (neat) cm^{-1} = 3095w, 2968w, 1582w, 1559w, 1449w, 1428w, 1286w, 1241w, 1159w, 1090w, 1050w, 1011w, 980w, 859w, 765m, 726w, 652m, 569w, 493w, 463w, 413w.

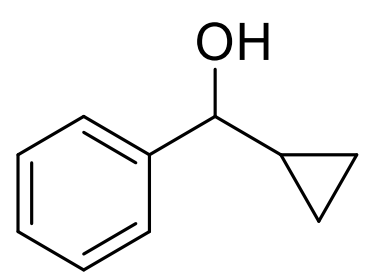

1-phenyl-1-cyclopropylmethanol The title compound was synthesized according to a published procedure; spectral data is consistent with reported literature values.[5]

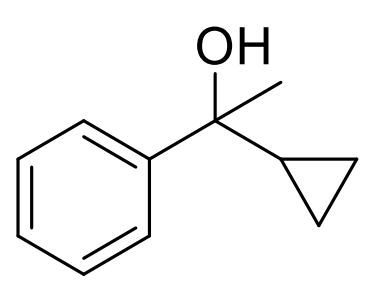

1-cyclopropyl-1-phenylethanol The title compound was synthesized according to a published procedure; spectral data is consistent with reported literature values.[6]

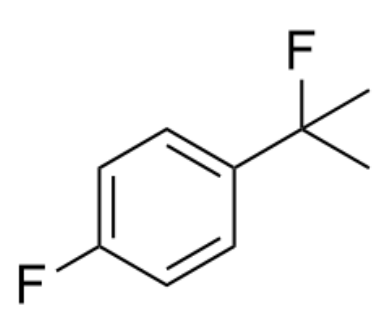

1-fluoro-4-(2-fluoropropan-2-yl)benzene To a 10 mL screw cap test tube equipped with a stir bar, 2-(4-fluorophenyl)propan-2-ol (100 mg, 0.649 mmol, 1.00 equiv.) and diethylaminosulfur trifluoride (0.257 mL, 1.94 mmol, 3.00 equiv.) were added. The solids were dissolved in dichloromethane (1 mL). The reaction was stirred for 2 hours. The final product was isolated via preparatory thin-layer chromatography (pure hexane) to yield the title compound as a colorless oil (5.2 mg, 5.1 %).

^{1}H NMR (400 MHz, CDCl$_3$) δ 7.37 – 7.33 (m, 1H), 7.03 (ddd, *J* = 9.1, 8.6, 0.8 Hz, 1H), 1.68 (d, *J* = 21.9 Hz, 3H).

^{13}C NMR (176 MHz, CDCl$_3$) δ 162.13 (d, *J* = 245.6 Hz), 141.80 (d, *J* = 22.8 Hz), 125.76 (t, *J* = 8.5 Hz), 115.17 (d, *J* = 21.4 Hz), 95.48 (d, *J* = 168.9 Hz), 29.47 (d, *J* = 26.0 Hz).

^{19}F NMR (376 MHz, CDCl$_3$) δ –115.78, –135.90 (hept, *J* = 21.8 Hz).

HRMS (ESI) (m/z): [M+H]$^+$ Calculated for $C_9H_{11}F_2$: 157.0823; found: 157.0835.

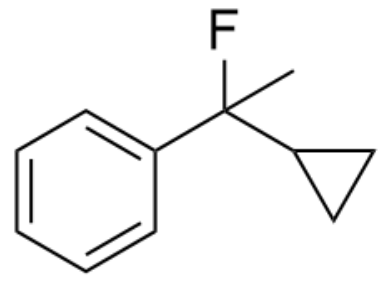

(1-cyclopropyl-1-fluoroethyl)benzene To a 10 mL screw cap test tube equipped with a stir bar, 1-cyclopropyl-1-phenylethan-1-ol (100 mg, 0.616 mmol, 1.00 equiv.) and diethylaminosulfur trifluoride (0.245 mL, 1.85 mmol, 3.00 equiv.) were added. The solids were dissolved in dichloromethane (1 mL). The reaction was stirred for 1 hour. Preparatory thin-layer chromatography was performed (95:5, hexane:ethyl acetate). Due to the instability of the title compound on silica, a mixture of product (confirmed with ^{19}F NMR and ESI-MS) and byproducts (elimination and ring-opening) were obtained as a clear oil.

^{19}F NMR (376 MHz, $CDCl_3$) δ –150.56 (hd, J = 21.8, 15.7 Hz).

HRMS (ESI) (m/z): $[M+H]^+$ Calculated for $C_{11}H_{14}F$: 165.1074; found: 165.1073.

Electrochemical measurements

Cyclic voltammograms were recorded under nitrogen atmosphere with a Biologic SP-150 potentiostat using a three-electrode system comprised of glassy carbon working electrode, platinum wire counter electrode, and nonaqueous silver nitrate reference electrode. All cyclic voltammograms were recorded at room temperature with a scan rate of 100 mV/s and internally referenced to ferrocene/ferrocenium. The CV solution was prepared by dissolving the copper complex (1.0 mM) in an electrolyte solution consisting of 0.1 M electrochemical grade tetrabutylammonium perchlorate in dichloromethane.

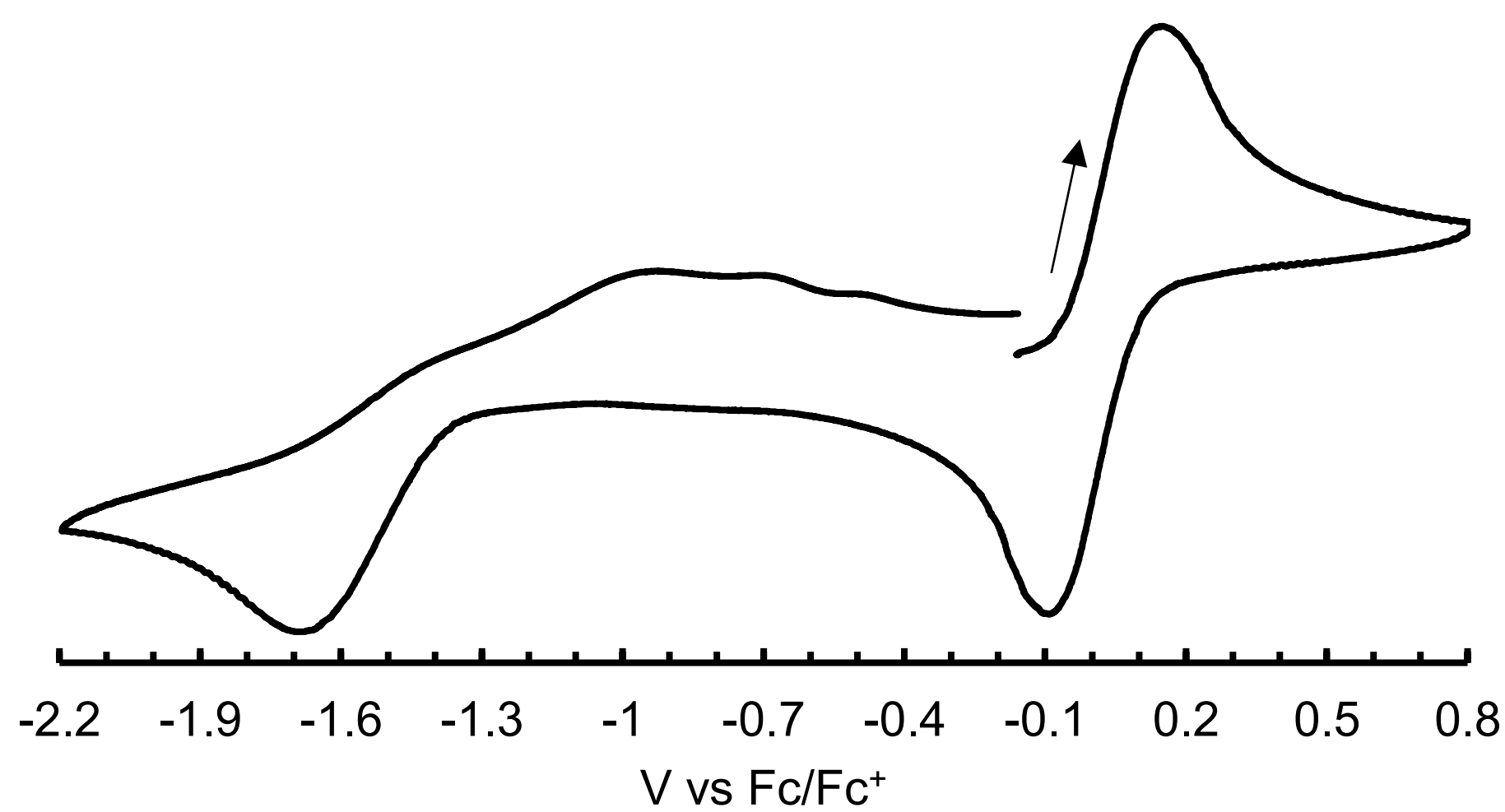

Figure S1. Cyclic voltammogram of **L**$Cu^{II}F$ in DCM at 100 mV/s. $Cu^{I/II}$ peak potential = –1.66 V vs Fc/Fc^{+}, $Cu^{II/III}$ $E_{1/2}$ = 31 mV vs Fc/Fc^{+}. This is consistent with the reported values.[4]

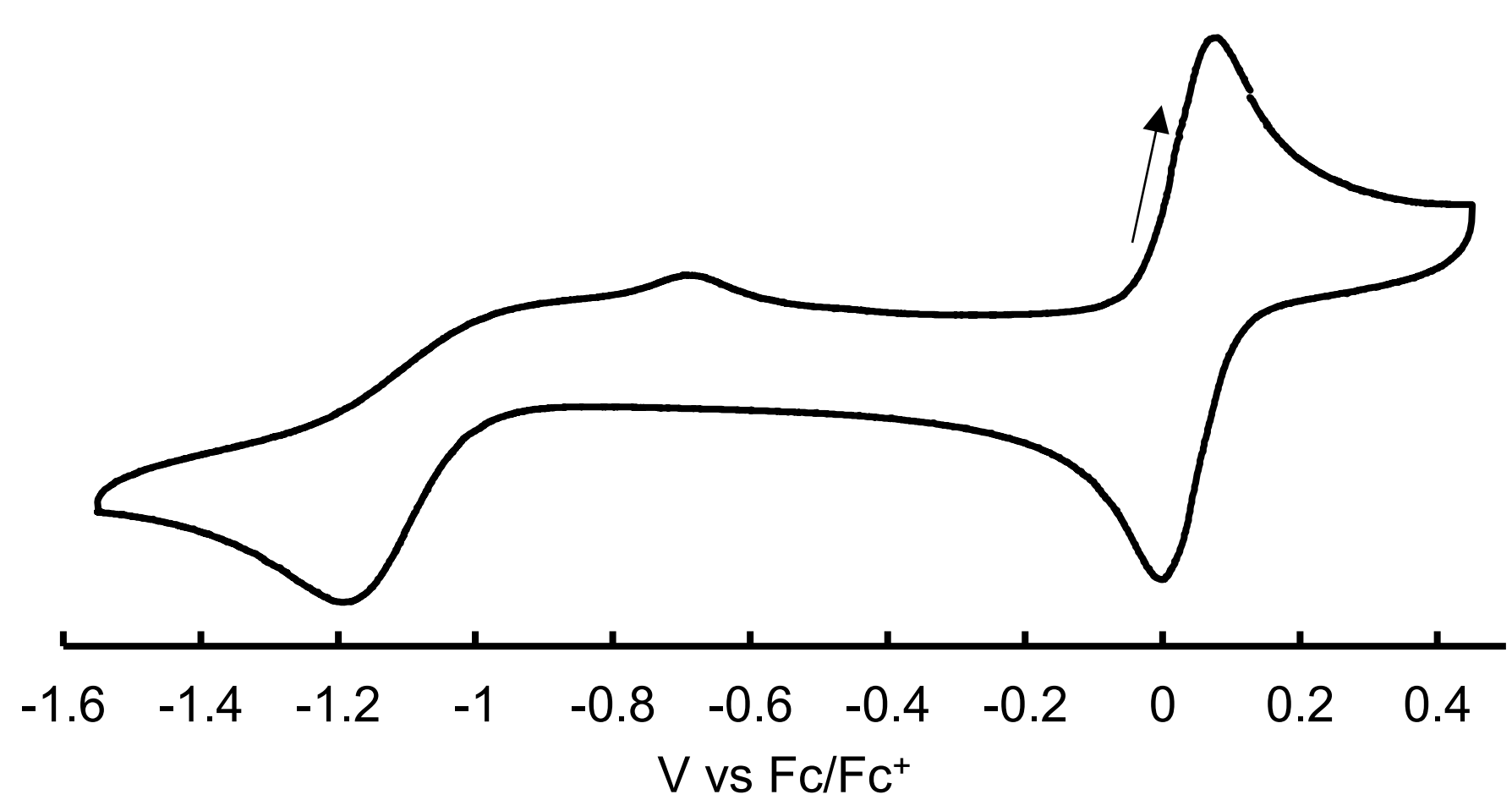

Figure S2. Cyclic voltammogram of **L**Cu^{II}Cl in DCM at 100 mV/s. $Cu^{I/II}$ peak potential = −1.21 V vs Fc/Fc^+, $Cu^{II/III}$ $E_{1/2}$ = 40 mV vs Fc/Fc^+. This is consistent with the reported values.[4]

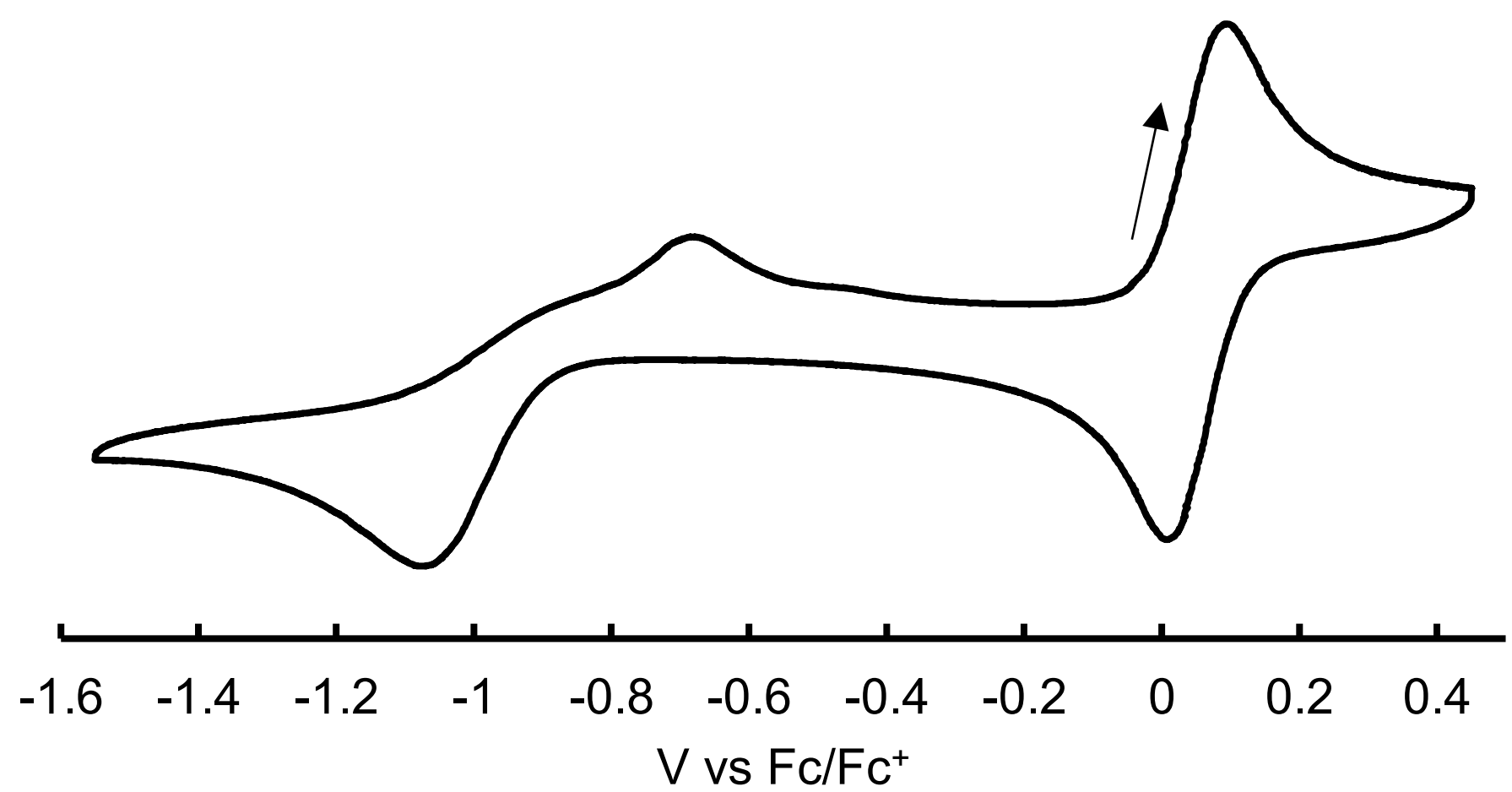

Figure S3. Cyclic voltammogram of **L**Cu^{II}Br in DCM at 100 mV/s. $Cu^{I/II}$ peak potential = −1.06 V vs Fc/Fc^+, $Cu^{II/III}$ $E_{1/2}$ = 50 mV vs Fc/Fc^+. This is consistent with the reported values.[4]

UV-vis spectroscopic studies

Beer's Law plots

UV-vis spectra were collected on an Agilent Cary 60 spectrophotometer outfitted with a Unisoku Unispeks cryostat (−100 °C to + 100 °C). For [**L**Cu^{III}X][SbF_6] complexes: dichloromethane solutions of [**L**Cu^{III}X][SbF_6] (0.075 mM, 0.1 mM, 0.125 mM, 0.15 mM, 0.175 mM and 0.2 mM) were made independently and placed in a quartz cuvette with septum attachment under a nitrogen atmosphere at room temperature. A UV-vis spectrum was taken of each concentration and plotted to obtain the extinction coefficient for each complex.

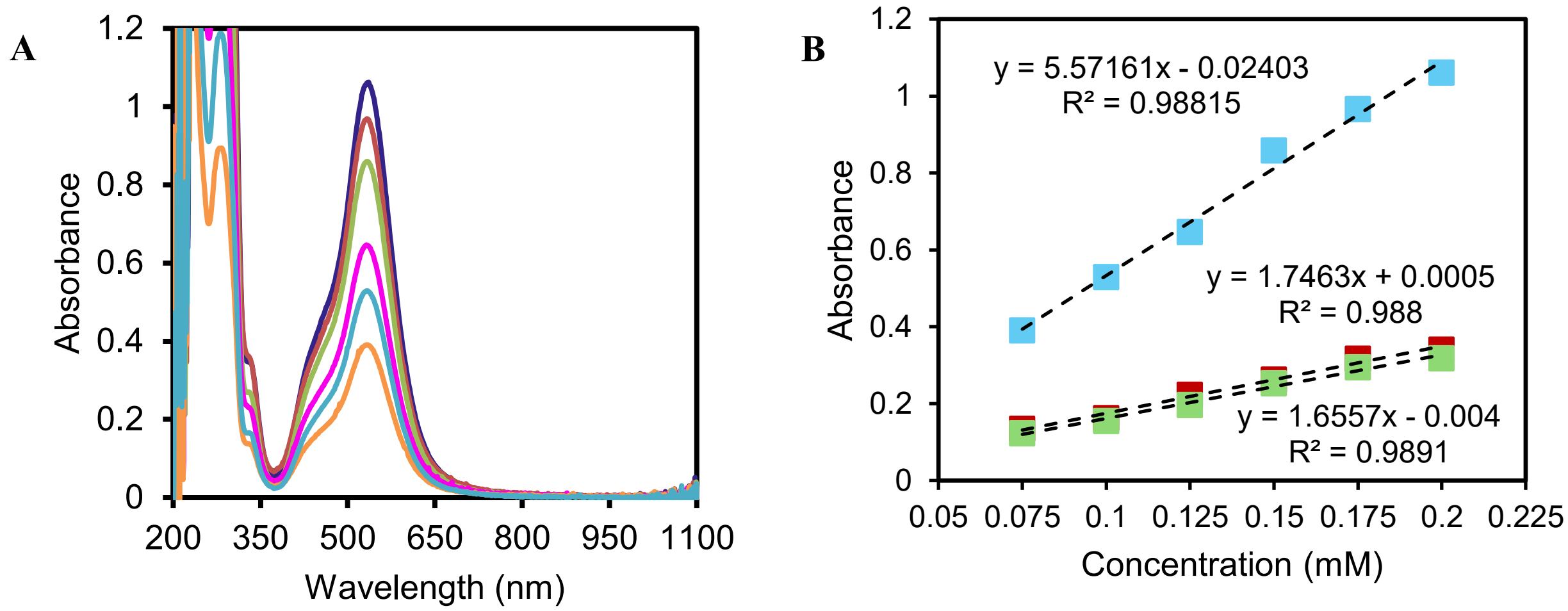

Figure S4. (A) UV-vis spectra obtained from various concentrations of [**L**Cu^{III}F][SbF_6] at 20 ºC in DCM. (B) Beer's law plot for [**L**Cu^{III}F][SbF_6]: 335 nm (ε = 1750 M^{-1} cm^{-1}), 426 nm (ε = 1660 M^{-1} cm^{-1}), 532 nm (ε = 5570 M^{-1} cm^{-1}).

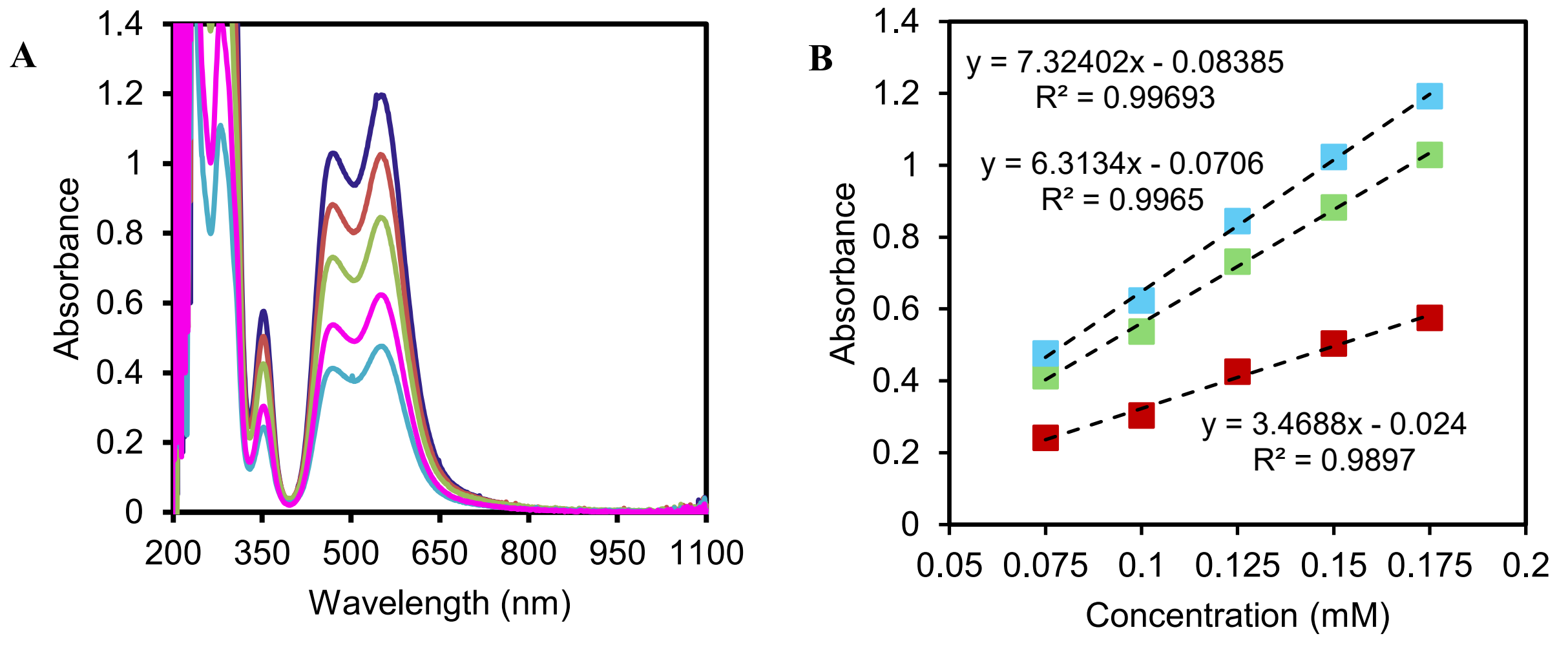

Figure S5. (A) UV-vis spectra obtained from various concentrations of [**L**Cu^{III}Cl][SbF_6] at 20 ºC in DCM. (B) Beer's law plot for [**L**Cu^{III}Cl][SbF_6]: 351 nm (ε = 3470 M^{-1} cm^{-1}), 469 nm (ε = 6310 M^{-1} cm^{-1}), 552 nm (ε = 7320 M^{-1} cm^{-1}).

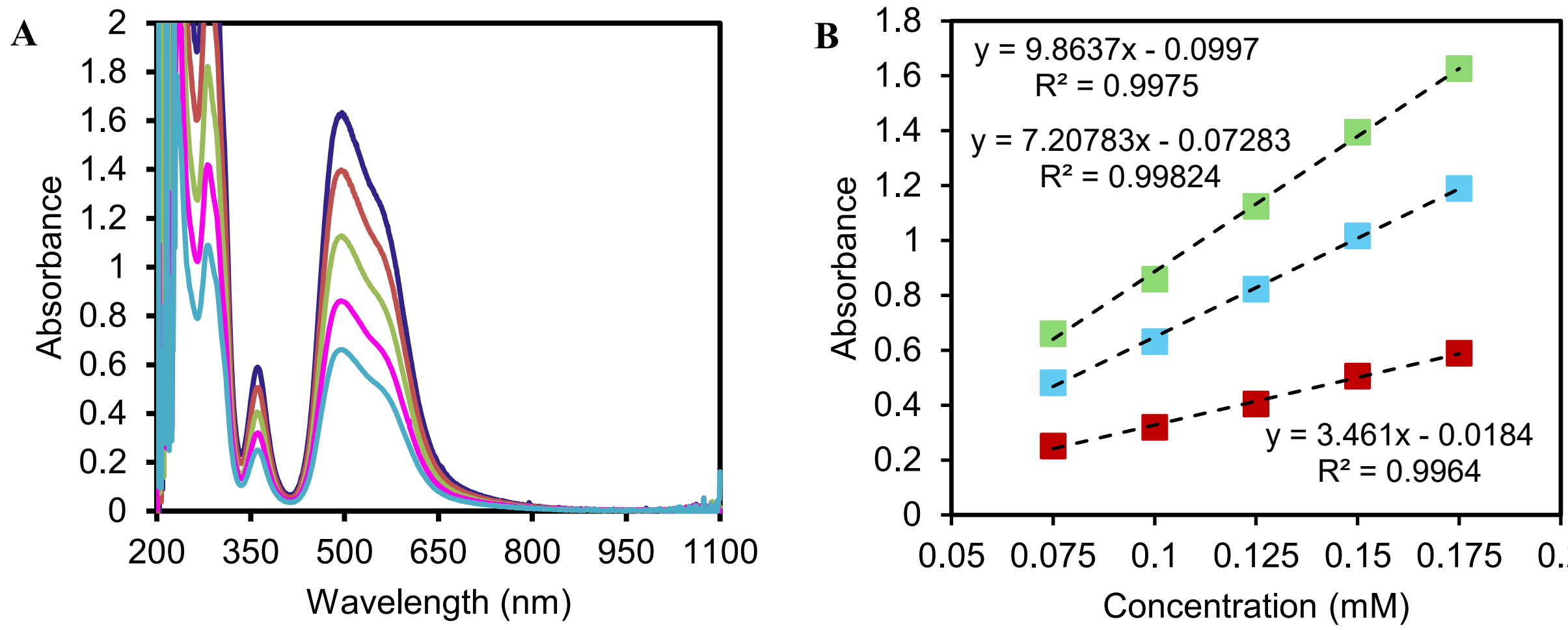

Figure S6. (A) UV-vis spectra obtained from various concentrations of [**L**Cu^{III}Br][SbF_6] at 20 ºC in DCM. (B) Beer's law plot for [**L**Cu^{III}Br][SbF_6]: 360 nm (ε = 3460 M^{-1} cm^{-1}), 493 nm (ε = 9860 M^{-1} cm^{-1}), 566 nm (ε = 7210 M^{-1} cm^{-1}).

IR Plots

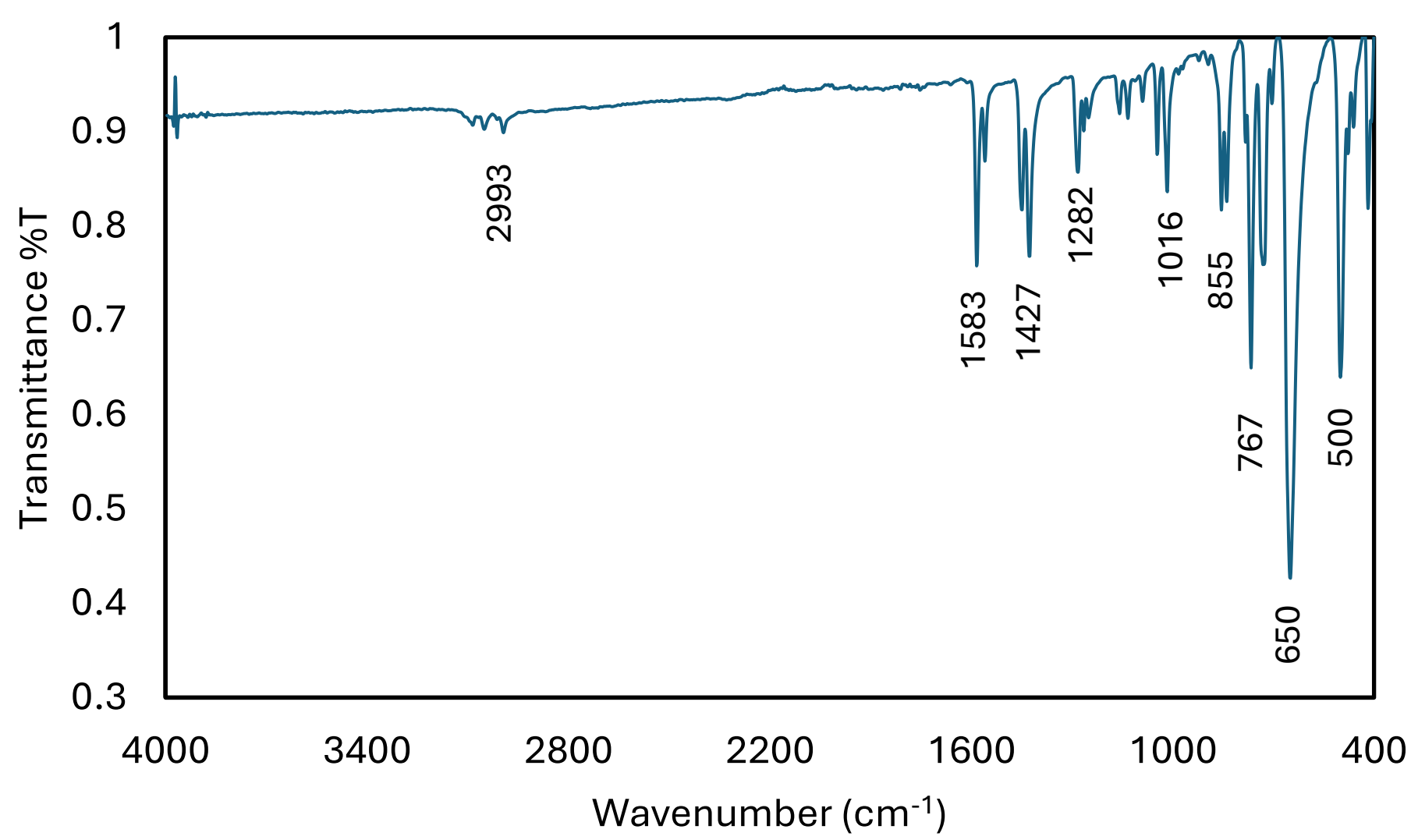

Figure S7. ATR-IR spectra of [**L**Cu^{III}F][SbF_6]

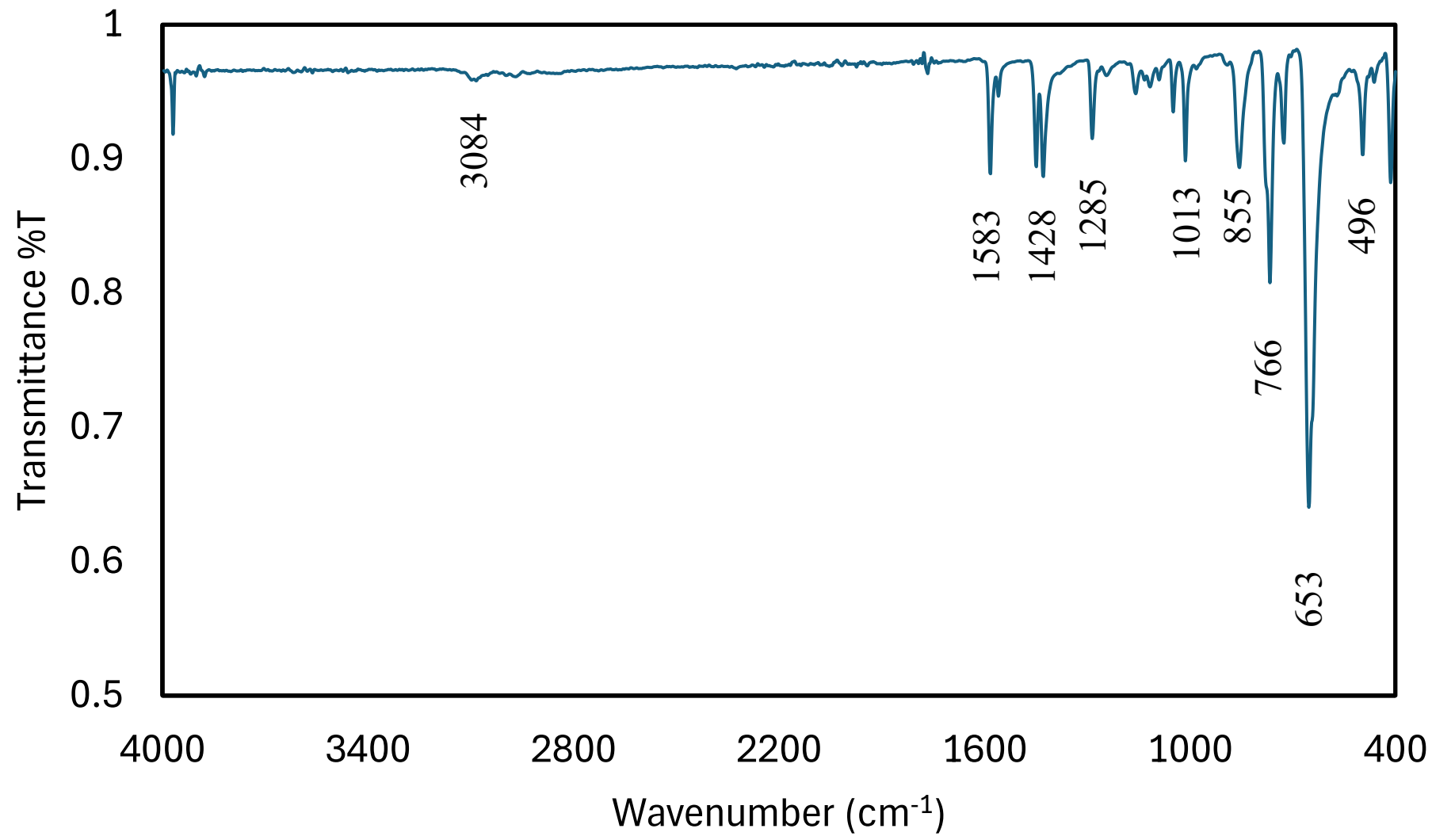

Figure S8. ATR-IR spectra of [**L**Cu^{III}Cl][SbF$_6$]

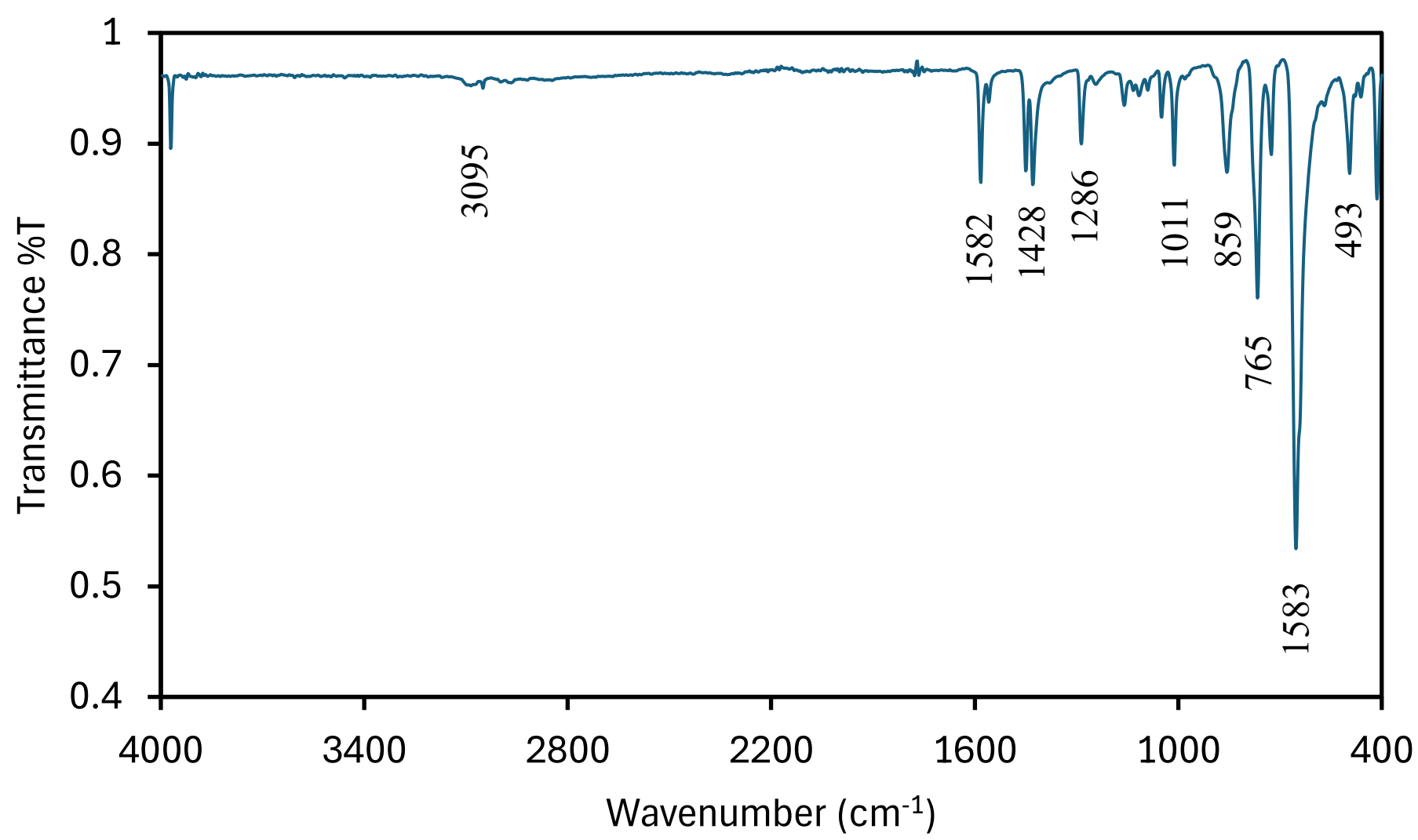

Figure S9. ATR-IR spectra of [**L**Cu^{III}Br][SbF$_6$]

Kinetic studies of HAT by LCu^{III}X

UV-vis spectra were collected on an Agilent Cary 60 spectrophotometer outfitted with a Unisoku Unispeks cryostat (−100 °C to + 100 °C). Stock solutions of **L**Cu^{II}F (3.0 mM in DCM), NAr$_3$[SbF$_6$] (3.0 mM in DCM) and DHA (300 mM in DCM) were made. To a septa cuvette, **L**Cu^{II}F (0.1 mL, 3.0 mM, 1.0 equiv.) solution was added and diluted to 2.8 mL then sealed before being transferred to the UV-vis. Then at 20 °C, NAr$_3$[SbF$_6$] (0.1 mL, 3.0 mM, 1.0 equiv.) was added to the cuvette to generate [**L**Cu^{III}F][SbF$_6$] in situ. After allowing the reaction to stir for 5 min, the DHA solution was added (0.1 mL, 300 mM, 100 equiv.). The decay of the absorption maximum of [**L**Cu^{III}F]$^+$ at 532 nm was monitored.

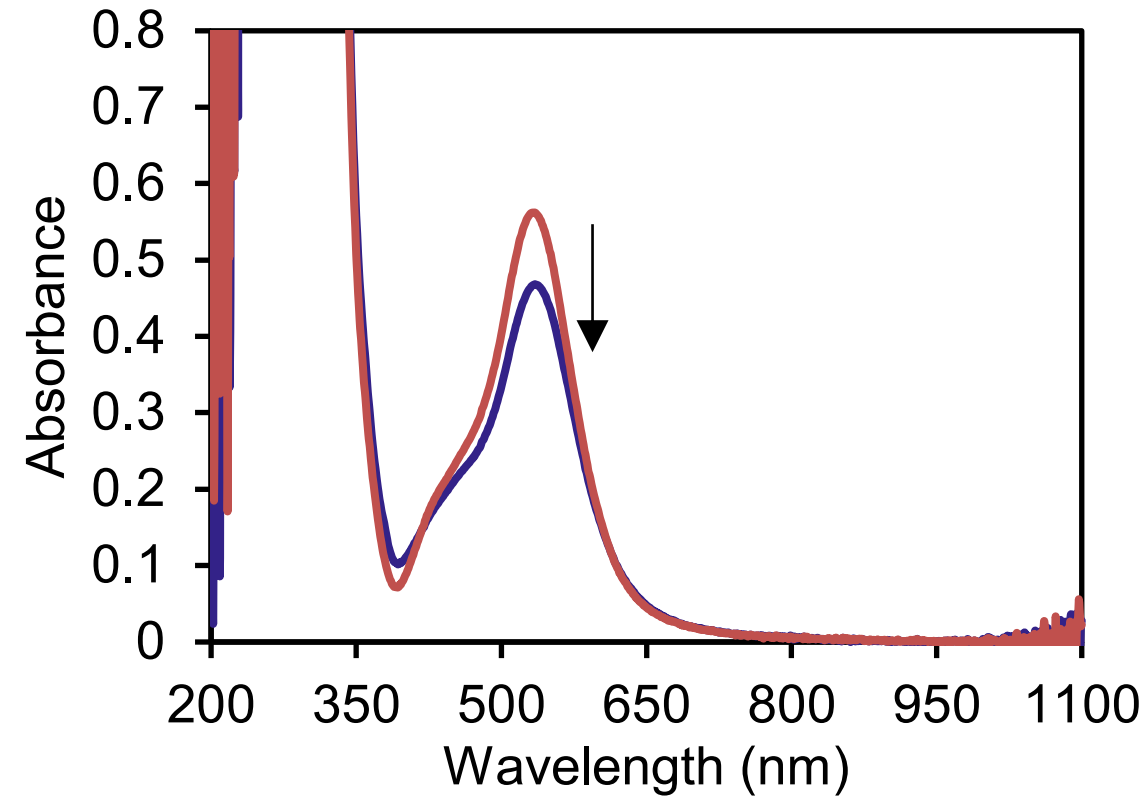

Figure S10. UV-vis traces of the reaction between [**L**Cu^{III}F][SbF_6] with an excess amount of DHA for 8 hours. The decay rate indicates that [**L**Cu^{III}F][SbF_6] is not reacting with DHA. Additionally, no anthracene is detected after the reaction using GCMS.

Thermal decomposition of [LCu^{III}X][SbF$_6$] complexes

A dichloromethane solution of [**L**Cu^{III}X][SbF_6] (3.000 mL, 0.10 mM, 0.3 μmol) was placed in a quartz cuvette with Schlenk attachment. The cryostat was heated to 40 °C. Once the temperature stabilized at 40 °C, the cuvette was inserted into the cryostat and data collection was initiated.

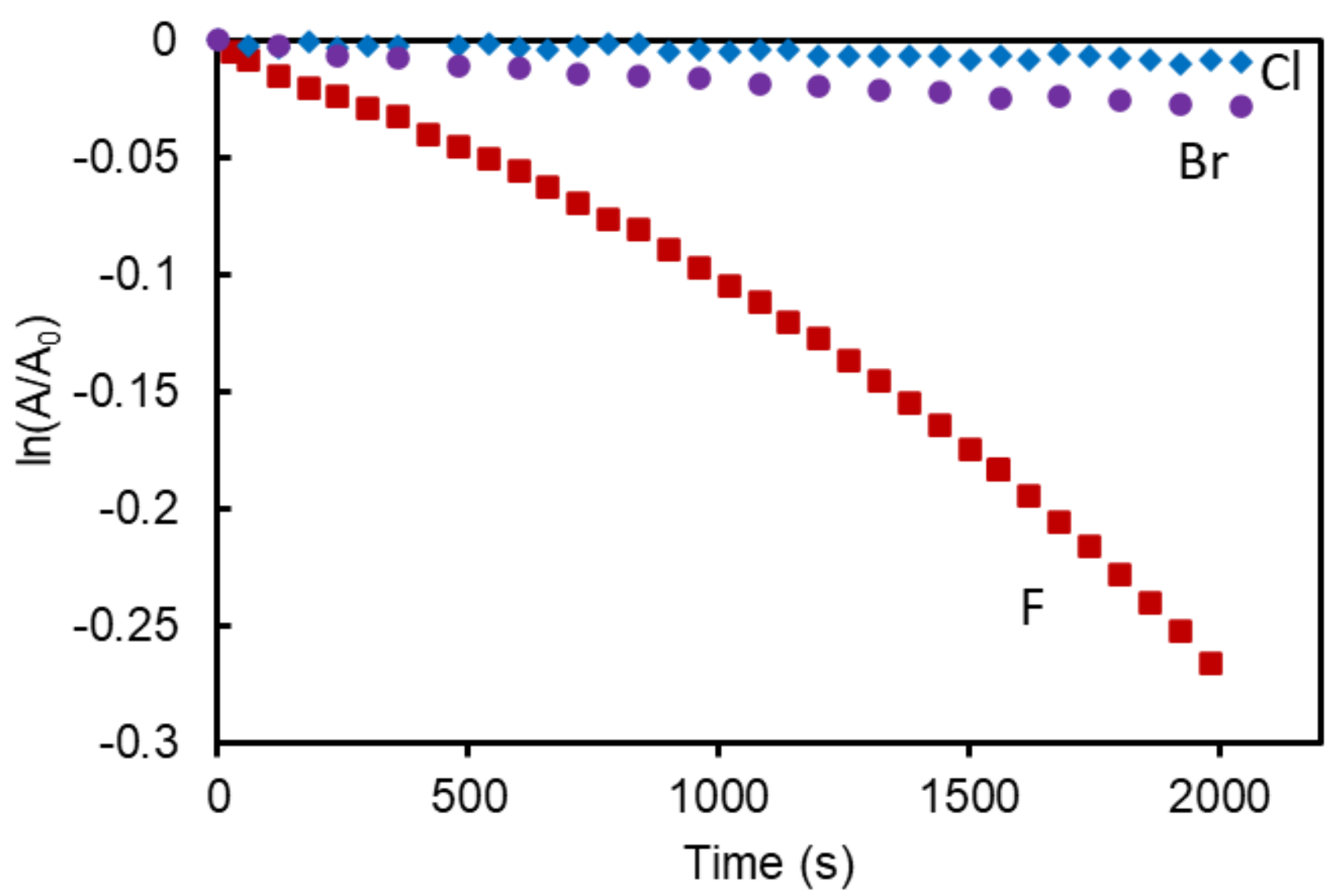

Figure S11. Overlay of kinetic traces of first order self-decay of [**L**Cu^{III}F][SbF_6] (dark red squares, λ_{max} = 532 nm), [**L**Cu^{III}Cl][SbF_6] (blue diamonds, λ_{max} = 552 nm), [**L**Cu^{III}Br][SbF_6] (purple circles, λ_{max} = 556 nm) at 40 °C in CDM. [**L**Cu^{III}F][SbF_6]: $k_{obs} = 1.29 \times 10^{-4}$ s^{-1} (R^2 = 0.979); [**L**Cu^{III}Cl][SbF_6]: $k_{obs} = 3.78 \times 10^{-6}$ s^{-1} (R^2 = 0.973); [**L**Cu^{III}Br][SbF_6]: $k_{obs} = 7.66 \times 10^{-6}$ s^{-1} (R^2 = 0.984).

Estimation of Lewis acidity using a modified Guttman-Beckett method

A stock solution of triphenylphosphine oxide (17.6 mM in CD_2Cl_2, 1.1 equiv.), [**L**Cu^{III}F][SbF_6] (16.0 mM in CD_2Cl_2, 1.0 equiv.), [**L**Cu^{III}Cl][SbF_6] (16.0 mM in CD_2Cl_2, 1.0 equiv.), [**L**Cu^{III}Br][SbF_6] (16.0 mM in CD_2Cl_2, 1.0 equiv.) and BCF (16.0 mM in CD_2Cl_2, 1.0 equiv.). To an NMR tube, the compound of interest ([**L**Cu^{III}F][SbF_6], [**L**Cu^{III}Cl][SbF_6], [**L**Cu^{III}Br][SbF_6] or BCF) (0.300 mL, 16.0 mM, 1.0 equiv.) and triphenylphosphine oxide (0.300 mL, 17.6 mM, 1.1 equiv.) were added. The resulting solution was analyzed by ^{31}P NMR Spectroscopy. The samples were referenced to free triphenylphosphine oxide using a sealed capillary tube.

A stock solution of triphenylphosphine oxide (17.6 mM in CD_2Cl_2, 1.1 equiv.), [**L**Cu^{II}MeCN][PF_6] (32.0 mM in CD_2Cl_2, 1.0 equiv.), [**PDA**Cu^{II}F][TBA] (32.0 mM in CD_2Cl_2, 1.0 equiv.), and [NAr_3][SbF_6] (32.0 mM in CD_2Cl_2, 1.0 equiv.). To an NMR tube, copper complex ([**L**CuMeCN][PF_6] or [**PDA**Cu^{II}F][TBA]) (0.150 mL, 32.0 mM, 1.0 equiv.), [NAr_3][SbF_6] (0.150 mL, 32.0 mM, 1.0 equiv.) and triphenylphosphine oxide (0.300 mL, 17.6 mM, 1.1 equiv.) were added generating the Cu(III) complexes in situ. The resulting solutions were analyzed by ^{31}P NMR Spectroscopy. The samples were referenced to free triphenylphosphine oxide using a sealed capillary tube.

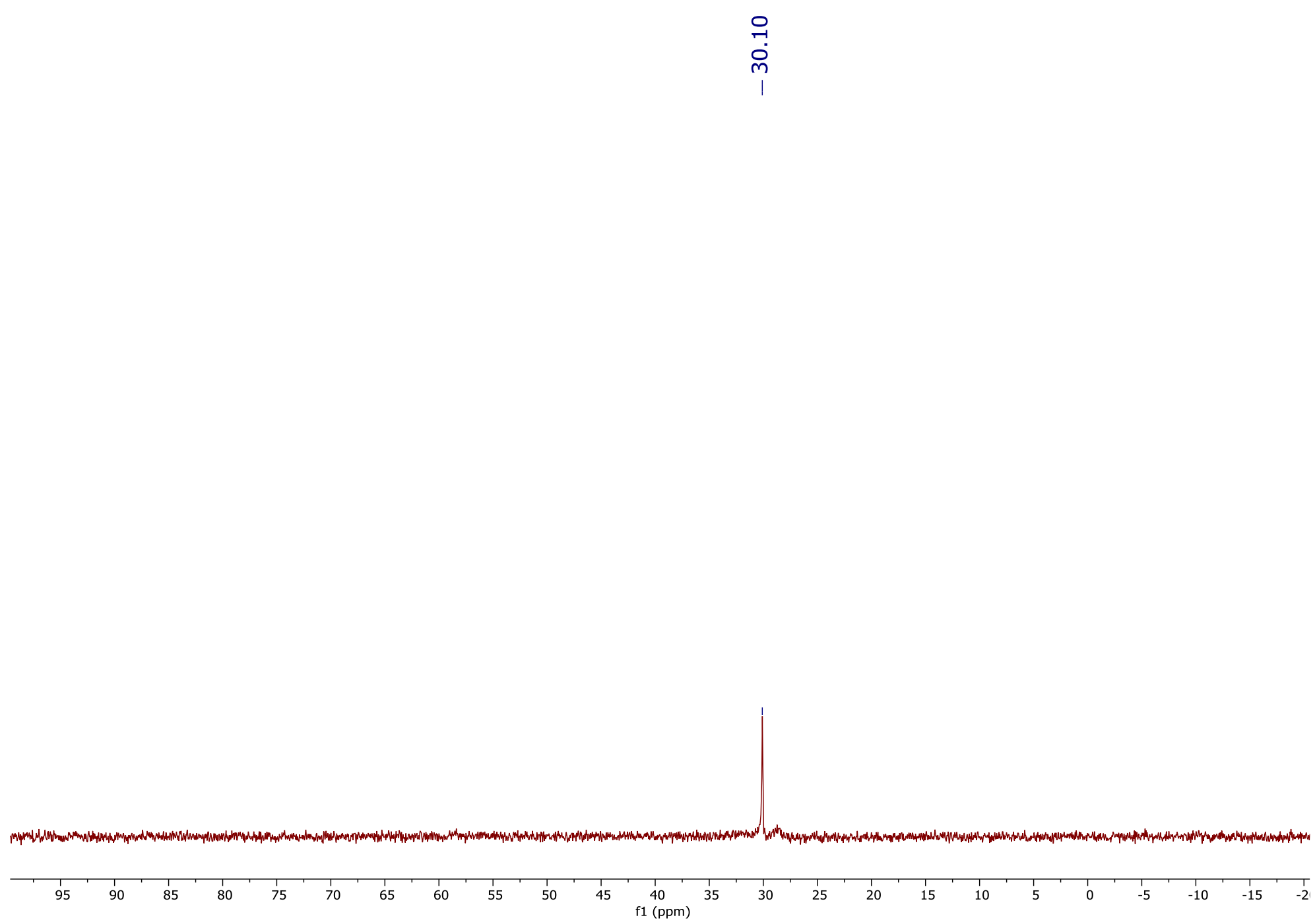

Figure S12. $^{31}P\{^1H\}$ NMR spectrum (242.95 MHz, CD_2Cl_2) of a mixture of [**L**Cu^{III}F][SbF_6] and triphenylphosphine oxide. The ^{31}P resonance of triphenylphosphine oxide resonance did not shift from the standard (30.10 ppm).

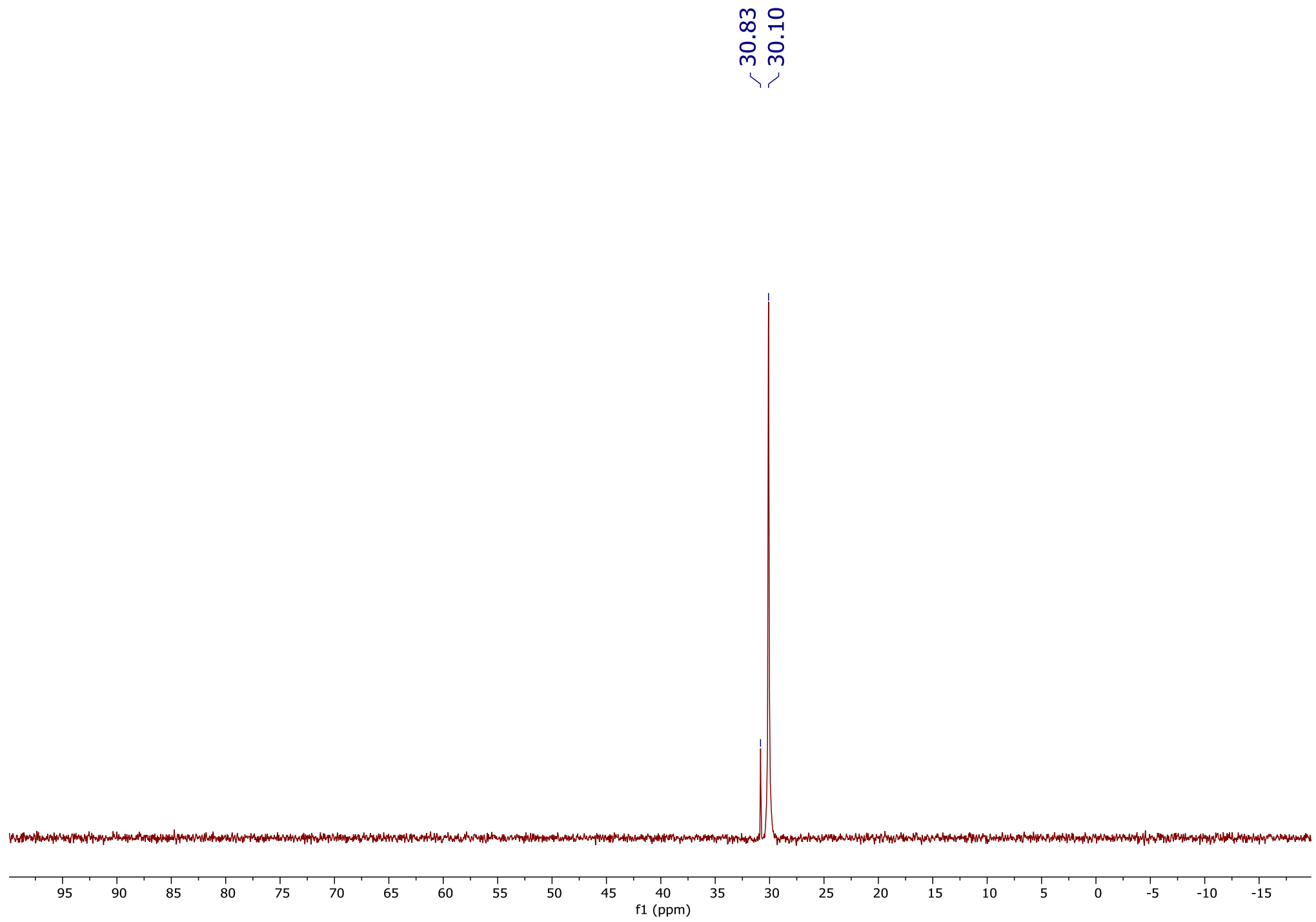

Figure S13. $^{31}P\{^{1}H\}$ NMR spectrum (242.95 MHz, CD_2Cl_2) of a mixture of [$\mathbf{L}Cu^{III}Cl$][SbF_6] and triphenylphosphine oxide. The ^{31}P resonance of triphenylphosphine oxide shifts by 0.73 ppm from the standard (30.10 ppm) due to the presence of [$\mathbf{L}Cu^{III}Cl$][SbF_6].

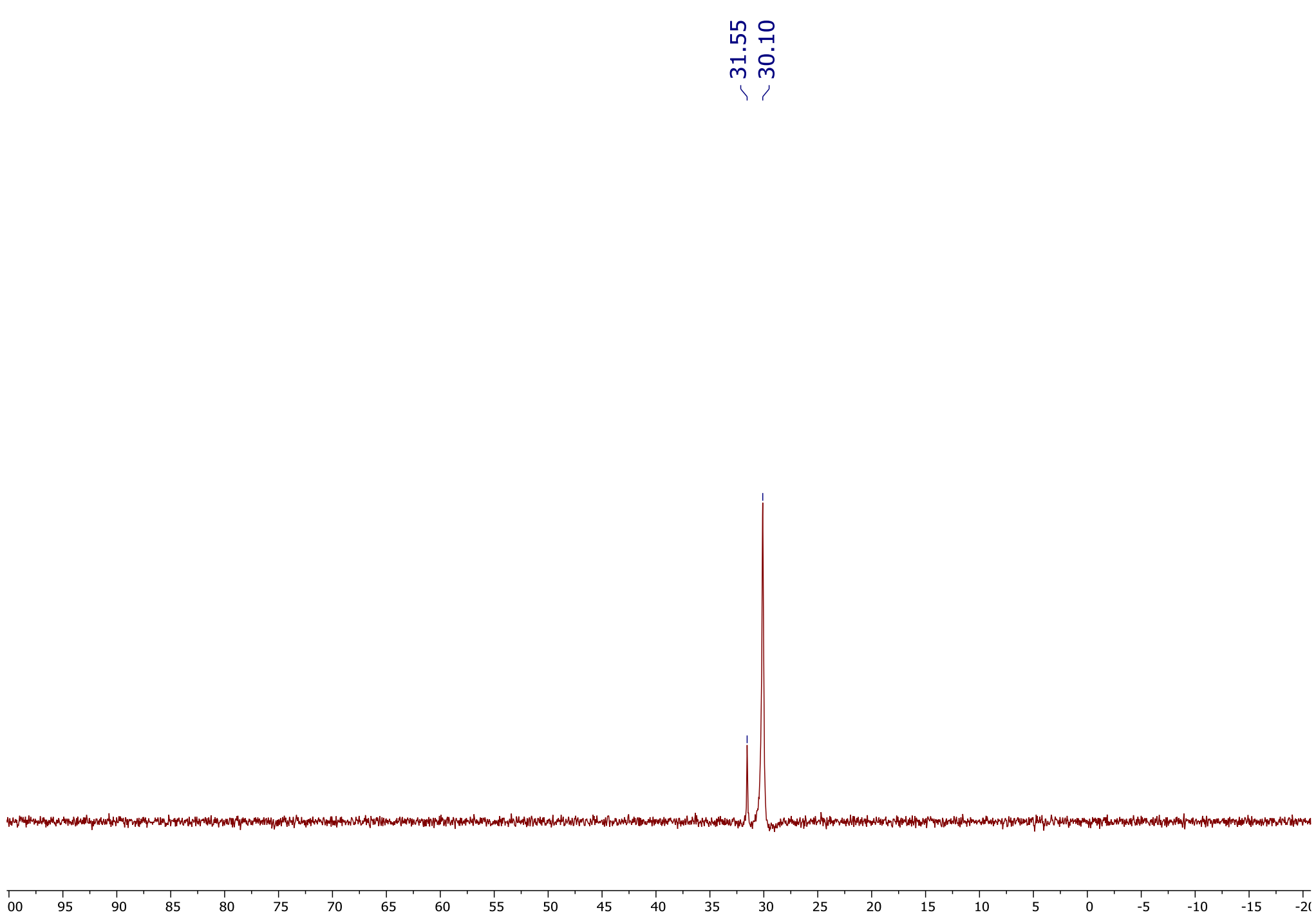

Figure S14. $^{31}P\{^{1}H\}$ NMR spectrum (242.95 MHz, CD_2Cl_2) of a mixture of [**L**Cu^{III}Br][SbF_6] and triphenylphosphine oxide. The ^{31}P resonance of triphenylphosphine oxide shifts by 1.45 ppm from the standard (30.10 ppm) due to the presence of [**L**Cu^{III}Br][SbF_6].

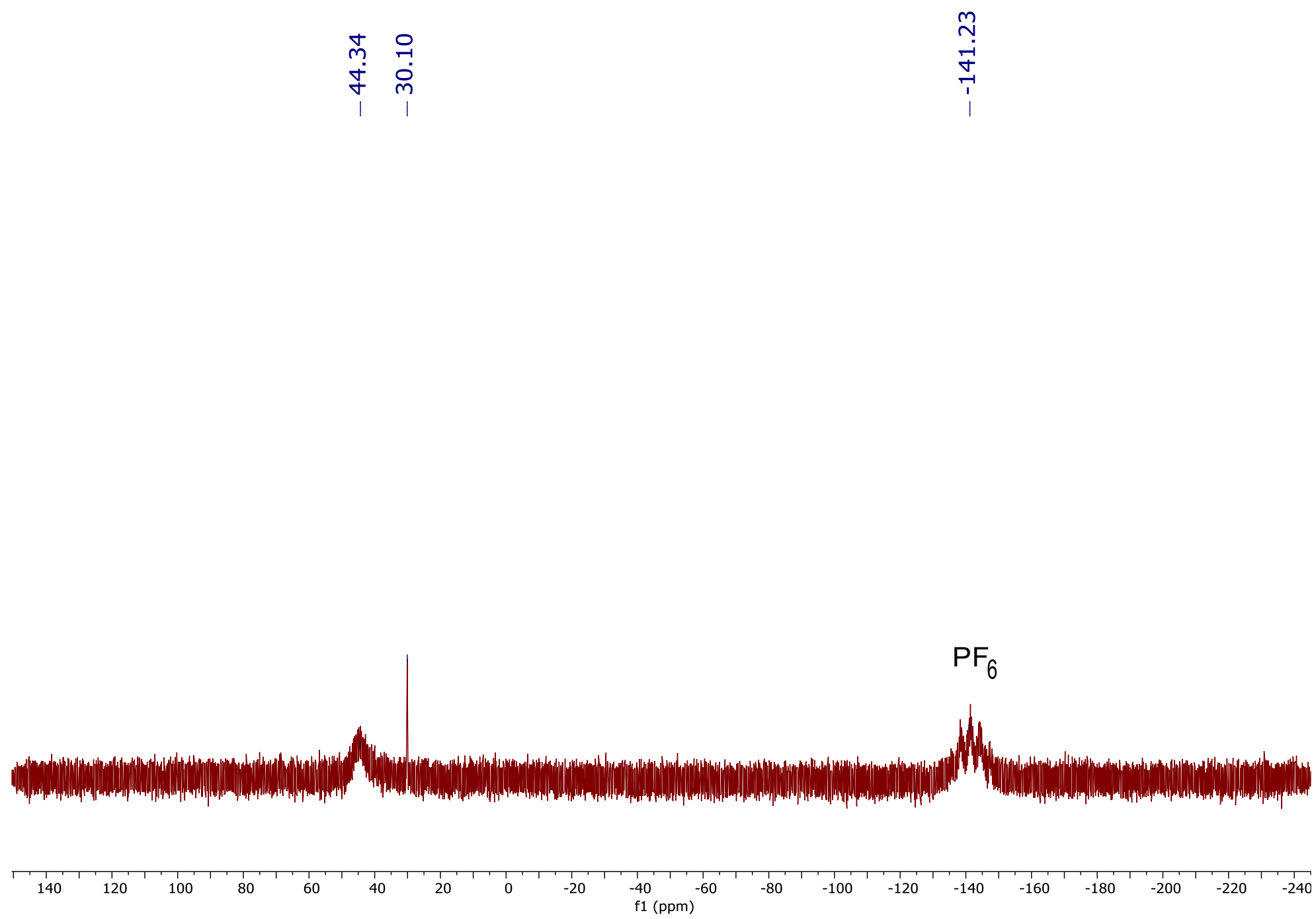

Figure S15. $^{31}P\{^{1}H\}$ NMR spectrum (242.95 MHz, CD_2Cl_2) of a mixture of [**L**Cu^{III}MeCN][PF_6][SbF_6] and triphenylphosphine oxide. The ^{31}P NMR resonance of triphenylphosphine oxide shifts by 14.2 ppm from the standard (30.10 ppm) due to the presence of [**L**Cu^{III}MeCN][PF_6][SbF_6].

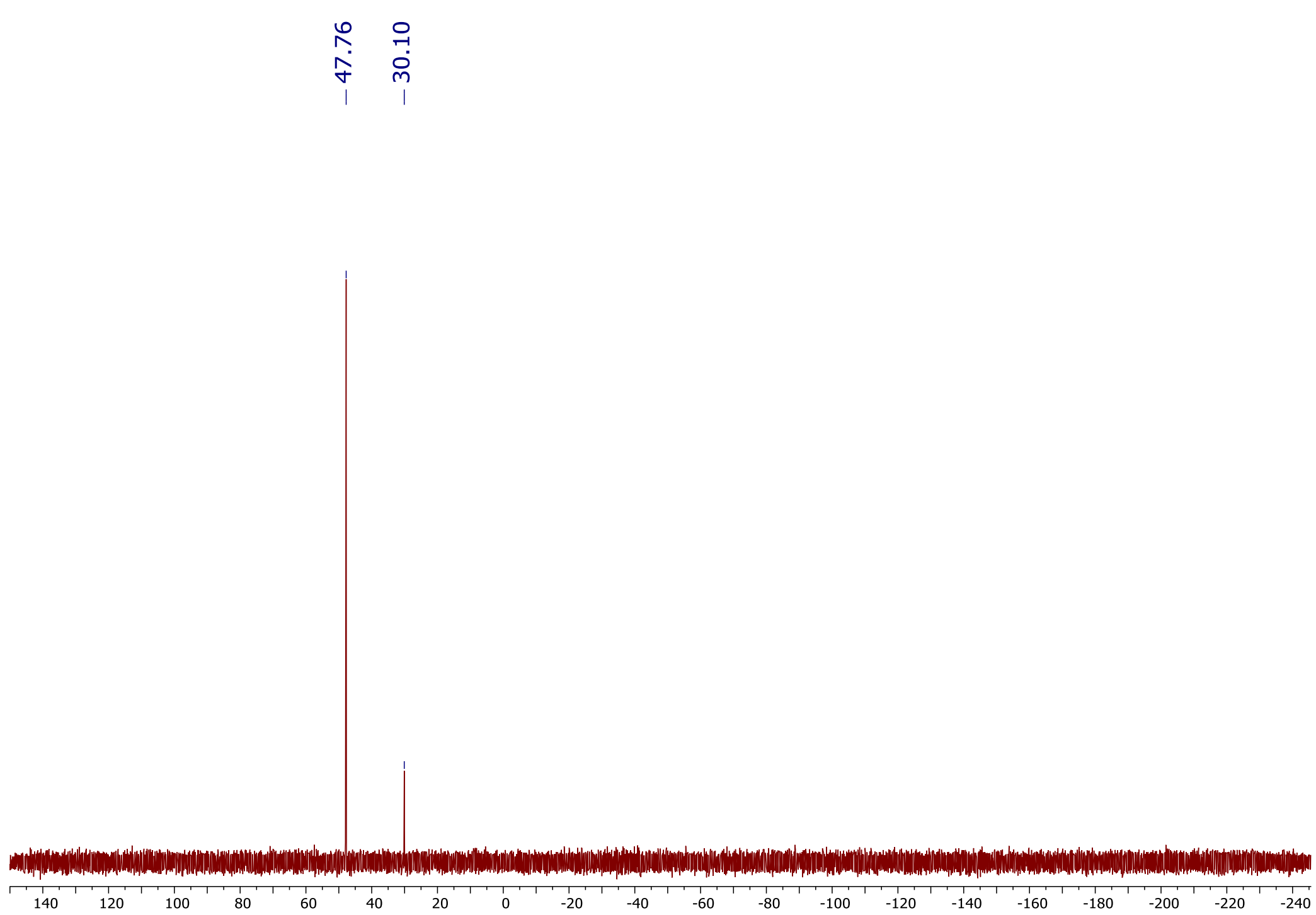

Figure S16. $^{31}P\{^{1}H\}$ NMR spectrum (242.95 MHz, CD_2Cl_2) of a mixture of BCF and triphenylphosphine oxide. The ^{31}P NMR resonance of triphenylphosphine oxide shifts by 17.65 ppm from the standard (30.10 ppm) due to the presence of BCF.

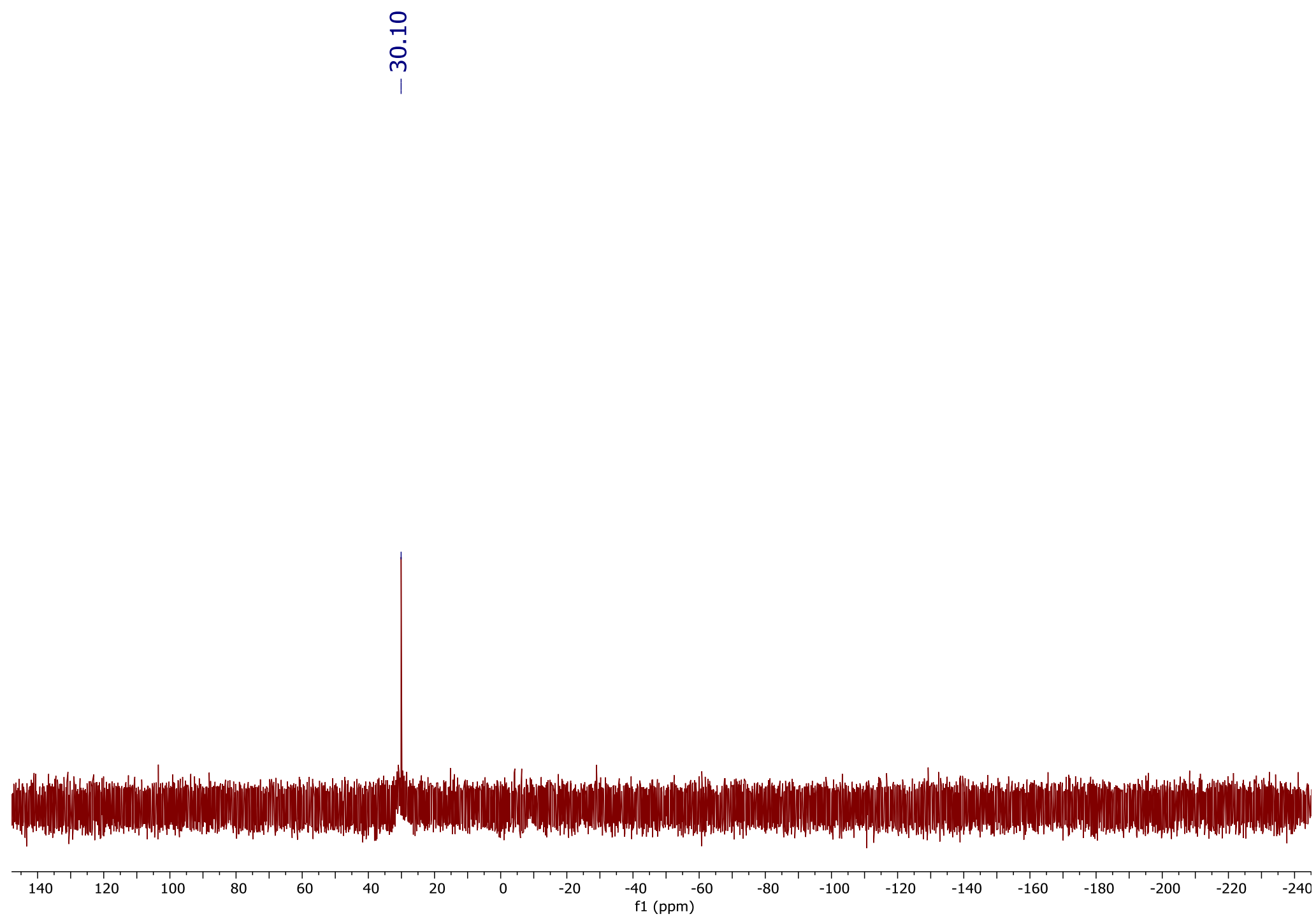

Figure S17. $^{31}P\{^{1}H\}$ NMR spectrum (242.95 MHz, CD_2Cl_2) of a mixture of **PDA**Cu(III)F and triphenylphosphine oxide. The ^{31}P NMR resonance of triphenylphosphine oxide resonance did not shift from the standard (30.10 ppm). The complex is observed to decompose immediately upon addition of triphenylphosphine oxide.

A stock solution of triphenylphosphine oxide (48 mM in CD_2Cl_2, 1.1 equiv.) and [**L**Cu^{III}Br][SbF_6] (16.0 mM in CD_2Cl_2, 1.0 equiv.) were made. To an NMR tube, [**L**Cu^{III}Br][SbF_6] and triphenylphosphine oxide (0.300 mL, 48 mM, 3.0 equiv.) were added. The resulting solution was analyzed by ^{31}P NMR Spectroscopy. The samples were referenced to free triphenylphosphine oxide using a sealed capillary tube. The use of excess triphenylphosphine oxide does not change the observed ^{31}P NMR shift.

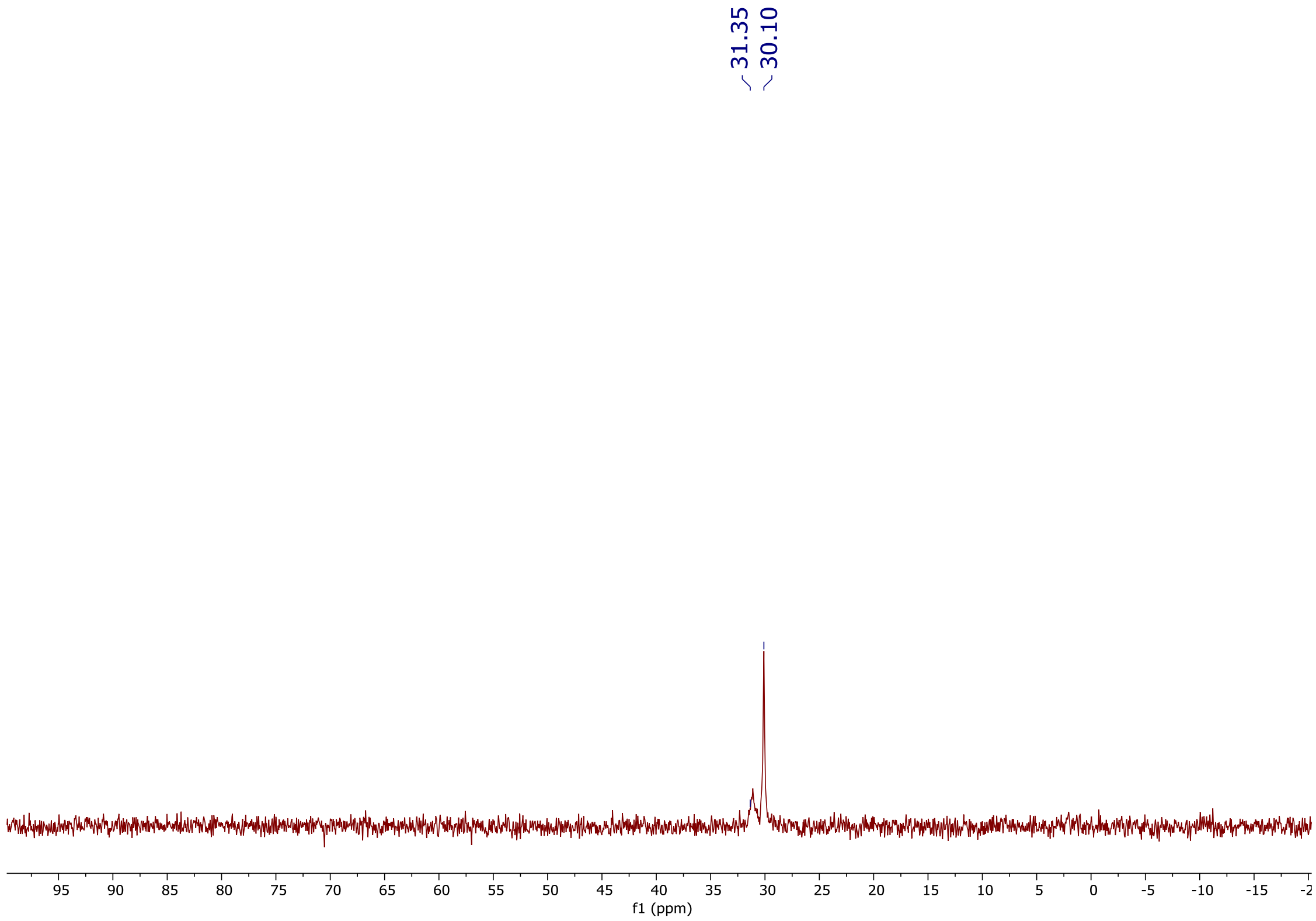

Figure S18. $^{31}P\{^{1}H\}$ NMR spectrum (242.95 MHz, CD_2Cl_2) of a mixture of [**L**Cu^{III}Br][SbF_6] and triphenylphosphine oxide (3 equiv.). The ^{31}P NMR resonance of triphenylphosphine oxide shifts by 1.25 ppm from the standard (30.10 ppm) due to the presence of [**L**Cu^{III}Br][SbF_6].

Procedure for trityl radical capture with [LCu^{III}X]$^{+}$ complexes

*Procedure for radical capture with [**L**Cu^{III}X][SbF$_6$]:* A CD_2Cl_2 solution of [**L**Cu^{III}F][SbF_6] (0.250 mL, 10 mM) was added to an NMR tube inside an N_2 glovebox. Then a solution of Gomberg's dimer (0.250 mL, 5 mM) was added. The tube was inverted and allowed to sit at room temperature for 30 minutes prior to NMR analysis. ^{19}F NMR was used to quantify the yield of Ph_3C-F due to its instability on silica gel column.

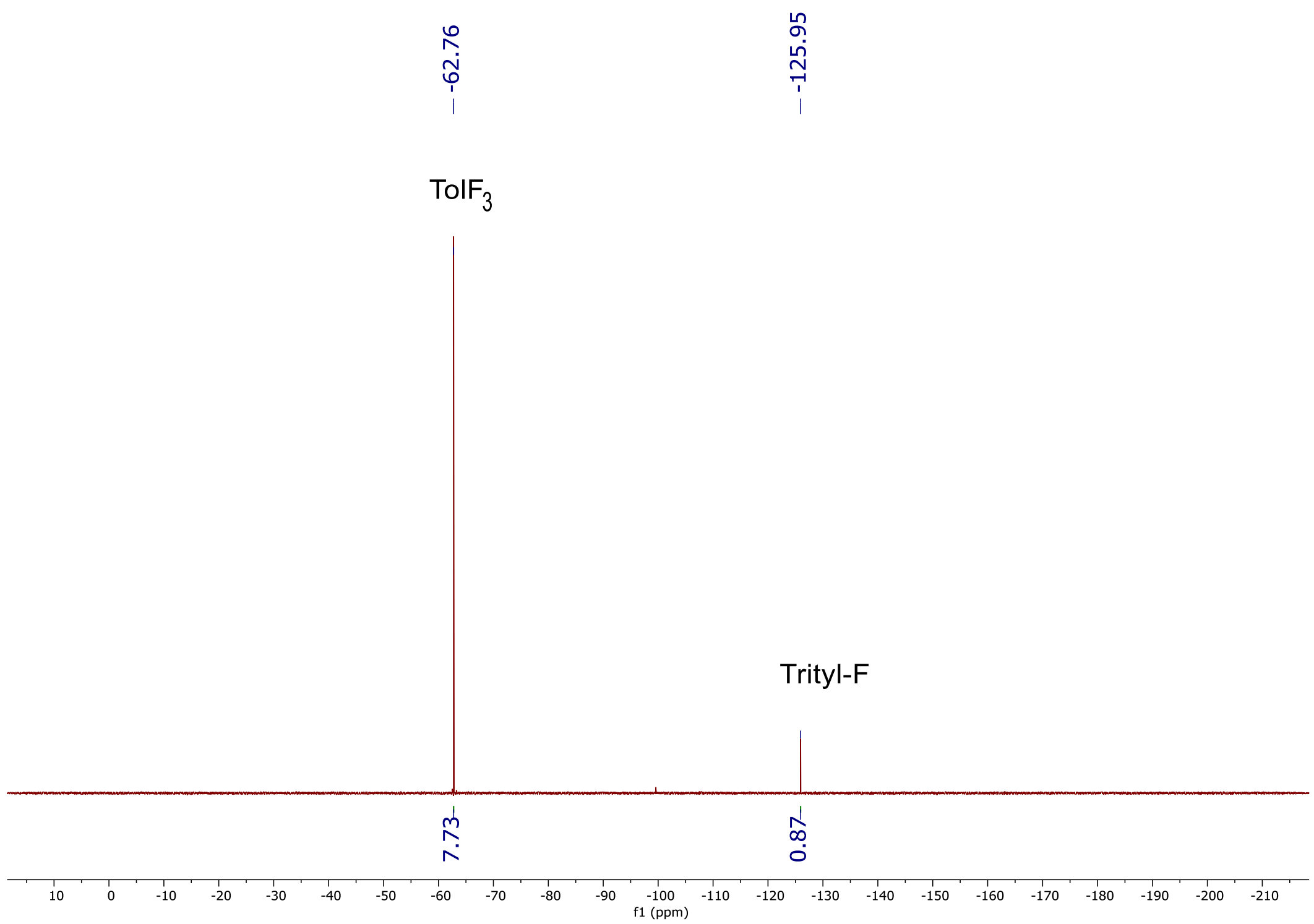

Figure S18. $^{19}F\{^1H\}$ NMR (565 MHz, CD_2Cl_2) of the reaction between [**L**Cu^{III}F][SbF_6] and 0.5 equivalents of Gomberg's dimer, revealing the formation of trityl fluoride at 125.9 ppm.[7] A ^{19}F NMR yield of 87% was obtained. The sample was referenced to an internal standard of trifluorotoluene ($TolF_3$).

Evaluation of halide substitution at [LCu^{III}F]$^+$

UV-vis spectra were collected on an Agilent Cary 60 spectrophotometer outfitted with a Unisoku Unispeks cryostat (−100 °C to + 100 °C). Stock solutions of [**L**Cu^{III}F]$^+$ (3.0 mM in DCM) and TBACl (3.0 mM in DCM) were made. To a septum cuvette, [**L**Cu^{III}F]$^+$ (0.1 mL, 3.0 mM, 1.0 equiv.) solution was added and diluted to 2.8 mL then sealed before being transferred to the UV-vis. Then at 20 °C, TBACl (0.1 mL, 3.0 mM, 1.0 equiv.) was added to the cuvette. This caused an immediate reduction of [**L**Cu^{III}F]$^+$ to form **L**Cu^{II}Cl

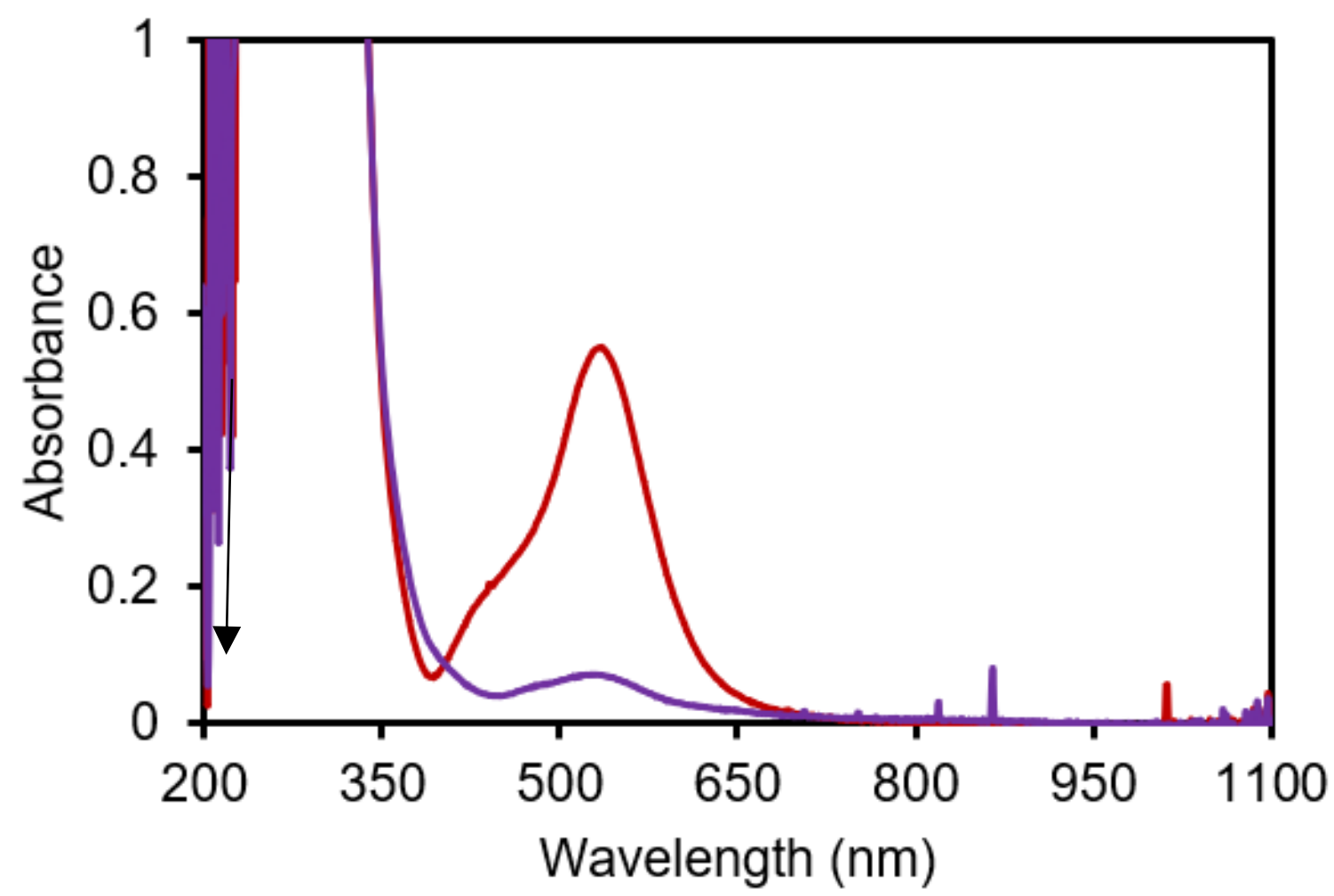

Figure S19. UV-vis traces of the reaction between [**L**Cu^{III}F][SbF_6] and TBACl.

Stock solutions of [**L**Cu^{III}F]$^+$ (3.0 mM in DCM) and cumyl alcohol (300.0 mM in DCM) were made. To a septa cuvette, [**L**Cu^{III}F]$^+$ (0.1 mL, 3.0 mM, 1.0 equiv.) solution was added and diluted to 2.5 mL then sealed before being transferred to the UV-vis. Then at 20 °C, cumyl alcohol (0.1 mL, 300.0 mM, 100.0 equiv.) was added to the cuvette, this was performed 5 times resulting in 500 equiv. of cumyl alcohol being added. This caused only a serial dilution of [**L**Cu^{III}F]$^+$.

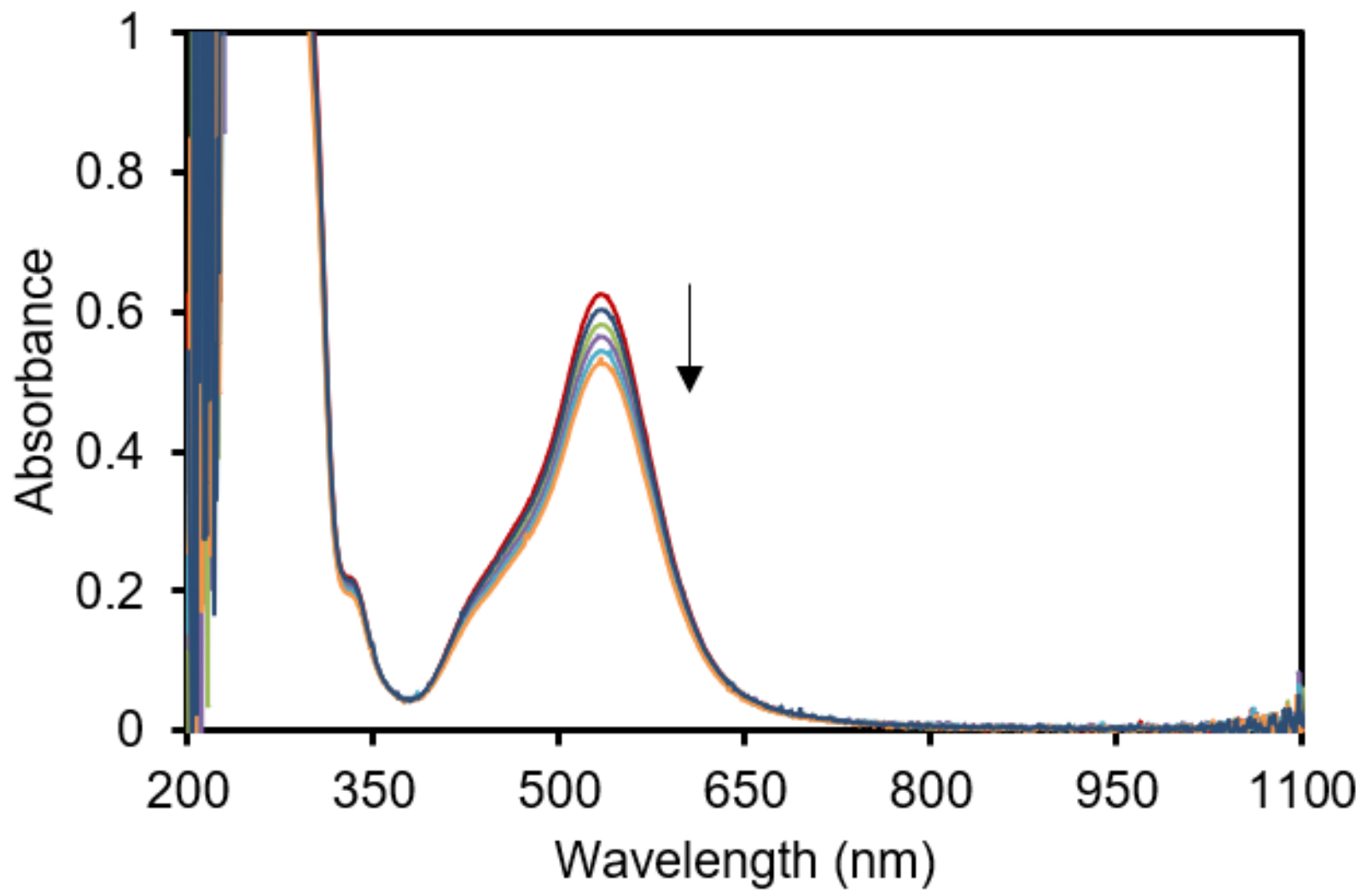

Figure S20. UV-vis traces of the addition of cumyl alcohol to [**L**Cu^{III}F][SbF$_6$].

Procedures for deoxyhalogentation of alcohols

Optimization of deoxyfluorination of alcohols with [LCu^{III}F]$^+$

To a 10 mL screw cap test tube equipped with a stir bar, [**L**Cu^{III}F][SbF$_6$] (16.5 mg, 0.0250 mmol, 1.00 equiv.) and cumyl alcohol (34.0 mg, 0.250 mmol, 10.0 equiv.) were added. The solids were dissolved in dichloroethane (2 mL). The reaction was stirred at 100 °C for 10 minutes. The reactions were stopped when the deep red color of the [**L**Cu^{III}F]$^+$ disappeared. Yields were determined using ^{19}F NMR with trifluorotoluene as the internal standard.

Table S1. Reaction optimization for the reaction between [**L**Cu^{III}F][SbF$_6$] and cumyl alcohol. Yields were determined by ^{19}F NMR.

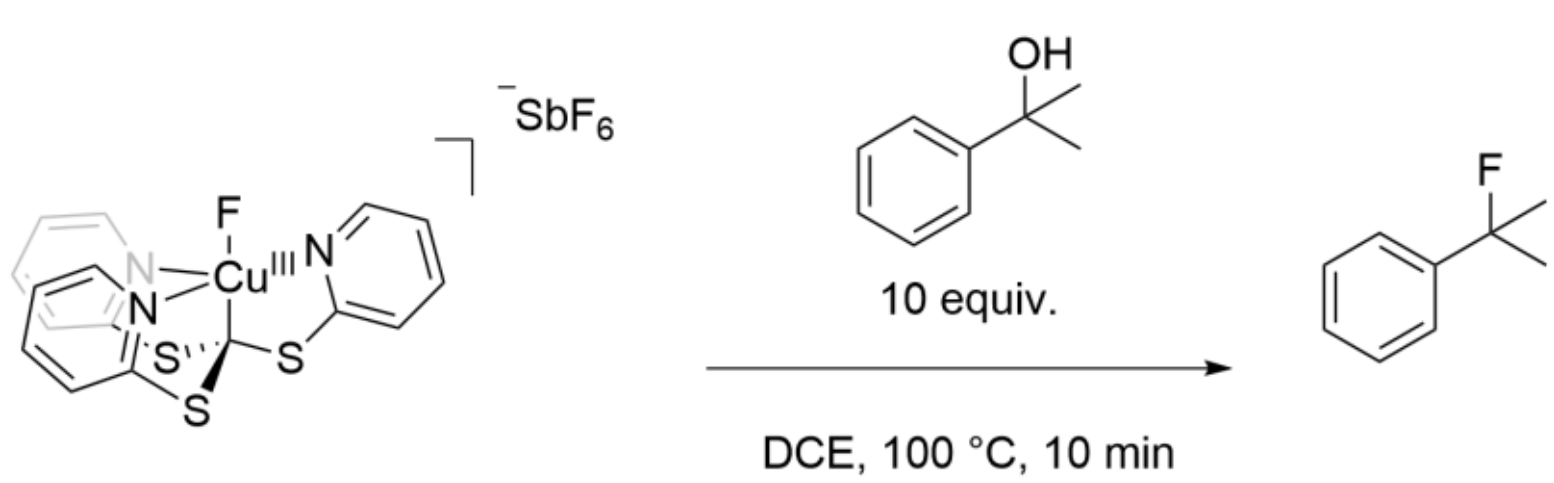

Entry	Deviation from standard conditions	Yield%
**1**	**None**	**40**
2	1 equiv. of cumyl alcohol	0
3	2 equiv. of cumyl alcohol	0
4	5 equiv. of cumyl alcohol	22
5	THF instead of DCE	0
6	MeCN instead of DCE	0
7	Benzene instead of DCE	0
8	1,4-dioxane instead of DCE	0
9	MeOH instead of DCE	0
10	60 °C	27
11	70 °C	33
12	80 °C	35
13	90 °C	33
14	110 °C	31

Optimization of deoxyfluorination of alcohols with $[\mathbf{L}Cu^{III}F]^+$ and additional fluorine Source

To a 10 mL screw cap test tube equipped with a stir bar, $[\mathbf{L}Cu^{III}F][SbF_6]$ (16.5 mg, 0.0250 mmol, 1.00 equiv.), cumyl alcohol (34.0 mg, 0.250 mmol, 10.0 equiv.) and $TBAPF_6$ (48.0 mg, 0.125 mmol, 5.00 equiv.) were added. The solids were dissolved in dichloroethane (2 mL). The reaction was stirred at 100 °C for 20 minutes. The reactions were stopped when the deep red color of the $[\mathbf{L}Cu^{III}F]^+$ disappeared. Yields were determined using ^{19}F NMR with trifluorotoluene as the internal standard.

Table S2. Reaction optimization for the reaction between [**L**Cu^{III}F][SbF_6] and cumyl alcohol with additional Fluorine sources. Yields were determined by ^{19}F NMR.

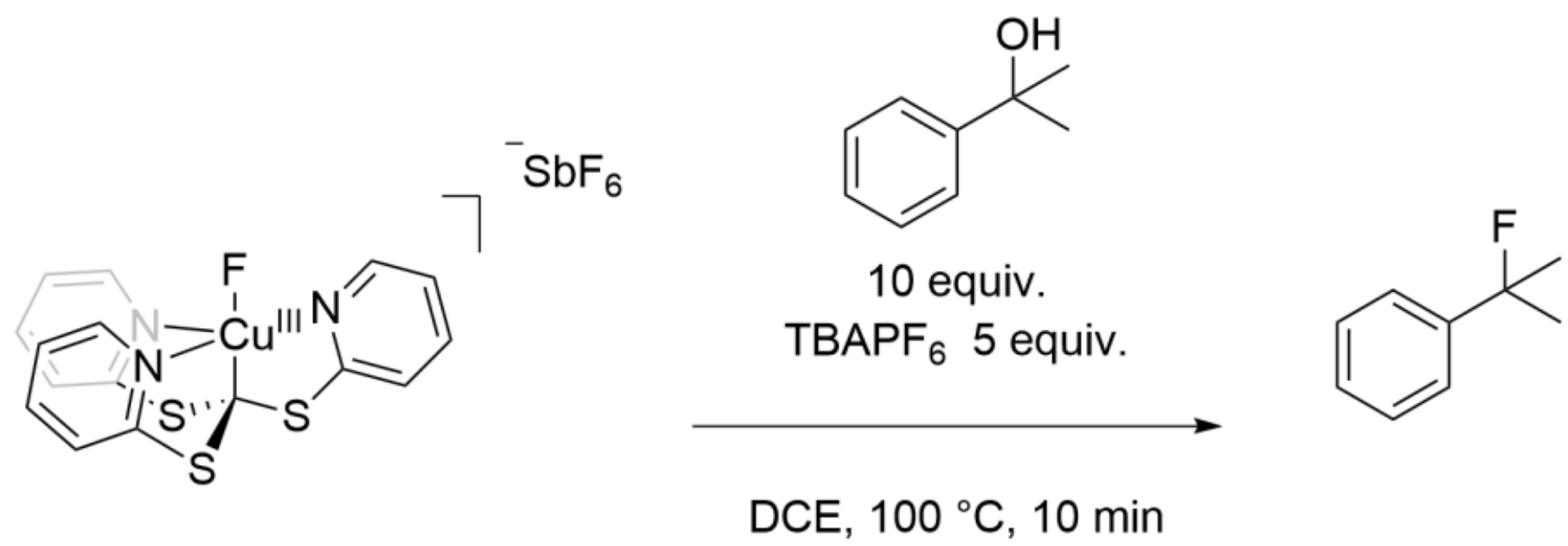

Entry	Deviation from standard conditions	Yield%
**1**	**None**	**99**
2	1 equiv. of $TBAPF_6$	56
3	2 equiv. of $TBAPF_6$	67
4	5 equiv. of $LiPF_6$	35
5	5 equiv. of $TBABF_4$	17
6	5 equiv. of CsF	0

Standard conditions of deoxyhalogenation

Deoxyfluorination

To a 10 mL screw cap test tube equipped with a stir bar, [**L**Cu^{III}F][SbF_6] (16.5 mg, 0.0250 mmol, 1.00 equiv.), substrate (0.250 mmol, 10.0 equiv.), and $TBAPF_6$ (48.0 mg, 0.125 mmol, 5.00 equiv.) were added. The solids were dissolved in dichloroethane (2 mL). The reaction was stirred at 100 °C for 5 min – 2 hrs. The reactions were stopped when the deep red color of the [**L**Cu^{III}F]$^+$ disappeared. Yields were determined using ^{19}F NMR with trifluorotoluene as the internal standard.

Deoxychlorination

To a 10 mL screw cap test tube equipped with a stir bar, [**L**Cu^{III}Cl][SbF_6] (16.9 mg, 0.025 mmol, 1.0 equiv.) and substrate (0.250 mmol, 10.0 equiv.) were added. The solids were dissolved in dichloroethane (2 mL). The reaction was stirred at 100 °C for 10 min – 2 hrs. The reactions were stopped when the deep violet color of the [**L**Cu^{III}Cl]$^+$ disappeared. Yields were determined using ^{1}H NMR with trimethoxy benzene as internal standard.

Deoxybromination

To a 10 mL screw cap test tube equipped with a stir bar, [**L**Cu^{III}Br][SbF_6] (18 mg, 0.025 mmol, 1.0 equiv.) and substrate (0.250 mmol, 10.0 equiv.) were added. The solids were dissolved in dichloroethane (2 mL). The reaction was stirred at 100 °C for 10 min – 2 hrs. The reactions were stopped when the deep violet color of the [**L**Cu^{III}Br]$^+$ disappeared. Yields were determined using ^{1}H NMR with trimethoxy benzene as internal standard.

Alkene formation

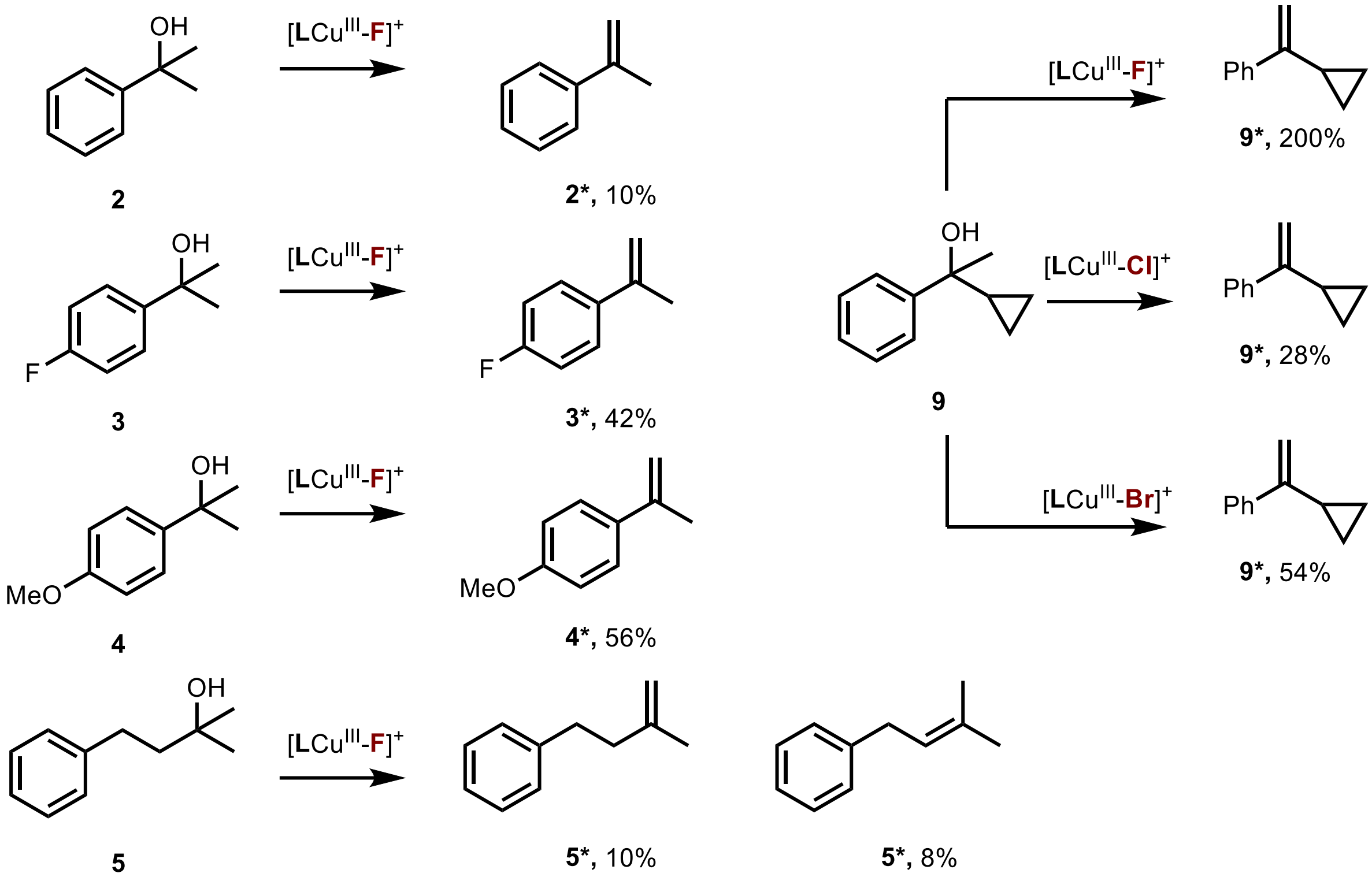

Table S3. Yield of alkene decomposition products under the standard reaction conditions.

Deoxyhalogenation of adamantanol with [LCu^{III}X]$^+$

To a 10 mL screw cap test tube equipped with a stir bar, [**L**Cu^{III}X][SbF$_6$] (0.025 mmol, 1.00 equiv.) and adamantanol (38.1 mg, 0.250 mmol, 10.0 equiv.) were added. The solids were dissolved in dichloroethane (2 mL). The reaction was stirred at 100 °C for 2 - 3 hrs. The reactions were stopped when the deep red/violet color of the [**L**Cu^{III}X]$^+$ disappeared. Yields were determined using ^{19}F NMR with trifluorotoluene as the internal standard or by GC-MS.

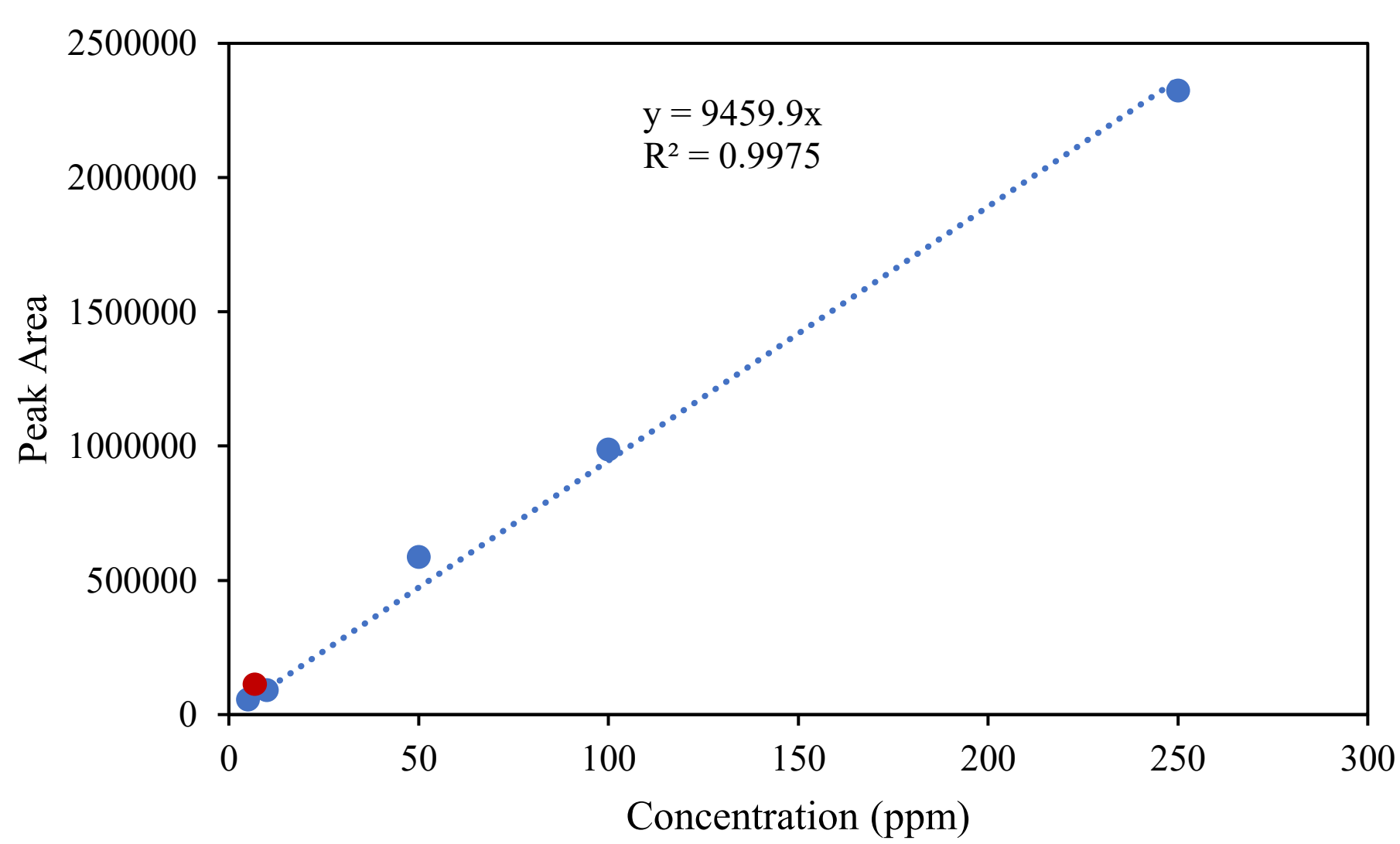

Figure S21. GC-MS calibration curve of 1-chloroadamantane standard. Analysis of the reaction mixture of [**L**$Cu^{III}Cl]^+$ with adamantanol shows a peak of 1-chloroadamantane with an area of 56596.78 (indicated by the red dot). This concentration suggests the yield of 1-chloroadamantane is ca. 31%.

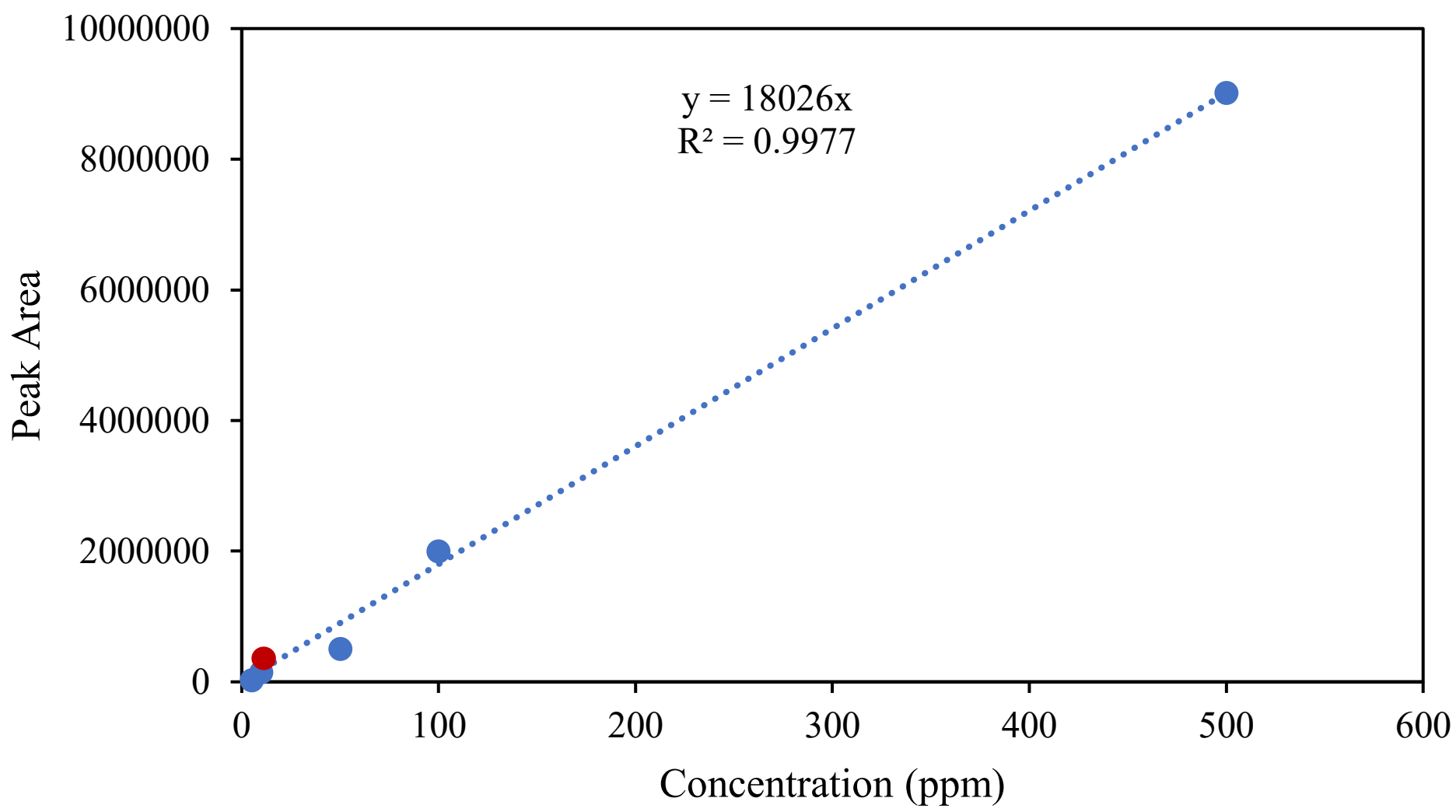

Figure S22. GC-MS calibration curve of 1-bromoadamantane standard. Analysis of the reaction mixture of [**L**$Cu^{III}Br]^+$ with adamantanol shows a peak of 1-bromoadamantane with an area of 59496.56 (indicated by the red dot). This concentration suggests the yield of 1-bromoadamantane is ca. 57%.

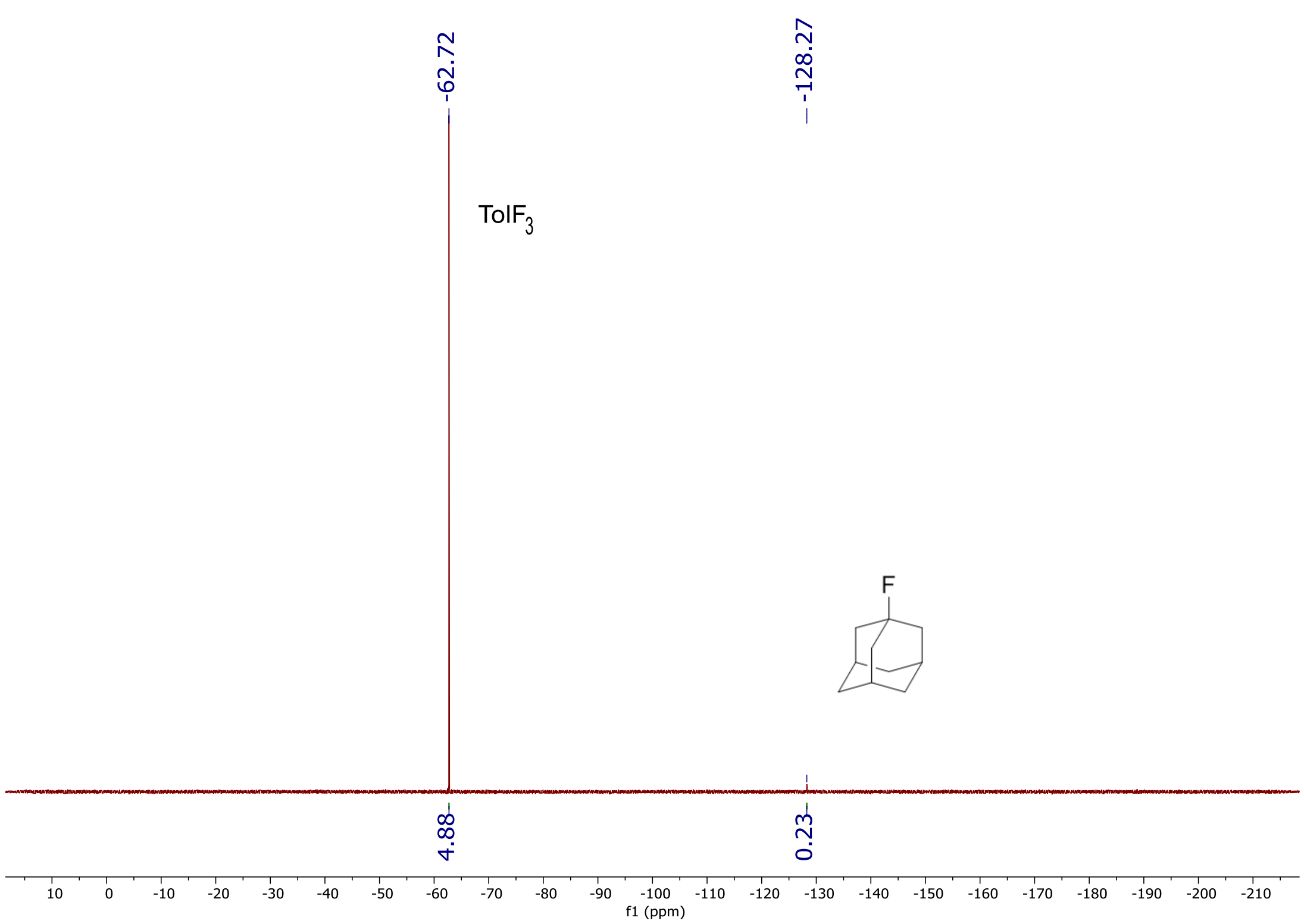

Figure S23. $^{19}F\{^1H\}$ NMR spectrum (376.5 MHz, CD_3Cl) of the standard deoxyfluorination reaction with adamantanol, showing the formation of the monofluorinated product at –128.27 ppm.[8]

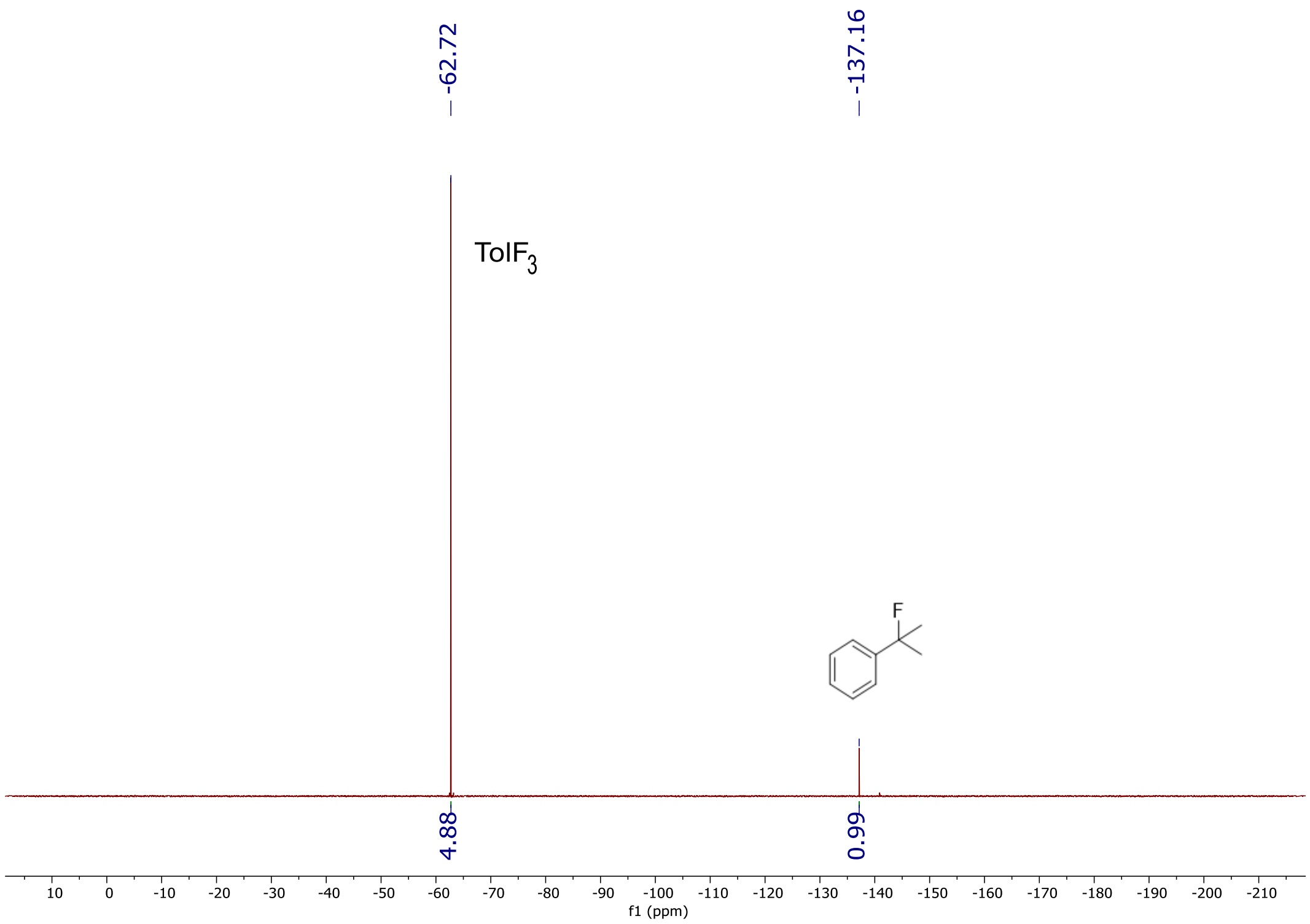

Figure S24. $^{19}F\{^1H\}$ NMR spectrum (376.5 MHz, CD_3Cl) of the standard deoxyfluorination reaction with cumyl alcohol, showing the formation of the monofluorinated product at –137.16 ppm.[9]

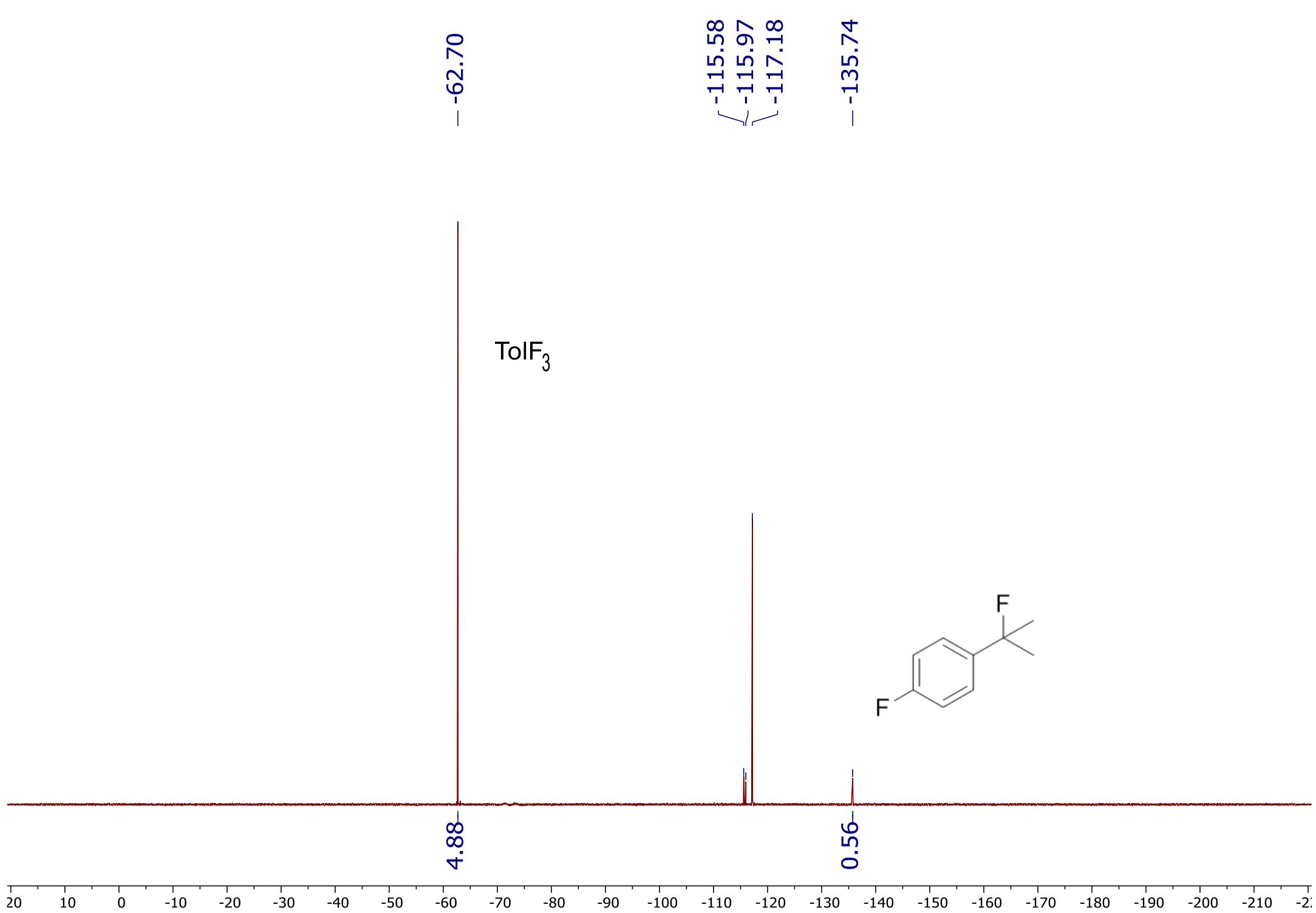

Figure S25. $^{19}F\{^1H\}$ NMR spectrum (376.5 MHz, CD_3Cl) of the standard deoxyfluorination reaction with 4-fluorocumyl alcohol, showing the formation of the monofluorinated product at –135.74 ppm. Product was independently synthesized to confirm with NMR.

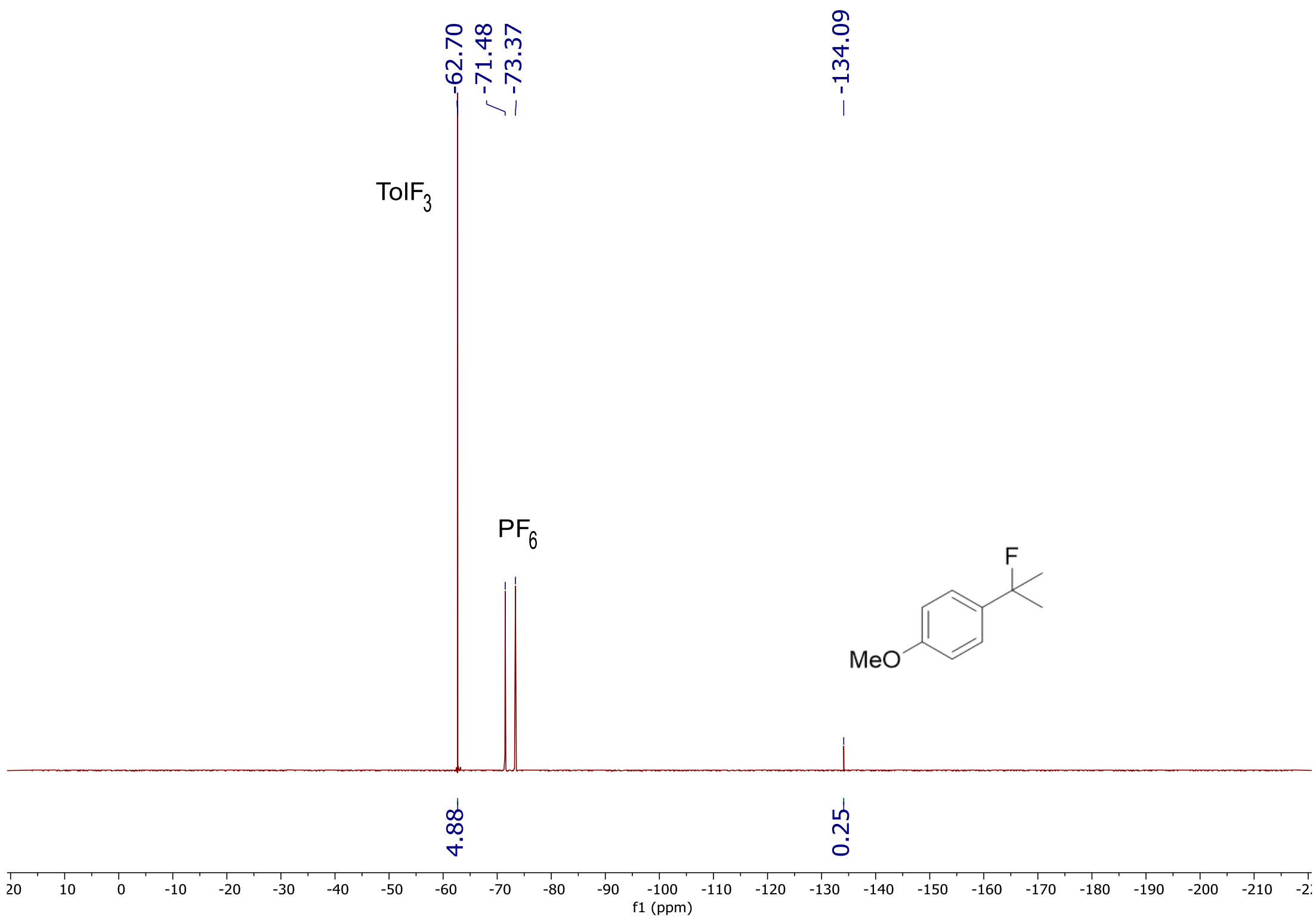

Figure S26. $^{19}F\{^1H\}$ NMR spectrum (376.5 MHz, CD_3Cl) of the standard deoxyfluorination reaction with 2-(4-methoxyphenyl)propan-2-ol, showing the formation of the monofluorinated product at –134.09 ppm.[10]

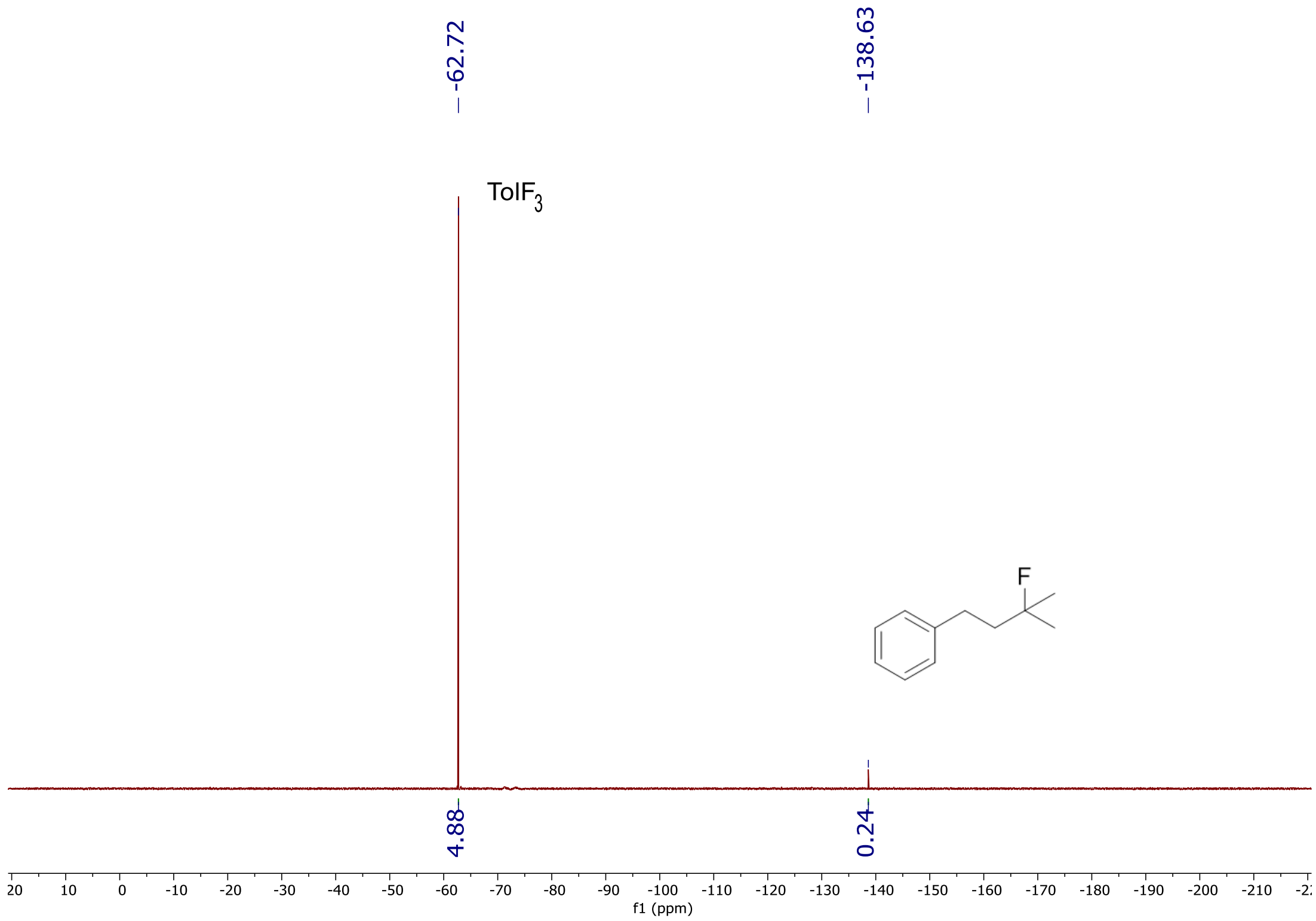

Figure S27. $^{19}F\{^{1}H\}$ NMR spectrum (376.5 MHz, CD_3Cl) of the standard deoxyfluorination reaction with 4-phenyl-2-methyl-2-butanol, showing the formation of the monofluorinated product at –138.63 ppm.[11]

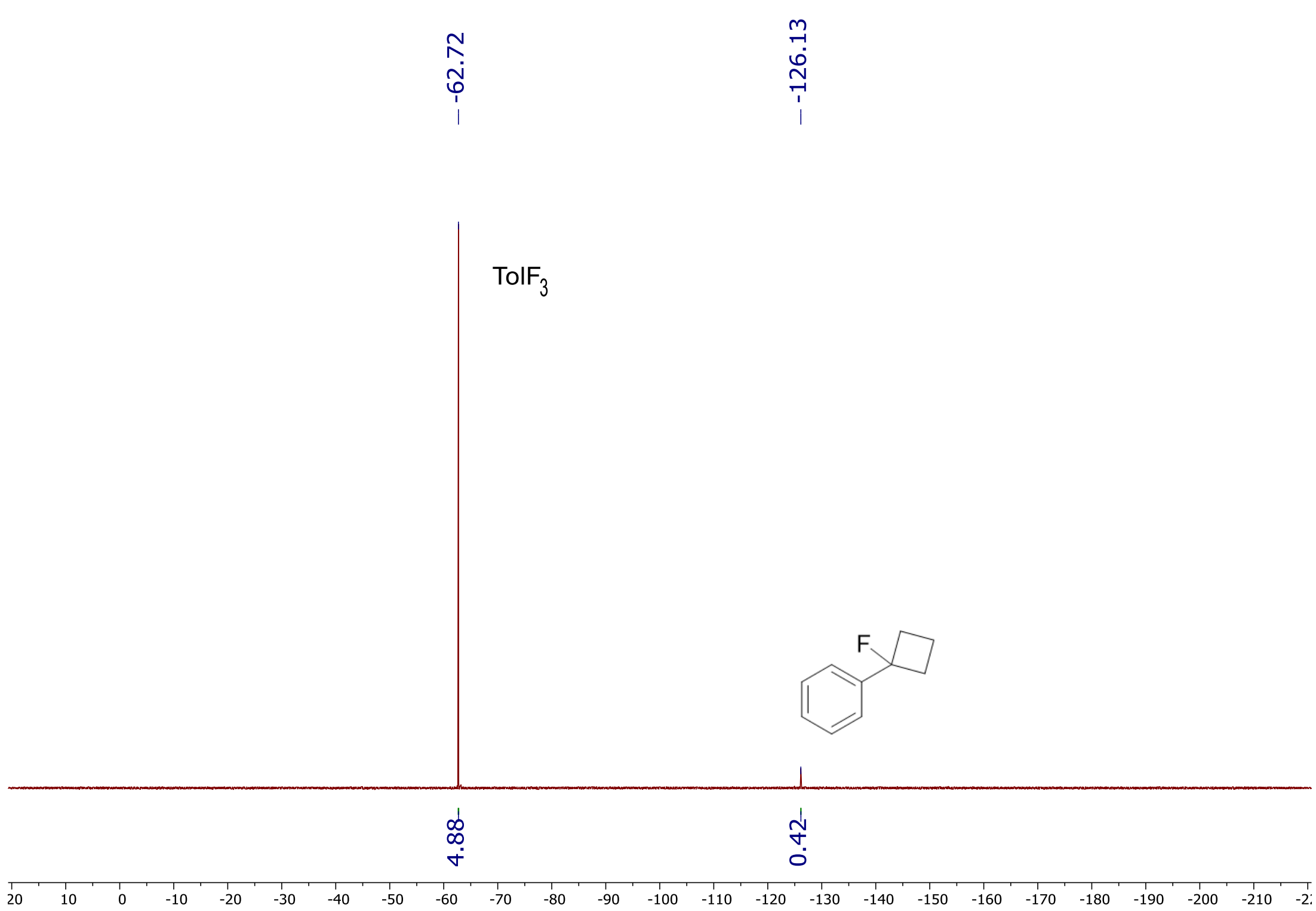

Figure S28. $^{19}F\{^{1}H\}$ NMR spectrum (376.5 MHz, CD_3Cl) of the standard deoxyfluorination reaction with 1-phenylcyclobutanol, showing the formation of the monofluorinated product at –126.13 ppm.[12]

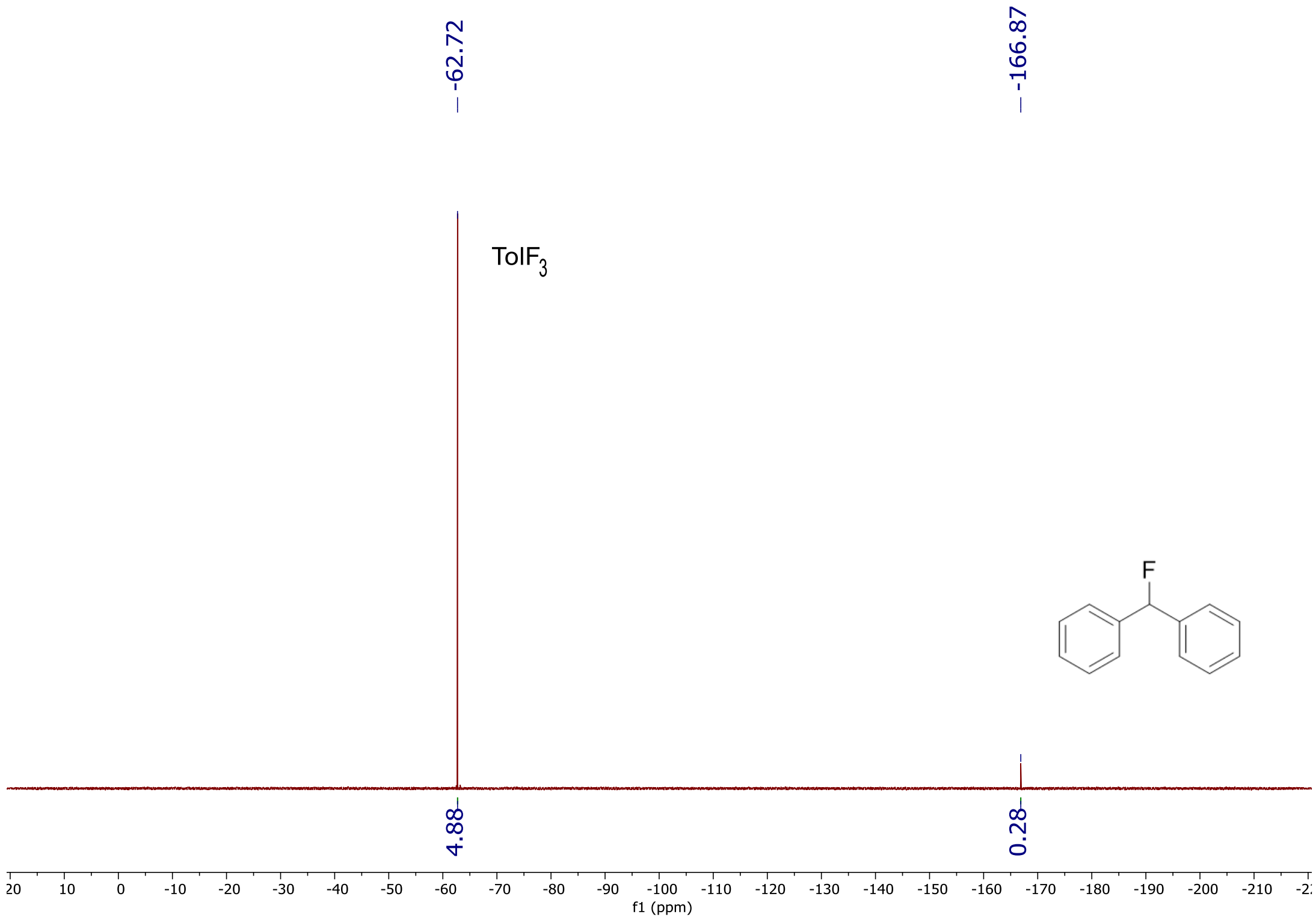

Figure S29. $^{19}F\{^{1}H\}$ NMR spectrum (376.5 MHz, CD_3Cl) of the standard deoxyfluorination reaction with 1,1-Diphenylmethanol, showing the formation of the monofluorinated product at –166.87 ppm.[9]

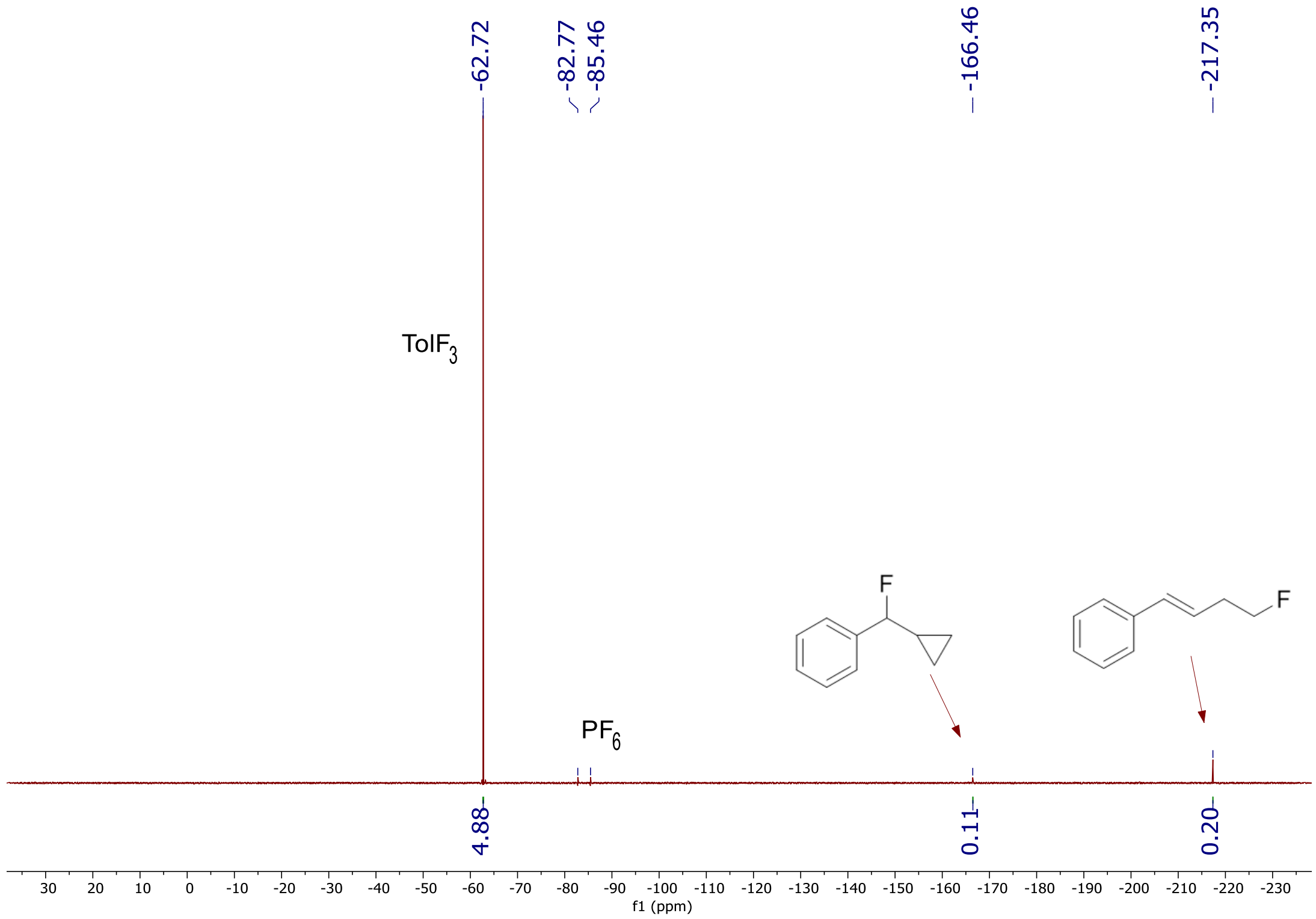

Figure S30. $^{19}F\{^{1}H\}$ NMR spectrum (376.5 MHz, CD_3Cl) of the standard deoxyfluorination reaction with 1-phenyl-1-cyclopropylmethanol, showing the formation of the monofluorinated product at –166.46 ppm and the ring opened product at –217.35 ppm.[13,14]

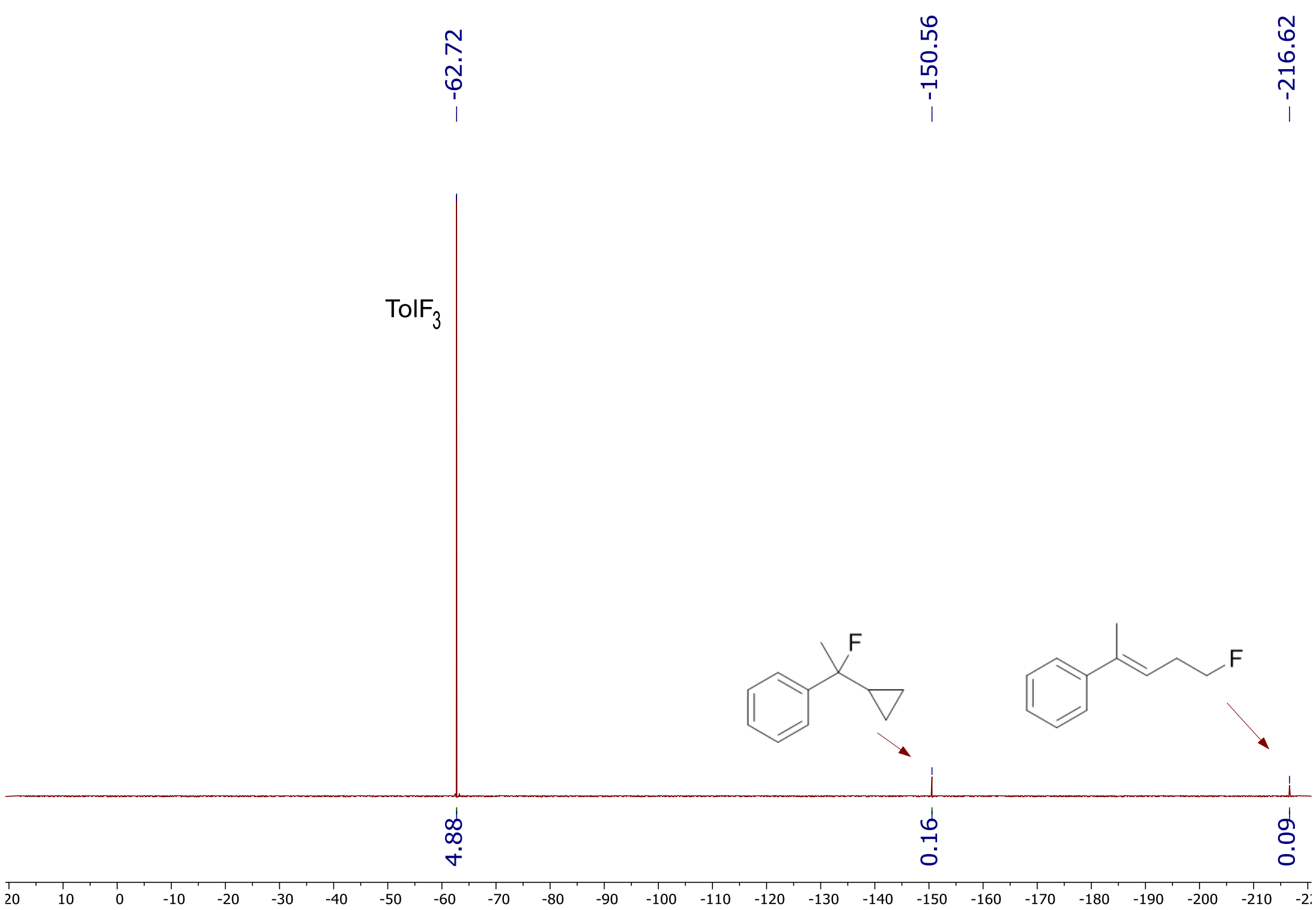

Figure S31. $^{19}F\{^1H\}$ NMR spectrum (376.5 MHz, CD_3Cl) of the standard deoxyfluorination reaction with 1-cyclopropyl-1-phenylethanol, showing the formation of the monofluorinated product at –150.56 ppm and the ring opened product at –216.62 ppm.[14]

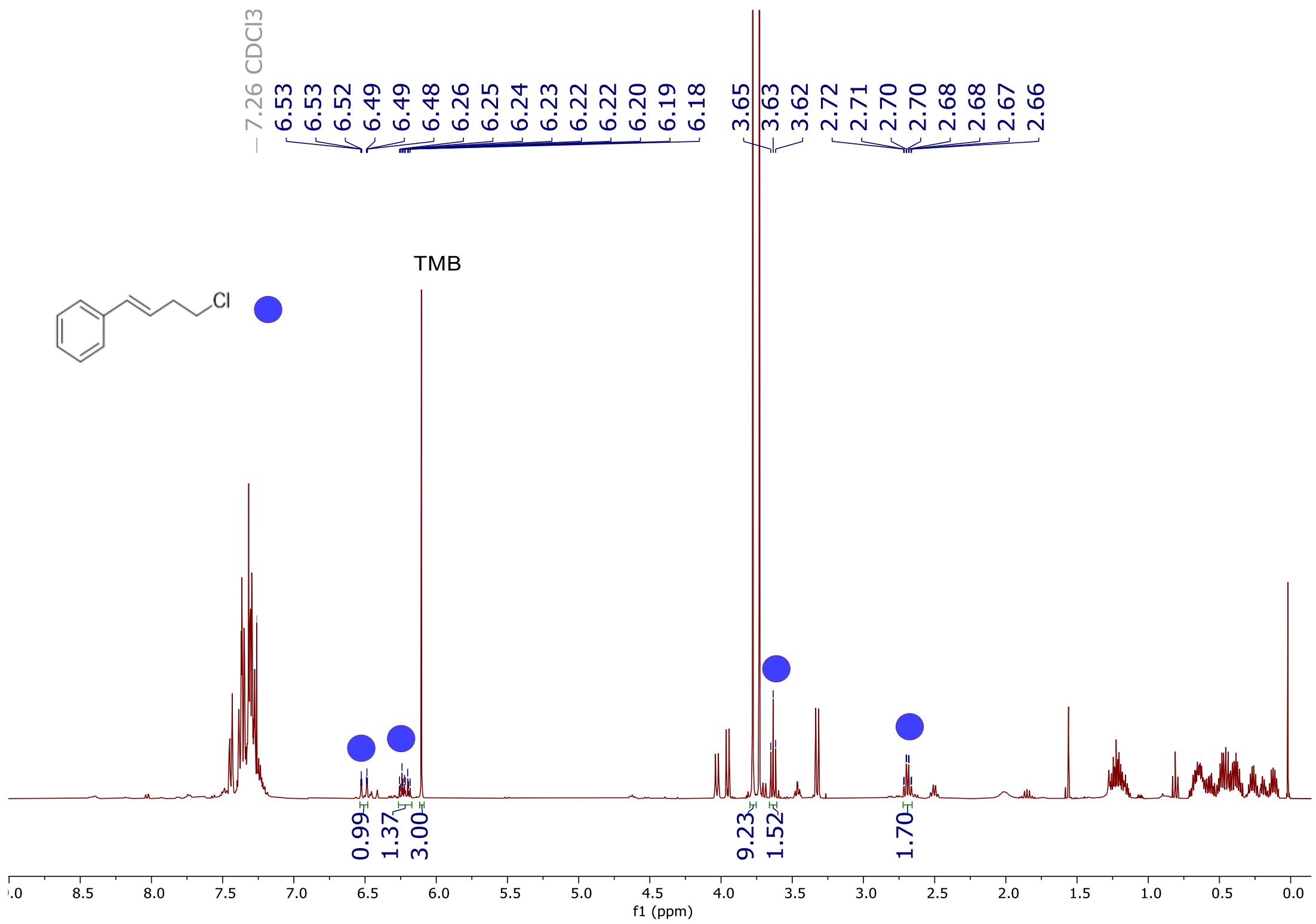

Figure S32. ^1H NMR spectrum (400 MHz, CD_3Cl) of the standard deoxychlorination reaction with 1-phenyl-1-cyclopropylmethanol, showing the formation of and the ring opened product.[15]

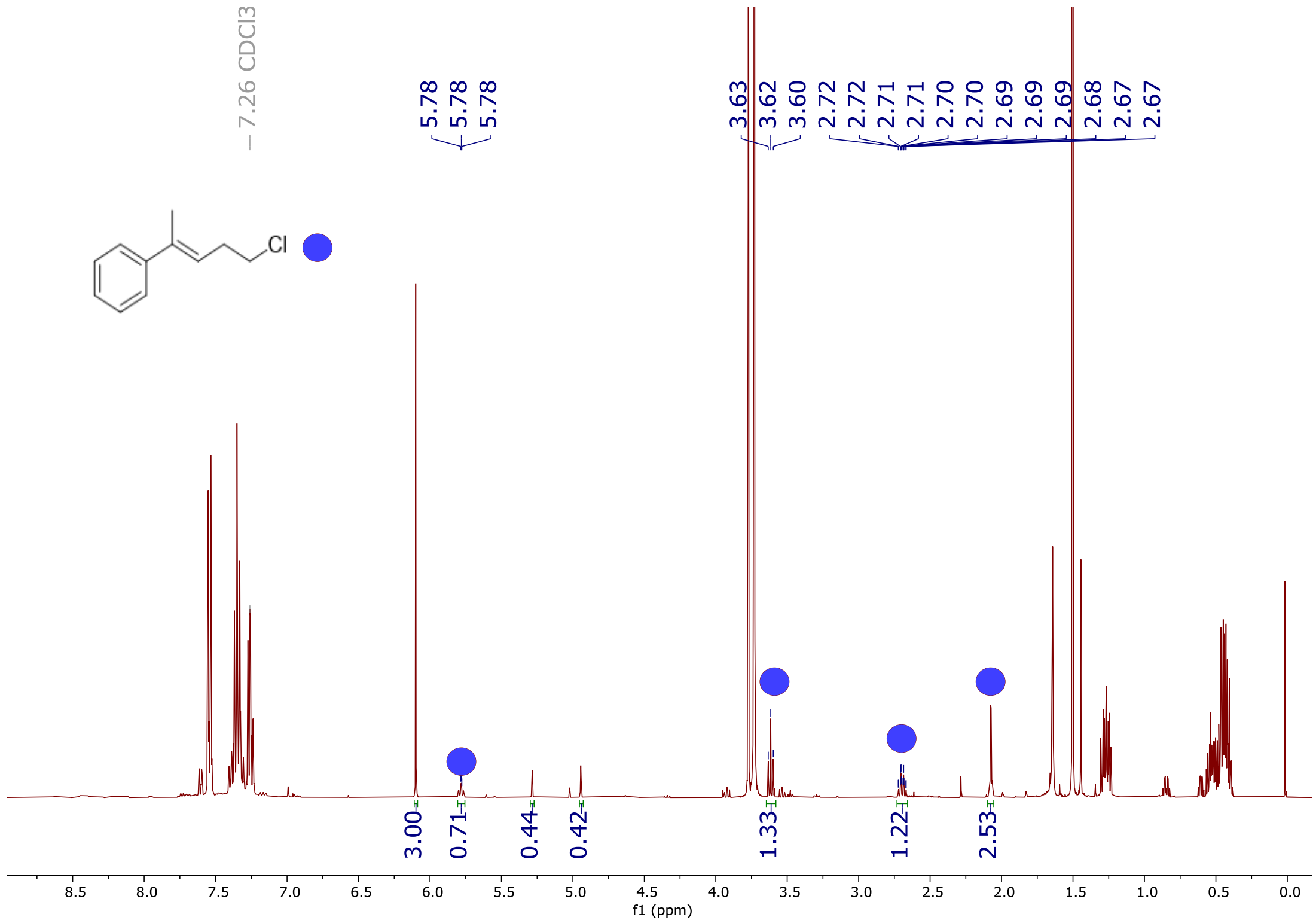

Figure S33. ^{1}H NMR spectrum (400 MHz, CD_3Cl) of the standard deoxychlorination reaction with 1-cyclopropyl-1-phenylethanol, showing the formation of the and the ring opened product.[16]

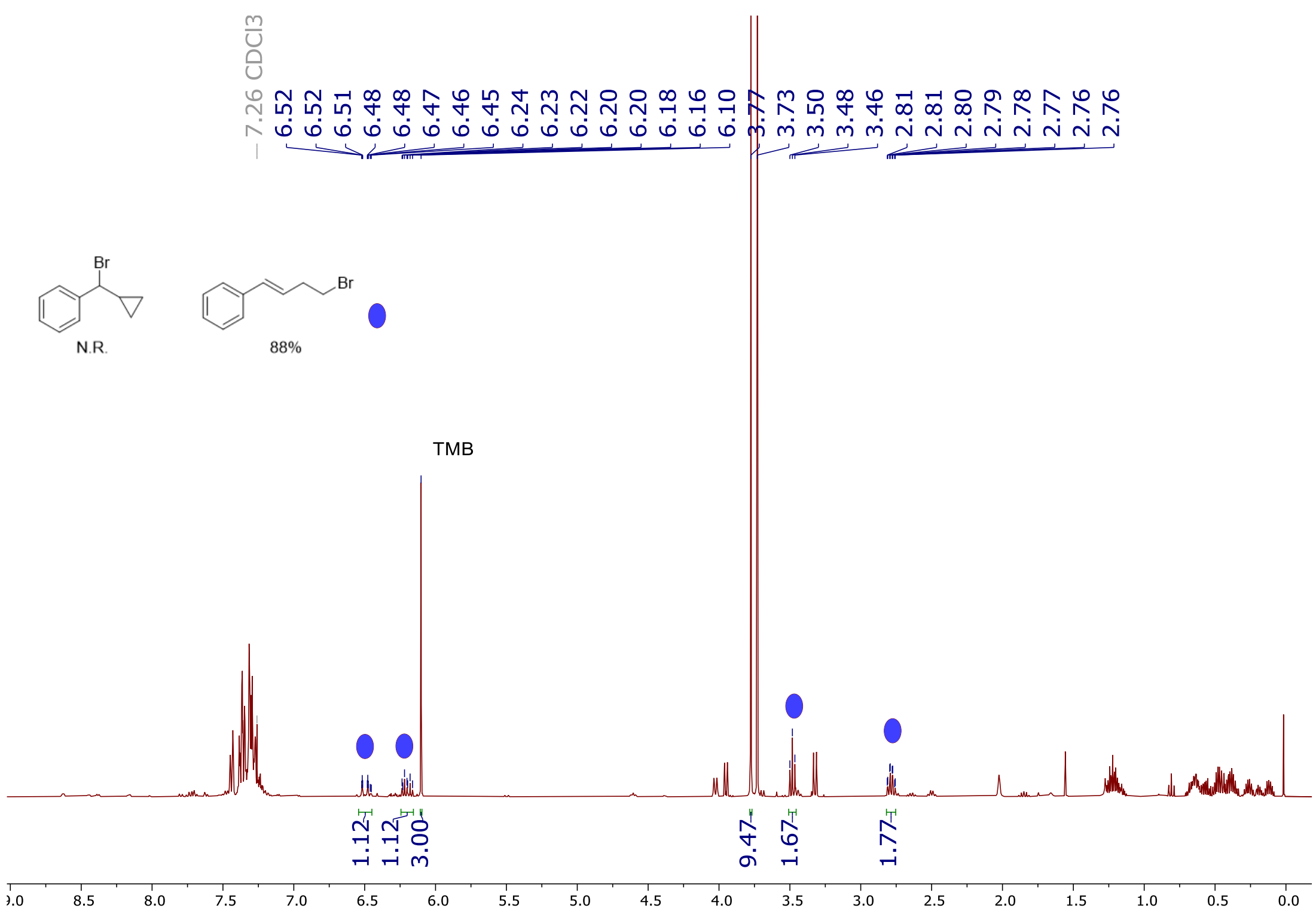

Figure S34. ^{1}H NMR spectrum (400 MHz, CD_3Cl) of the standard deoxybromination reaction with 1-phenyl-1-cyclopropylmethanol, showing the formation of and the ring opened product.[17]

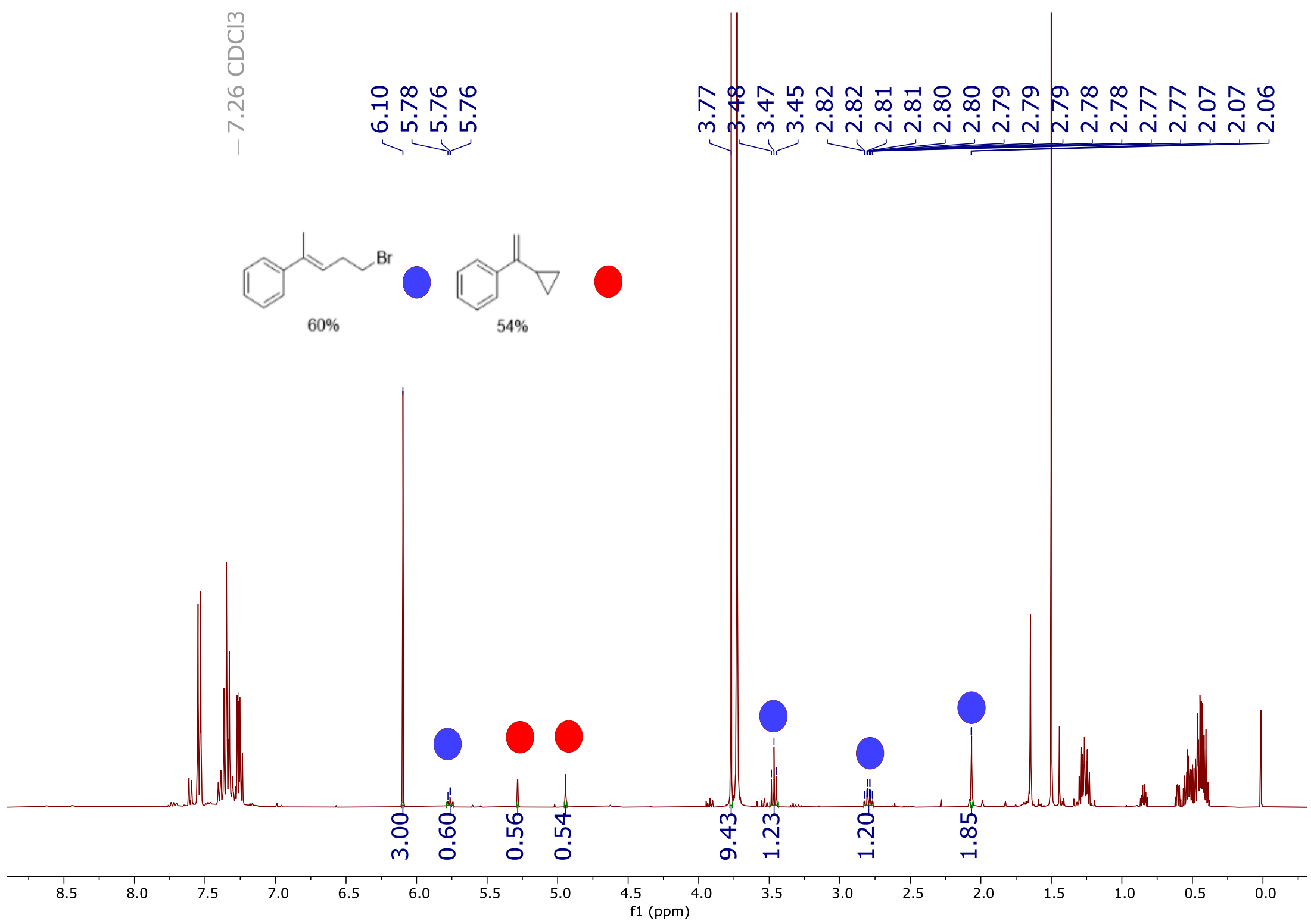

Figure S35. ^{1}H NMR spectrum (400 MHz, CD_3Cl) of the standard deoxybromination reaction with 1-cyclopropyl-1-phenylethanol, showing the formation of the and the ring opened product.[18]

EPR spin quantization of $[LCu^{II}MeCN]^+$ produced from deoxyfluorination reactions

Spectra were recorded on a Magnettech ESR5000 benchtop EPR (continuous-wave, X-band) spectrometer at 77K. For all samples, a modulation frequency of 100 kHz and a time constant of 60s were employed. EPR spin quantization were performed using Igor64.

To a 10 mL screw cap test tube equipped with a stir bar, $[\mathbf{L}Cu^{III}F][SbF_6]$ (16.5 mg, 0.0250 mmol, 1.00 equiv.) and cumyl alcohol (34.0 mg, 0.250 mmol, 10.0 equiv.) were added. The solids were dissolved in dichloroethane (1.95 mL) and MeCN (50 µL). The reaction was stirred at 100 °C for 25 minutes. The reactions were stopped when the deep red color of the $[\mathbf{L}Cu^{III}F]^+$ disappeared. An aliquot of the reaction was taken for ^{19}F NMR analysis with trifluorotoluene as internal standard. Under a nitrogen atmosphere, the crude reaction mixture was dried down and then dissolved in a 1:1 toluene : DCM solution to give a theoretical [Cu] of 1.0 mM. Then, 0.2 mL of the solution was transferred to a 4 mm quartz EPR tube (Wilmad Glass Company) prior to freezing in liquid nitrogen for analysis. A 1.0 mM $Cu^{II}OTf_2$ solution in MeCN was used as a spin quantification standard. The yield of $[\mathbf{L}Cu^{II}MeCN]^+$ was calculated to be 34% based on double integration of the EPR signals against an external standard of $Cu^{II}OTf_2$ solution in MeCN measured under identical conditions.

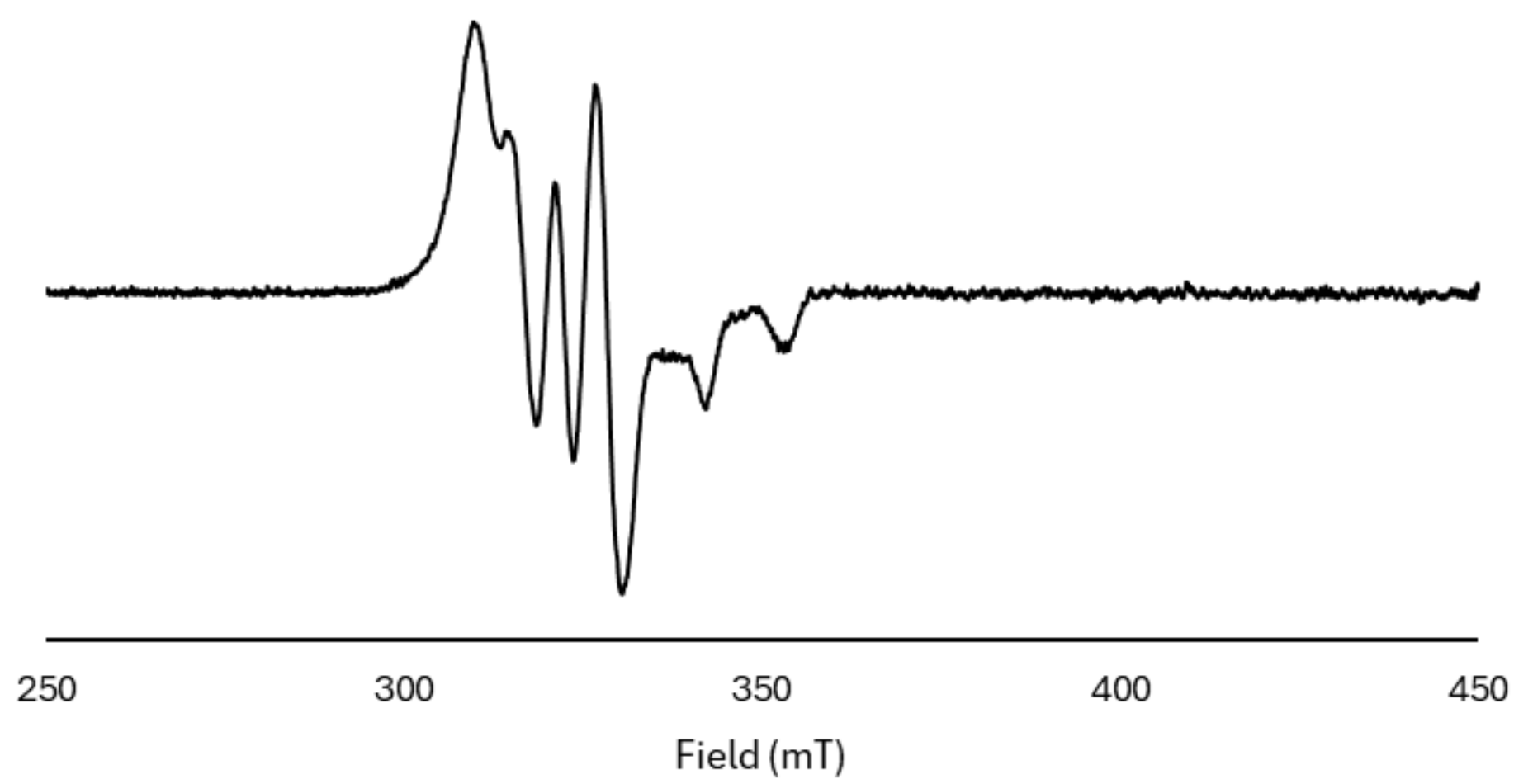

Figure S36. EPR spectrum of [**L**Cu^{II}MeCN]$^+$ in solution after deoxyfluorination reaction of [**L**Cu^{III}F][SbF_6] + cumyl alcohol. The features of the EPR signal match that reported in the literature.[19]

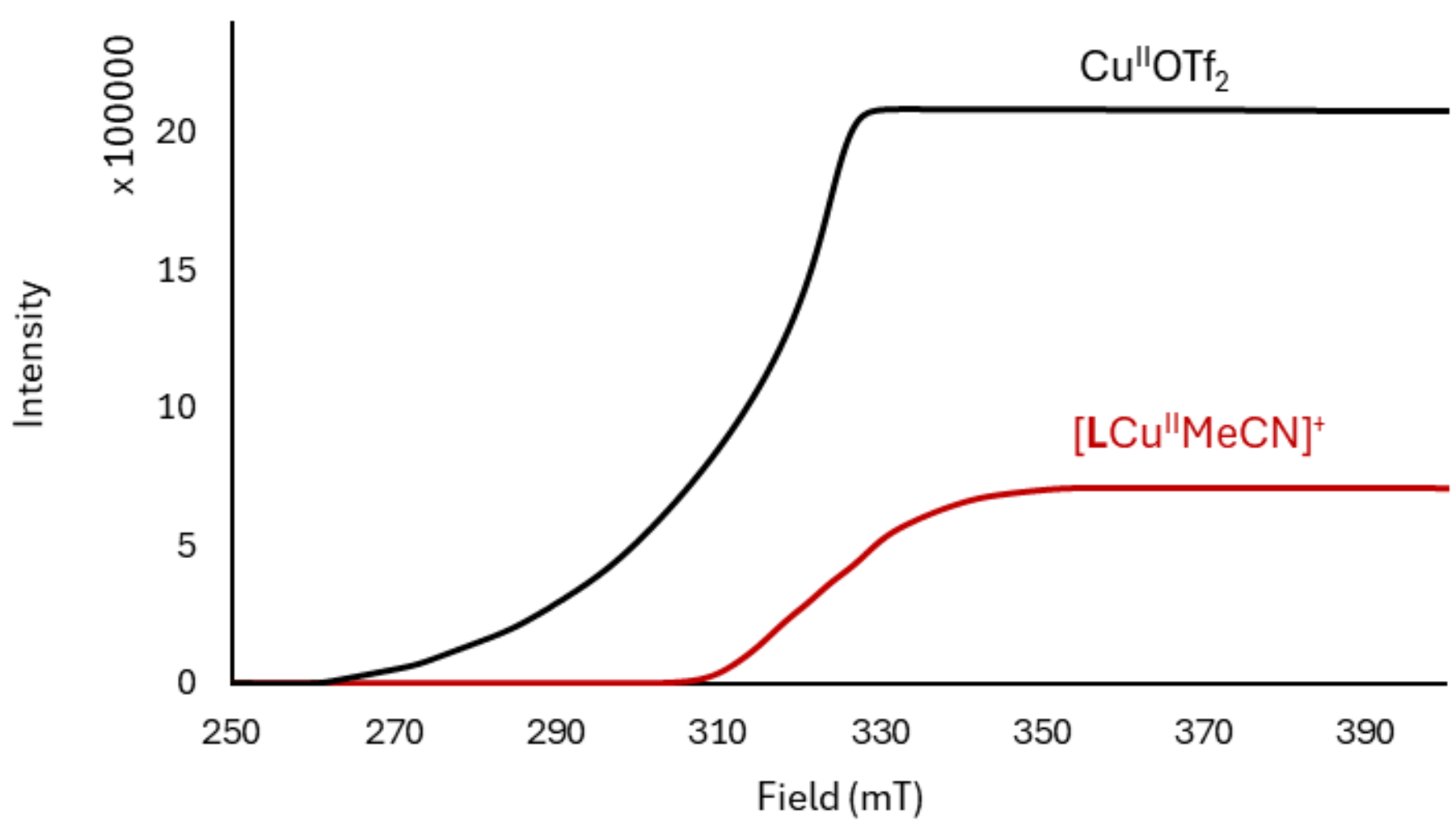

Figure S37. EPR spin quantification of [**L**Cu^{II}MeCN]$^+$ in solution after deoxyfluorination by [**L**Cu^{III}F][SbF_6]. Yield of [**L**Cu^{II}MeCN]$^+$ is 34%

EPR Spin Quantization of [LCu^{II}MeCN]$^+$ produced from the decomposition of [LCu^{III}OH]$^+$

Stock solutions of [**L**Cu^{II}OH•TEAH][PF_6] (3.0 mM in DCM) and NAr_3[SbF_6] (3.0 mM in DCM) were made. To a 20 mL scintillation vial, [**L**Cu^{II}OH•TEAH][PF_6] (1.0 mL, 3.0 mM, 1.0 equiv.) solution was added. Then NAr_3[SbF_6] (1.0 mL, 3.0 mM, 1.0 equiv.) was added to the vial to generate a putative [**L**Cu^{III}OH]$^+$ species in situ. After allowing the reaction to stir for 10 min, MeCN (200 uL) was added to the reaction mixture. The reaction was allowed to stir for 5 minutes. The solution was then diluted with toluene (800 uL) to give a theoretical [Cu] of 1.0 mM. Then, 0.2 mL of the solution was transferred to a 4 mm quartz EPR tube (Wilmad Glass Company) prior to freezing in liquid nitrogen for analysis. A 1.0 mM $Cu^{II}OTf_2$ solution in MeCN was used as a spin quantification standard. The yield of [**L**Cu^{II}MeCN]$^+$ was

calculated to be 46% based on double integration of the EPR signals against an external standard of $Cu^{II}OTf_2$ solution in MeCN measured under identical conditions.

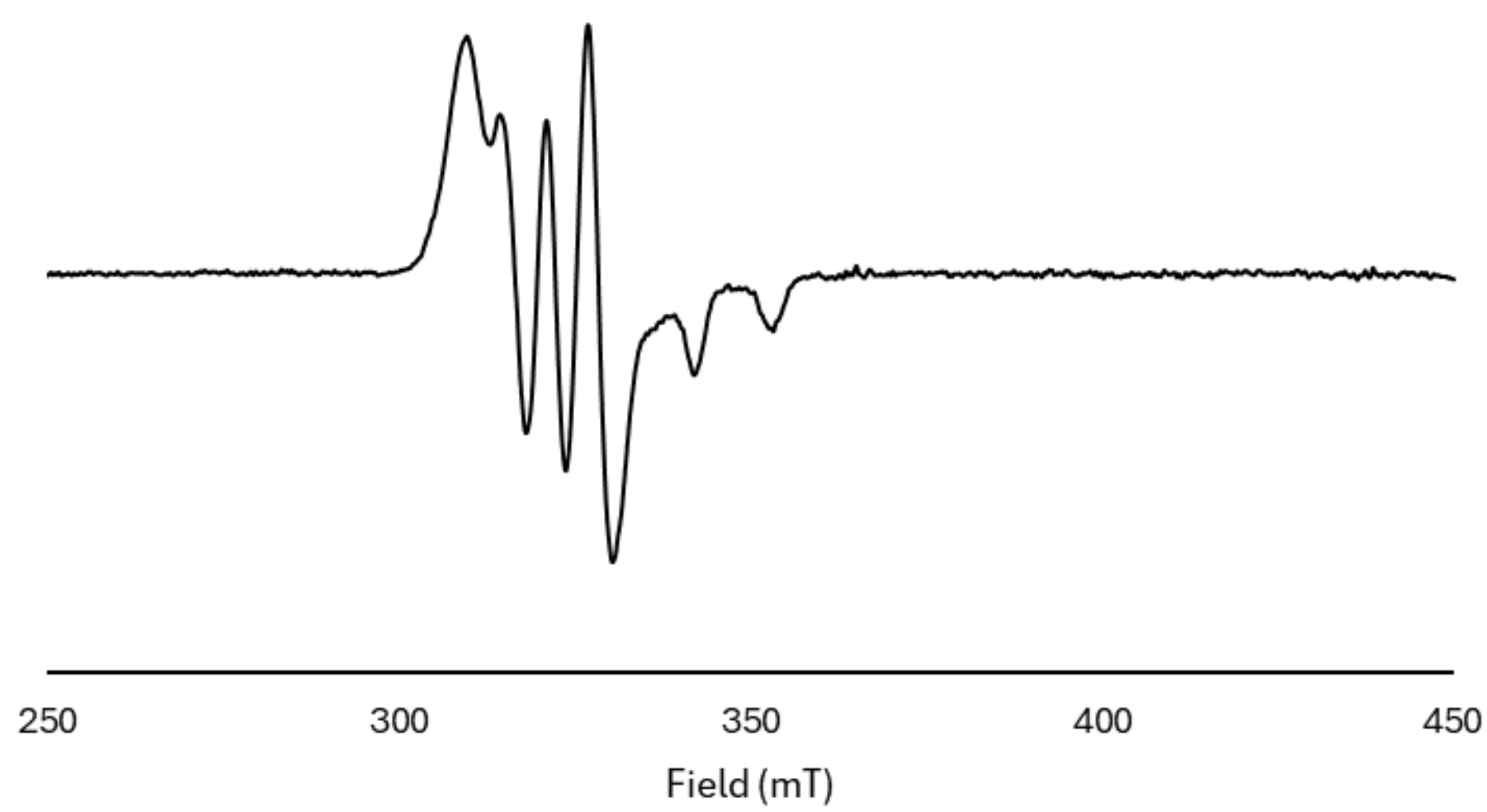

Figure S38. EPR spectrum of [**L**Cu^{II}MeCN]$^+$ in solution after decomposition of [**L**Cu^{III}OH]$^+$ at room temp. The features of the EPR signal match that those reported in the literature.[19]

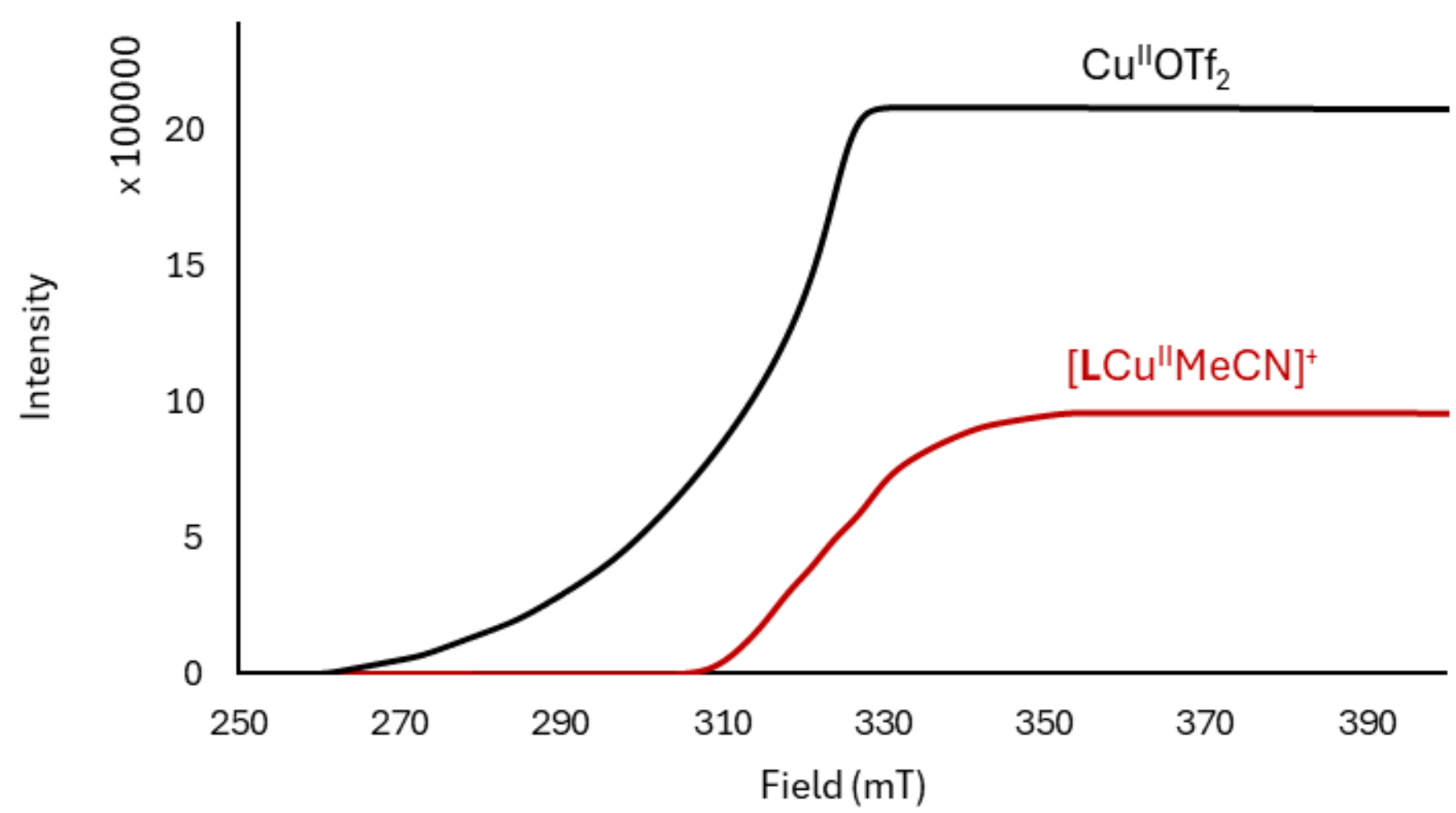

Figure S39. EPR spin quantification of [**L**Cu^{II}MeCN]$^+$ in solution after decomposition of [**L**Cu^{III}OH]$^+$ at room temp. Yield of [**L**Cu^{II}MeCN]$^+$ is 46%

Computational details

DFT calculations were performed in ORCA[20,21] Geometry optimizations were performed at the B3LYP/6-31G(d) (SDD for Cu, Br) level of theory with D3BJ dispersion correction. A frequency calculation was then performed on the optimized structures at the same level of theory, confirming the absence of imaginary frequencies. A single point energy calculation on the optimized structure was then performed at B3LYP/def2-TZVP (SDD for Cu, Br) level of theory, with CPCM solvent modeling (dichloromethane) and D3BJ dispersion correction.

Cartesian coordinates of complexes used in computations

Complex – Level of theory optimized

Charge, spin multiplicity

TPTM$Cu^{III}F^{+}$ S = 0 – B3LYP/6-31G(d) D3BJ (SDD for Cu)

1, 1

29	4.711248000	4.912902000	11.836910000
16	6.202746000	6.180078000	14.179201000
16	3.371970000	6.966248000	13.655566000
16	5.534220000	7.852089000	11.799721000
7	4.198016000	6.121495000	10.312973000
6	4.530004000	7.423279000	10.401276000
7	6.651506000	4.509142000	12.179733000
7	3.323516000	4.364661000	13.185826000
6	7.263413000	5.162902000	13.185112000
6	7.349101000	3.668844000	11.398937000
6	1.511343000	3.689828000	15.157351000
6	3.486293000	5.676925000	9.265050000
6	8.621683000	5.005022000	13.451053000
6	4.152245000	8.349893000	9.432159000
6	1.923050000	5.015622000	15.018492000
6	2.937188000	3.086528000	13.325519000
6	3.084767000	6.535001000	8.247793000
6	4.962652000	6.526917000	12.900253000
6	2.831605000	5.312919000	14.005228000

6	2.021657000	2.710744000	14.301675000
6	3.422868000	7.887409000	8.336405000
6	9.345546000	4.123036000	12.648157000
6	8.704684000	3.444124000	11.609128000
1	3.114817000	8.582726000	7.561605000
1	4.411725000	9.397250000	9.538282000
1	2.524490000	6.149230000	7.403646000
1	3.284883000	4.611131000	9.269974000
1	3.370043000	2.398321000	12.607536000
1	1.712919000	1.674811000	14.384343000
1	0.801992000	3.424282000	15.935158000
1	1.559807000	5.793548000	15.680636000
1	9.096170000	5.561124000	14.251777000
1	10.405521000	3.974598000	12.830077000
1	9.242859000	2.748532000	10.975225000
1	6.767538000	3.172488000	10.629578000
9	4.477632000	3.414612000	10.849760000

TPTMCu^{III}F^{+} S = 1 – B3LYP/6-31G(d) D3BJ (SDD for Cu)

1, 3

29	4.677692000	4.761448000	11.735514000
16	6.249030000	6.227761000	14.179110000
16	3.372751000	6.973823000	13.725114000
16	5.489748000	7.912253000	11.808831000
7	4.183385000	6.110025000	10.290158000
6	4.488673000	7.408779000	10.407747000
7	6.655710000	4.491726000	12.159821000
7	3.315714000	4.334236000	13.189093000
6	7.262675000	5.173814000	13.139587000
6	7.366822000	3.624933000	11.417435000
6	1.509614000	3.693037000	15.188973000

6	3.510875000	5.683049000	9.206649000
6	8.620467000	5.034394000	13.421973000
6	4.125313000	8.354952000	9.450869000
6	1.958800000	5.005991000	15.047814000
6	2.884373000	3.068003000	13.326081000
6	3.117817000	6.556685000	8.199219000
6	4.995762000	6.771735000	13.061820000
6	2.861760000	5.279798000	14.022014000
6	1.976837000	2.708215000	14.315954000
6	3.429667000	7.912106000	8.326030000
6	9.357855000	4.133801000	12.653418000
6	8.723840000	3.416577000	11.636550000
1	3.130366000	8.621368000	7.560524000
1	4.376087000	9.401358000	9.587454000
1	2.580846000	6.181787000	7.335102000
1	3.323989000	4.614662000	9.176399000
1	3.280894000	2.368475000	12.597580000
1	1.643727000	1.679721000	14.398541000
1	0.806155000	3.443259000	15.977255000
1	1.624952000	5.794672000	15.713410000
1	9.081541000	5.615523000	14.213298000
1	10.417989000	3.999650000	12.845138000
1	9.269559000	2.707164000	11.024569000
1	6.796615000	3.095475000	10.661198000
9	4.443815000	3.268006000	10.750870000

Other NMR spectra

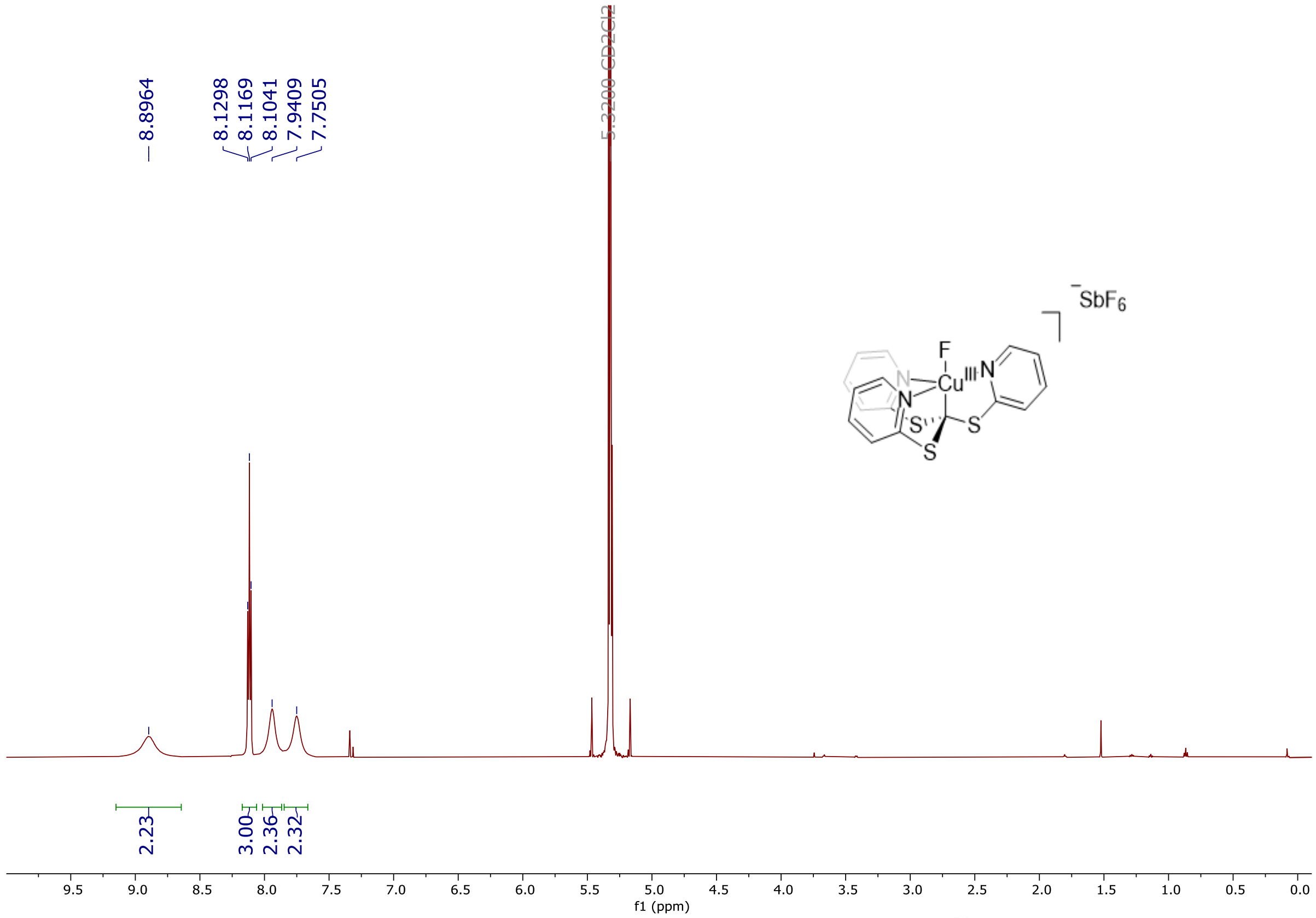

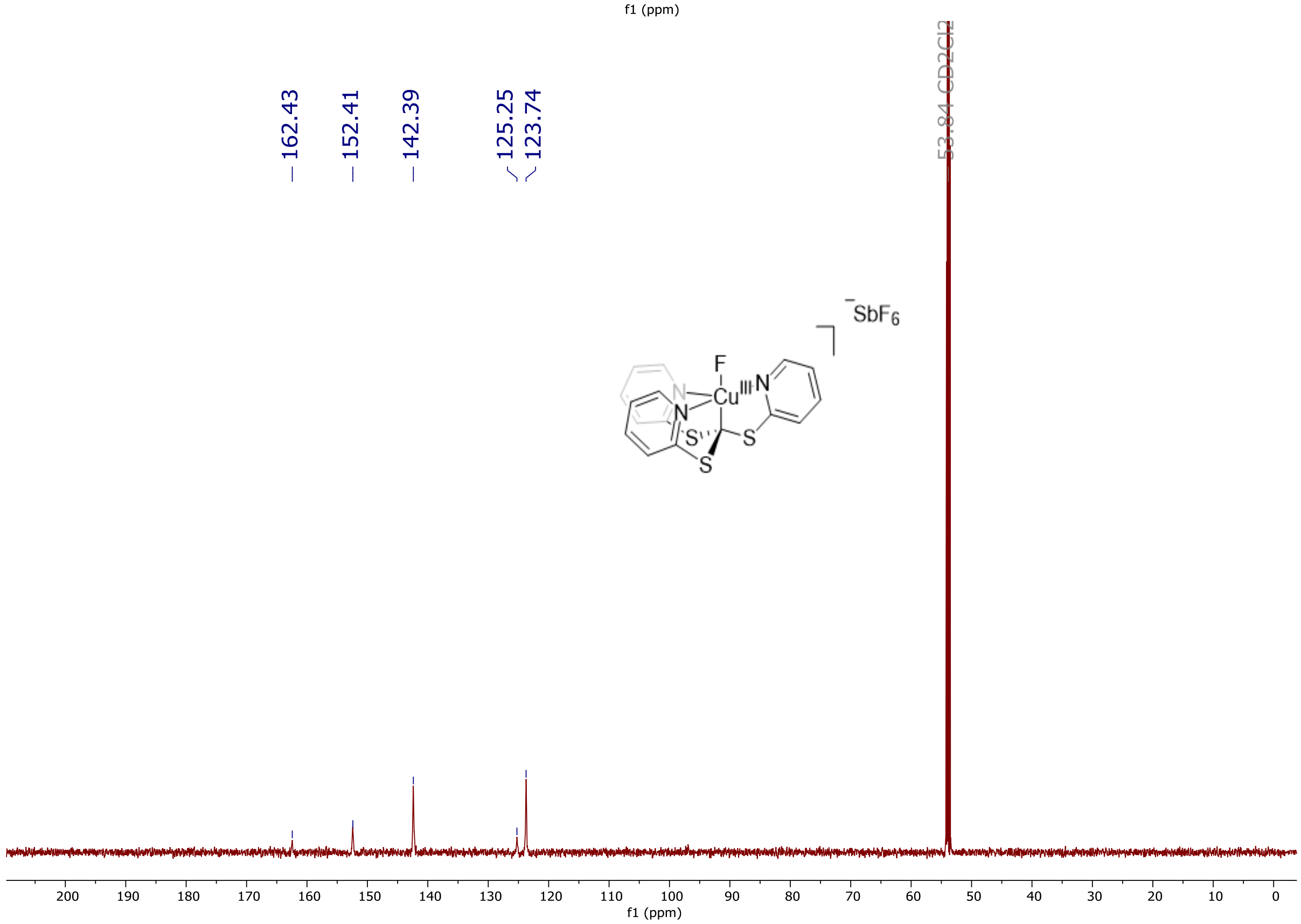

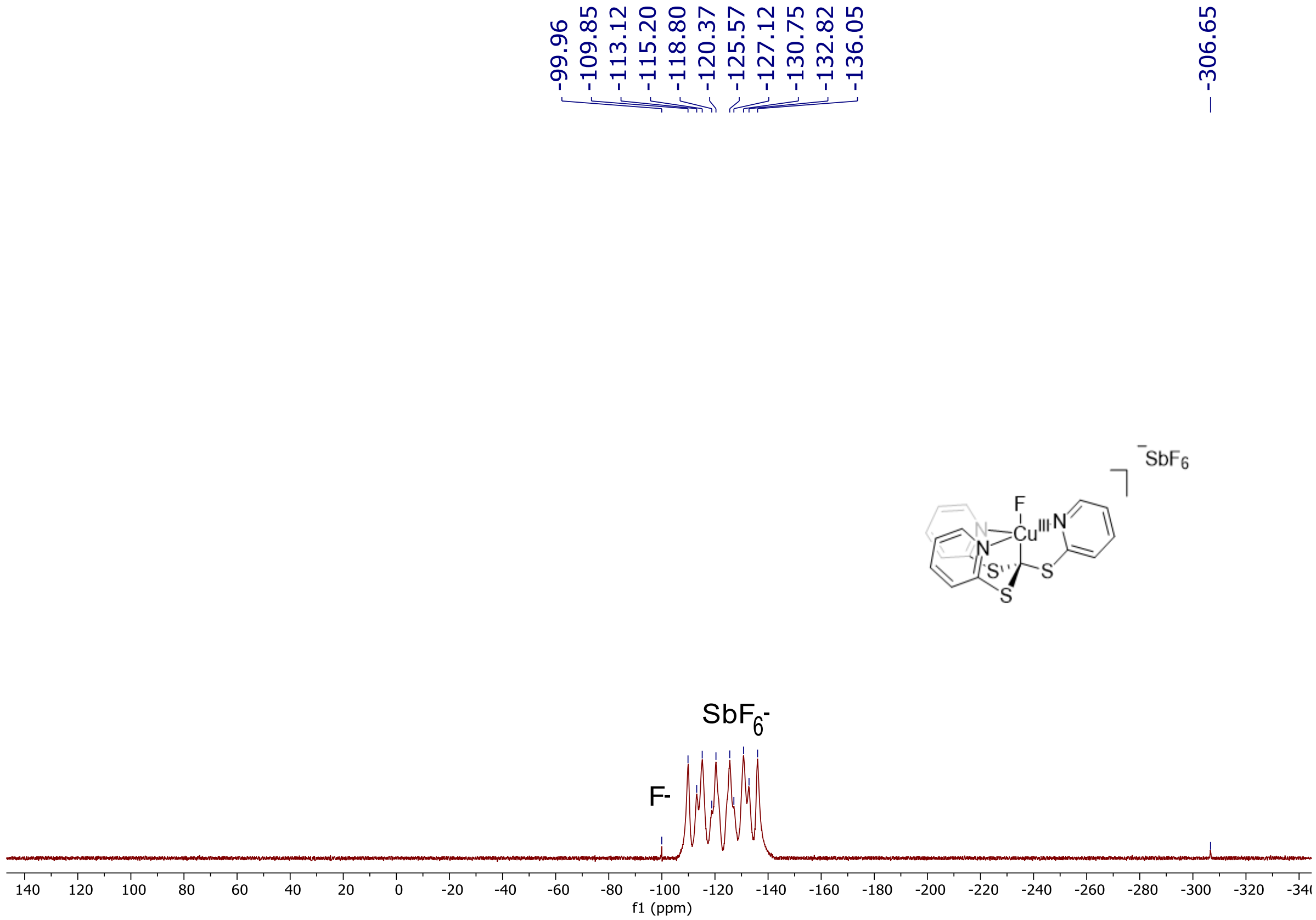

-99.96
-109.85
-113.12
-115.20
-118.80
-120.37
-125.57
-127.12
-130.75
-132.82
-136.05
-306.65
SbF6
F
Cu
N
S
SbF6-
F-
140 120 100 80 60 40 20 0 -20 -40 -60 -80 -100 -120 -140 -160 -180 -200 -220 -240 -260 -280 -300 -320
f1 (ppm)

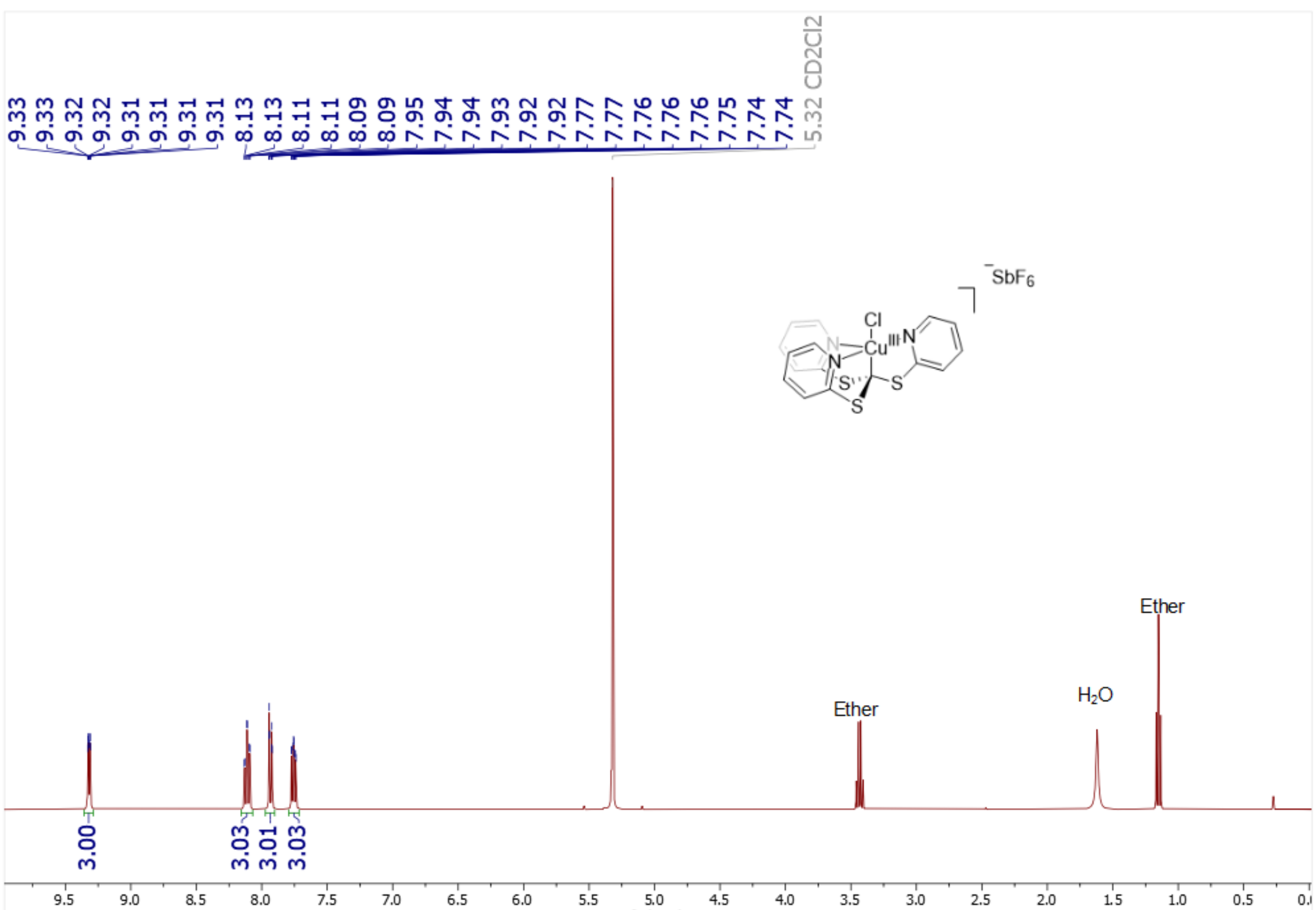

9.33
9.33
9.32
9.32
9.31
9.31
9.31
9.31
8.13
8.13
8.11
8.11
8.09
8.09
7.95
7.94
7.94
7.93
7.92
7.92
7.77
7.77
7.76
7.76
7.76
7.75
7.74
7.74
5.32 CD2Cl2
SbF6
Cl
Cu
N
S
Ether
H2O
Ether
3.00
3.03
3.01
3.03
9.5 9.0 8.5 8.0 7.5 7.0 6.5 6.0 5.5 5.0 4.5 4.0 3.5 3.0 2.5 2.0 1.5 1.0 0.5
f1 (ppm)

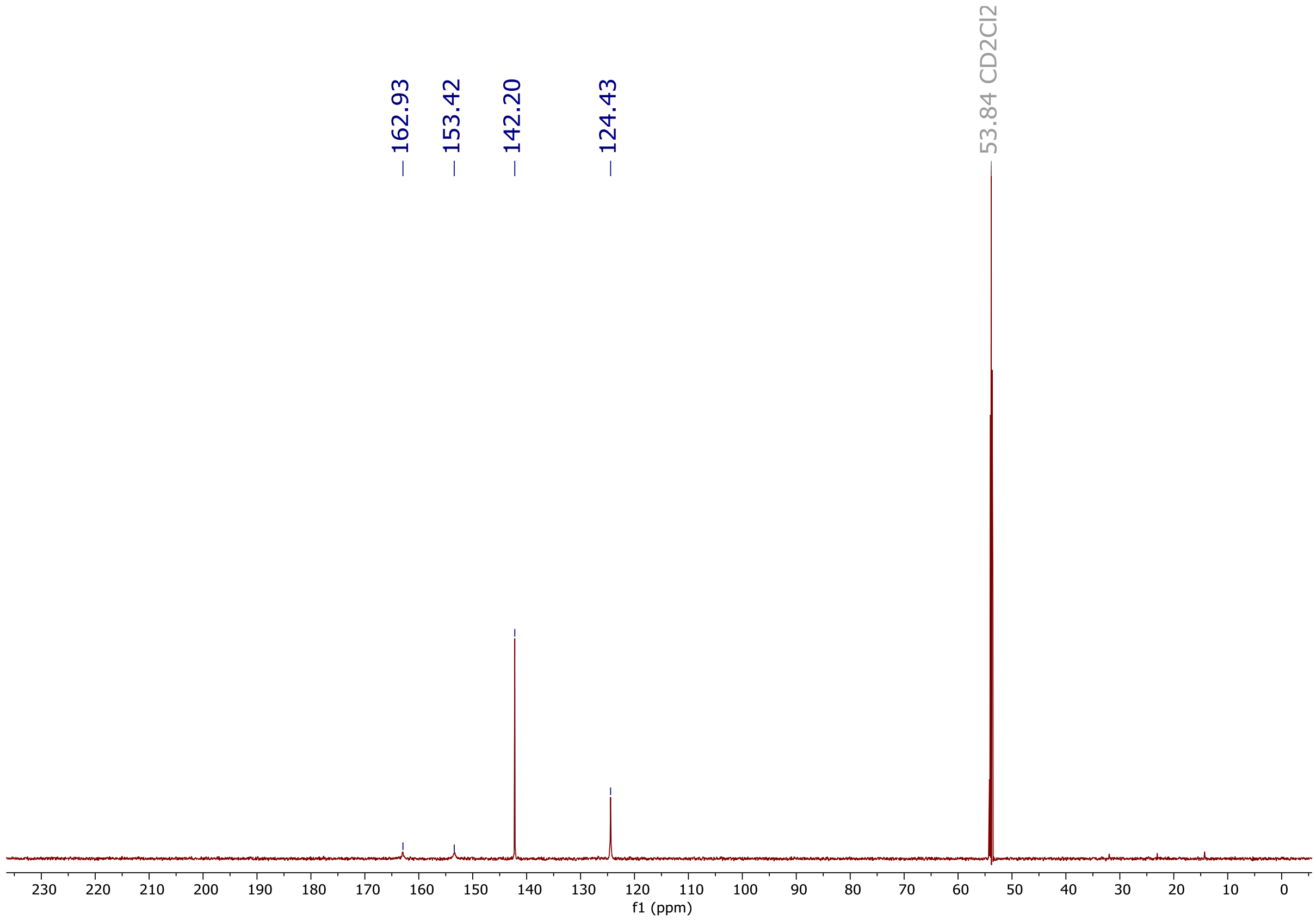

162.93
153.42
142.20
124.43
53.84 CD2Cl2
230 220 210 200 190 180 170 160 150 140 130 120 110 100 90 80 70 60 50 40 30 20 10 0
f1 (ppm)

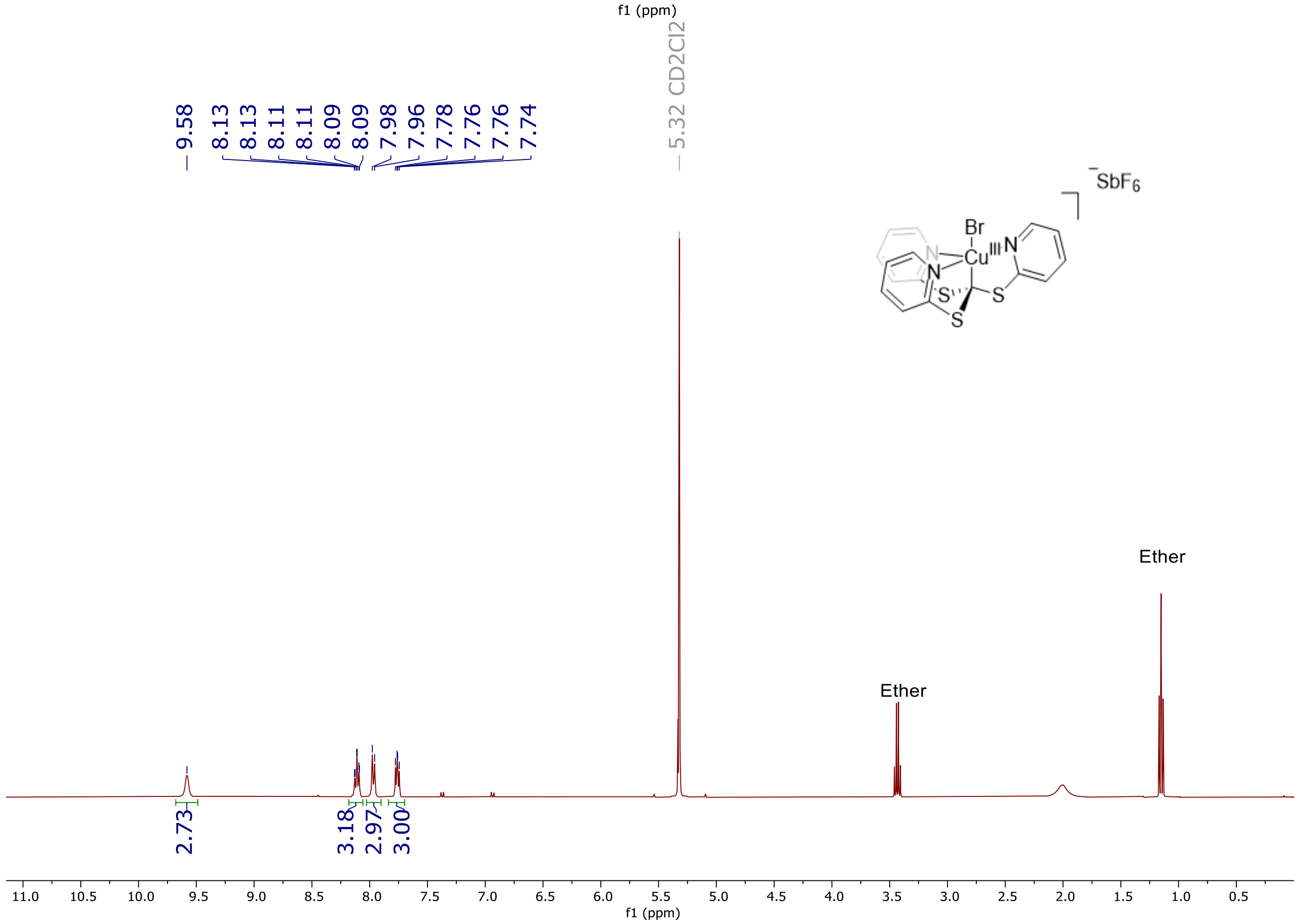

9.58
8.13
8.13
8.11
8.11
8.09
8.09
7.98
7.96
7.78
7.76
7.76
7.74
5.32 CD2Cl2
Br
Cu
N
N
N
S
S
S
SbF6
Ether
Ether
2.73
3.18
2.97
3.00
11.0 10.5 10.0 9.5 9.0 8.5 8.0 7.5 7.0 6.5 6.0 5.5 5.0 4.5 4.0 3.5 3.0 2.5 2.0 1.5 1.0 0.5
f1 (ppm)

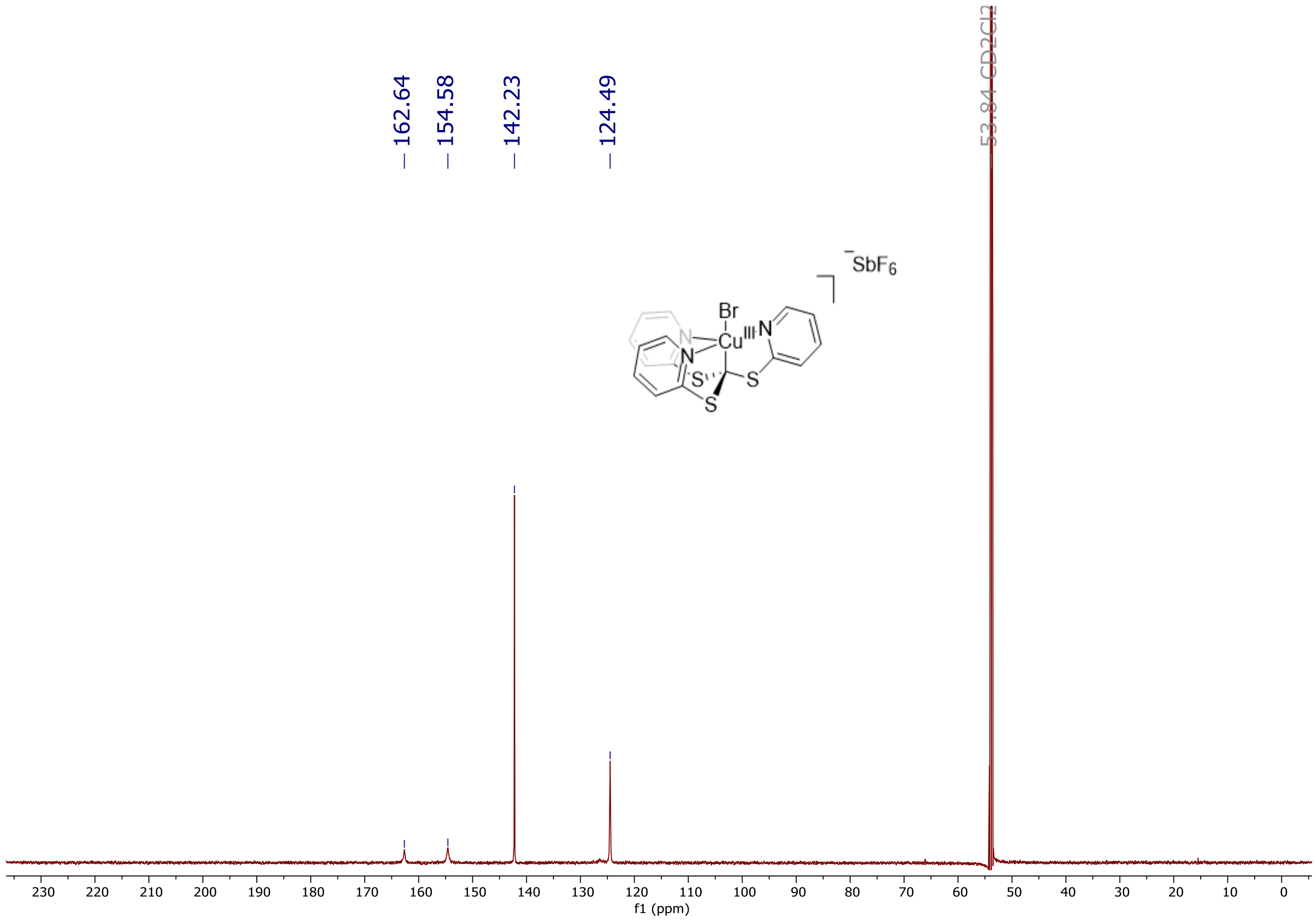

162.64
154.58
142.23
124.49
53.84 CD2Cl2
SbF6
Br
Cu
N
N
N
S
S
S
230
220
210
200
190
180
170
160
150
140
130
120
110
100
90
80
70
60
50
40
30
20
10
0
f1 (ppm)

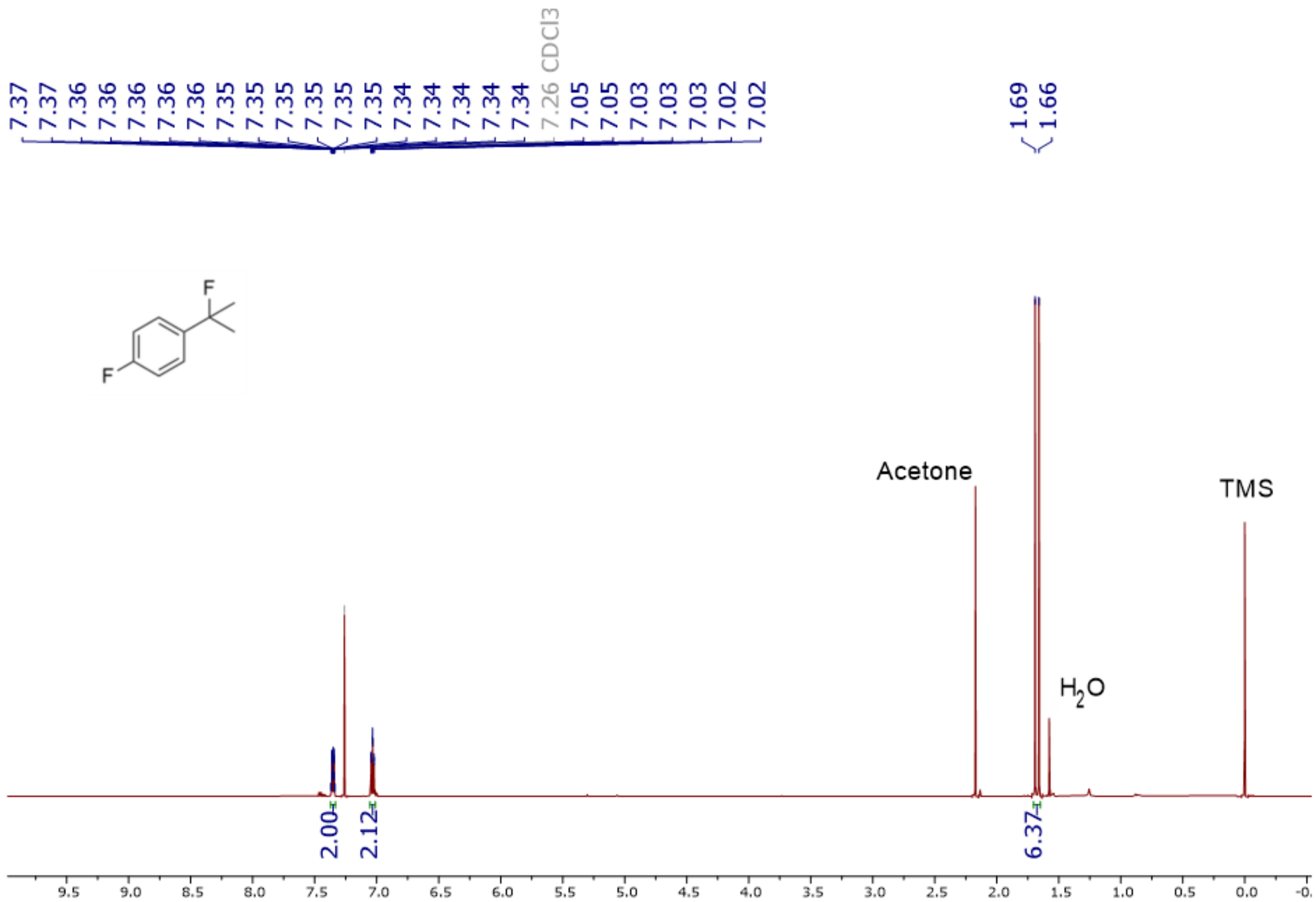
7.37 7.37 7.36 7.36 7.36 7.36 7.36 7.35 7.35 7.35 7.35 7.35 7.35 7.34 7.34 7.34 7.34 7.34 7.26 CDCl3 7.05 7.05 7.03 7.03 7.03 7.02 7.02
1.69 1.66
F
F
Acetone
TMS
H_2O
2.00
2.12
6.37
9.5 9.0 8.5 8.0 7.5 7.0 6.5 6.0 5.5 5.0 4.5 4.0 3.5 3.0 2.5 2.0 1.5 1.0 0.5 0.0

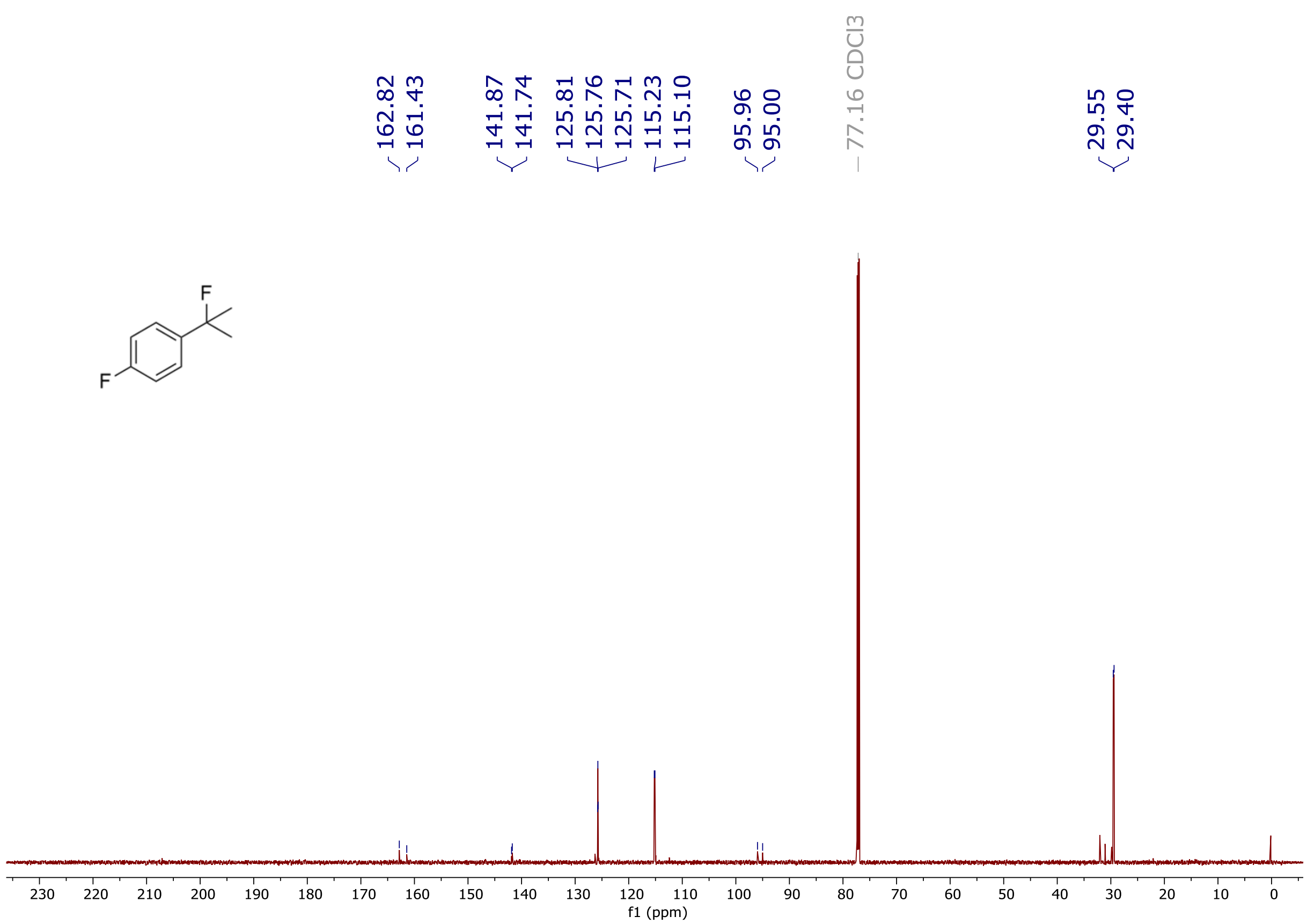
162.82 161.43
141.87 141.74
125.81 125.76 125.71
115.23 115.10
95.96 95.00
77.16 CDCl3
29.55 29.40
F
F
230 220 210 200 190 180 170 160 150 140 130 120 110 100 90 80 70 60 50 40 30 20 10 0
f1 (ppm)

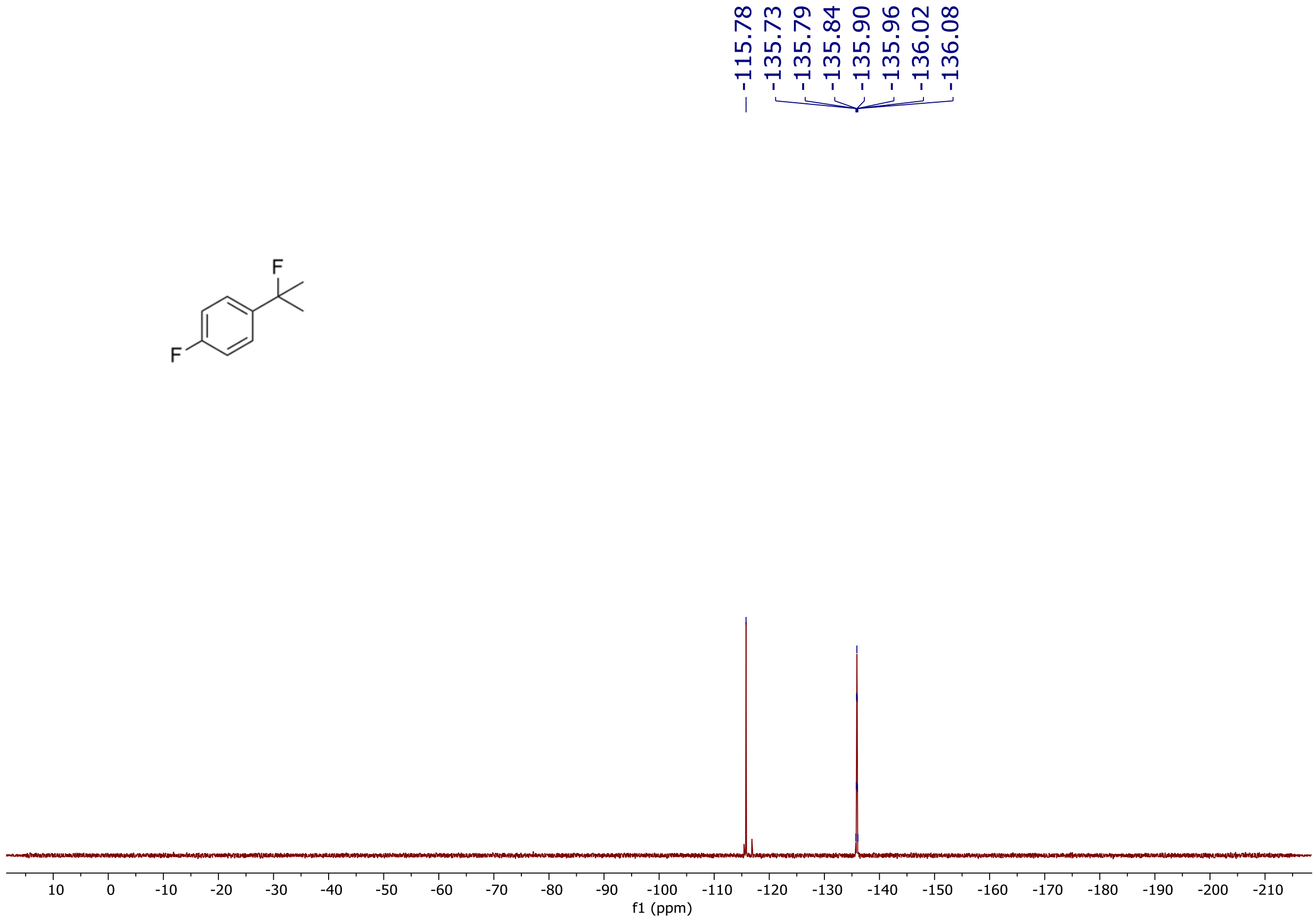
-115.78
-135.73
-135.79
-135.84
-135.90
-135.96
-136.02
-136.08
F
F
10 0 -10 -20 -30 -40 -50 -60 -70 -80 -90 -100 -110 -120 -130 -140 -150 -160 -170 -180 -190 -200 -210
f1 (ppm)

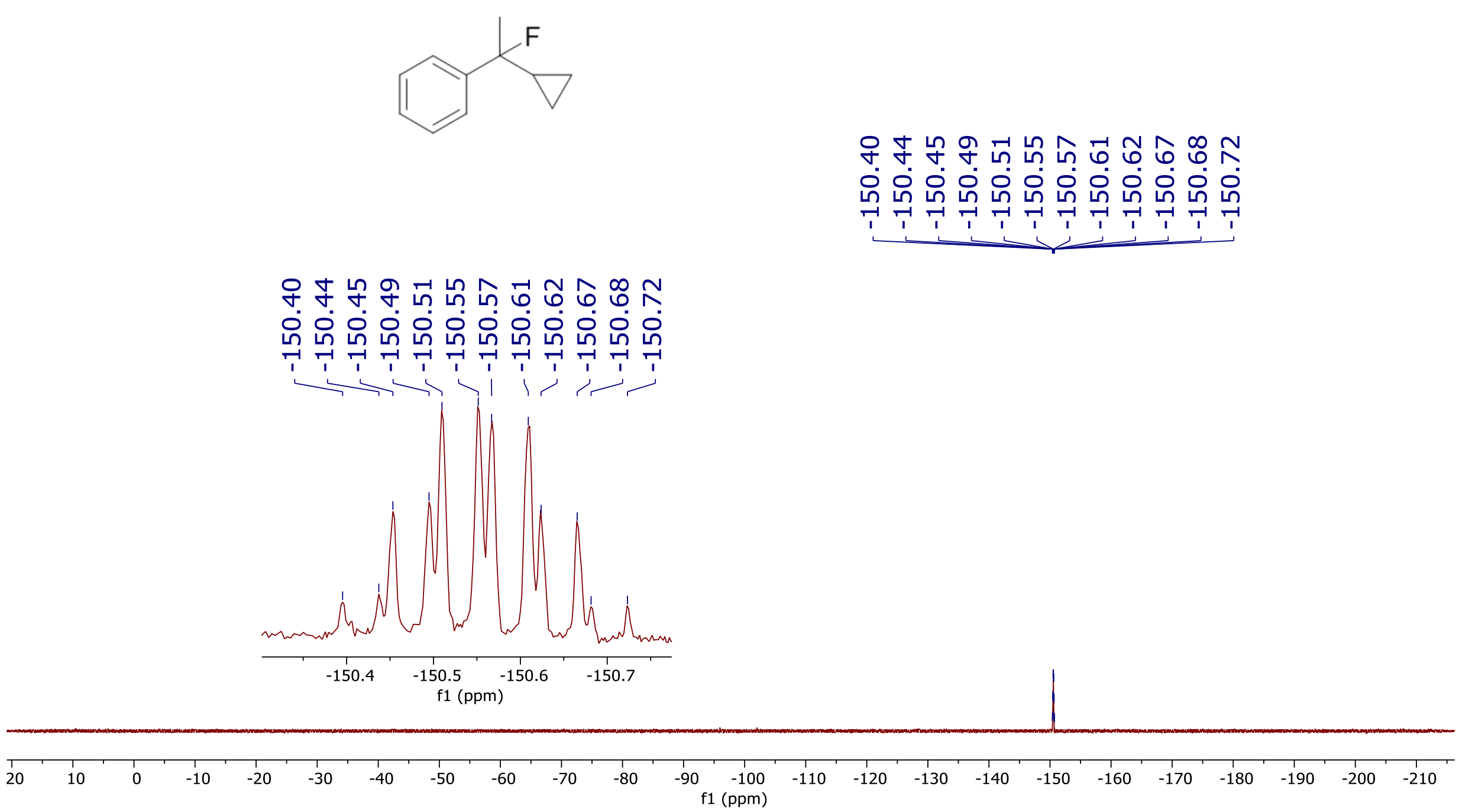
F
-150.40
-150.44
-150.45
-150.49
-150.51
-150.55
-150.57
-150.61
-150.62
-150.67
-150.68
-150.72
-150.4 -150.5 -150.6 -150.7
f1 (ppm)
20 10 0 -10 -20 -30 -40 -50 -60 -70 -80 -90 -100 -110 -120 -130 -140 -150 -160 -170 -180 -190 -200 -210
f1 (ppm)

X-Ray crystallographic details

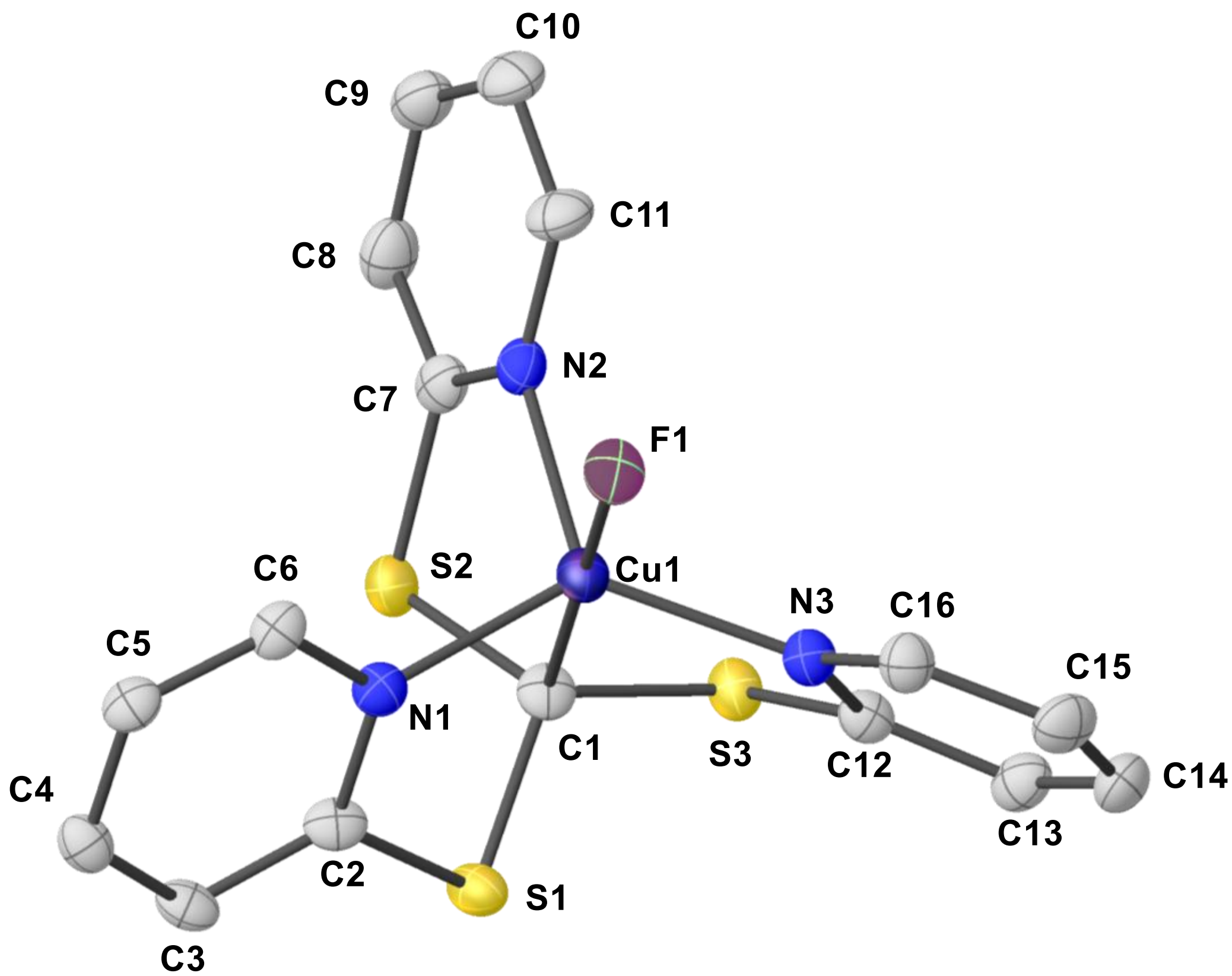

Figure S40. Solid-state structure of the cationic portion of [$\mathbf{L}Cu^{III}F$][SbF_6] with thermal ellipsoids represented at the 50% probability level. Hydrogens, the hexafluoroantimonate anion, and co-crystallized dichloromethane molecules are omitted for clarity. Selected bond distances (Å) and angles (°): Cu1-F1 1.849(19), Cu1-N1 1.999(3), Cu1-N2 2.073(3), Cu1-N3 1.979(3), Cu1-C1 2.000(4), C1-Cu1-F1 178.95(11), CCDC: 2533594

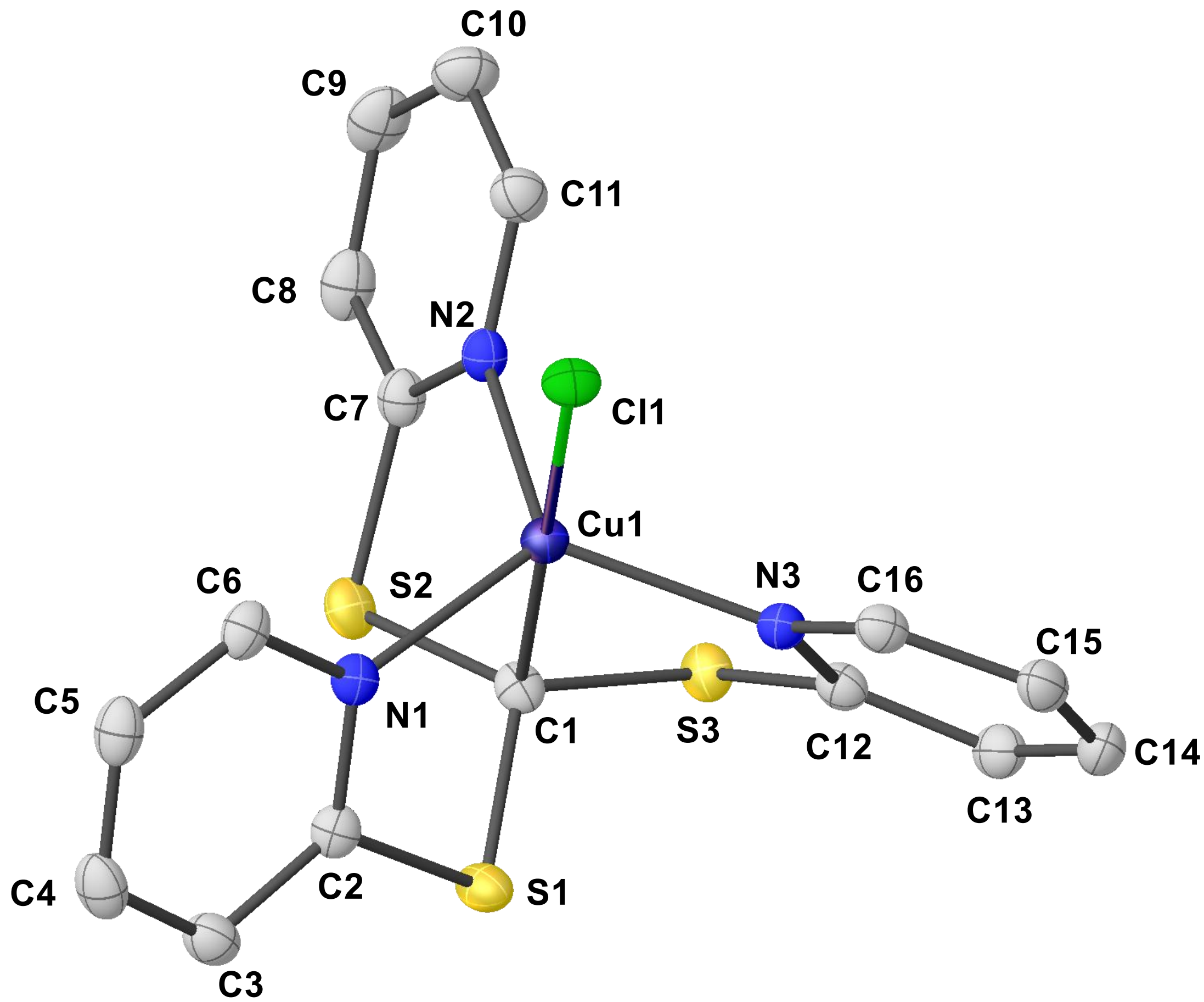

Figure S41. Solid-state structure of the cationic portion of [**L**Cu^{III}Cl][SbF$_6$] with thermal ellipsoids represented at the 50% probability level. Hydrogens, the hexafluoroantimonate anion, and co-crystallized dichloromethane molecules are omitted for clarity. Selected bond distances (Å) and angles (°): Cu1-Cl1 2.2522(5), Cu1-N1 2.038(2), Cu1-N2 2.039(2), Cu1-N3 2.047(2), Cu1-C1 2.034(2), C1-Cu1-Cl1 178.65(6), CCDC: 2533595

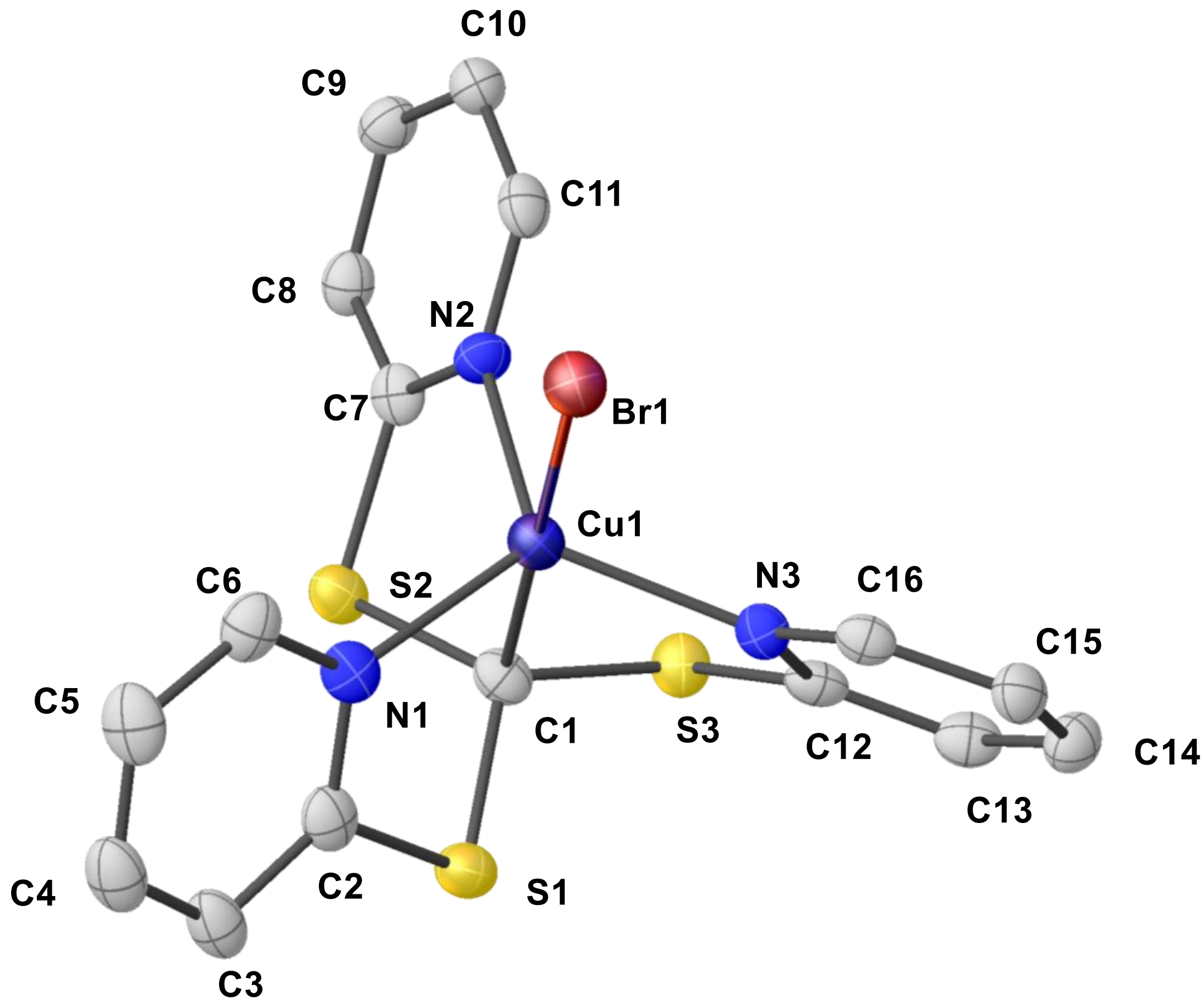

Figure S42. Solid-state structure of the cationic portion of [**L**Cu^{III}Br][SbF_6] with thermal ellipsoids represented at the 50% probability level. Hydrogens, the hexafluoroantimonate anion, and co-crystallized dichloromethane molecules are omitted for clarity. Selected bond distances (Å) and angles (°): Cu1-Br1 2.4084(8), Cu1-N1 2.056(4), Cu1-N2 2.051(4), Cu1-N3 2.054(4), Cu1-C1 2.030(5), C1-Cu1-Br1 179.37(15), CCDC: 2533603

Table S4. Comparison of Select Bond Lengths and Angles for **L**Cu^{II}-F and **L**Cu^{III}-F

	Cu(II)F[4]	Cu(III)F
Cu-F	1.896(9)	1.849(4)
Cu-C	2.02(1)	2.000(4)
Cu-N	2.07(3)	2.017(6)
C-Cu-F	177.0(4)	178.95(11)
N-Cu-F	92.03(4)	94.4(2)
N-Cu-N	101.6(4)	109.54(19)
N-Cu-N	128.2(3)	119.6(2)
N-Cu-N	129.9(3)	129.2(2)

Table S5. Comparison of Select Bond Lengths and Angles for **L**Cu^{II}-Cl and **L**Cu^{III}-Cl

	Cu(II)Cl[22]	Cu(III)Cl
Cu-Cl	2.316(2)	2.257(6)
Cu-C	2.012(9)	2.038(2)
Cu-N	2.11(7)	2.049(2)
C-Cu-Cl	178.1(3)	178.65(6)
N-Cu-Cl	94.1(2)	96.06(5)
N-Cu-N	106.2(2)	118.44(7)
N-Cu-N	122.3(3)	121.17(7)
N-Cu-N	131.5(2)	117.10(7)

Table S6. Select Bond Lengths and Angles for **L**Cu^{III}-Br. **L**Cu^{II}-Br is not reported and attempts to grow single crystals were unsuccessful.

	Cu(III)Br
Cu-Br	2.408(8)
Cu-C	2.030(5)
Cu-N	2.054(4)
C-Cu-Br	179.37(15)
N-Cu-Br	96.1(1)
N-Cu-N	121.34(16)
N-Cu-N	117.59(16)
N-Cu-N	117.75(16)

[**L**Cu^{III}F][SbF_6]

The single crystal X-ray diffraction studies were carried out on a Bruker Kappa Photon III CPAD diffractometer equipped with Mo K_α radiation (λ = 0.71073 Å). A 0.179 × 0.046 × 0.028 mm purple block was mounted on a Cryoloop with Paratone 24EX oil. Data were collected in a nitrogen gas stream at 100 K using φ and ω scans. Crystal-to-detector distance was 60 mm using variable exposure time (2-10s) depending on θ with a scan width of 1.000º. Data collection was 100% complete to 28.325º in θ (0.7490 Å). A total of 45430 reflections were collected covering the indices, $-23 \leq h \leq 23$, $-23 \leq k \leq 23$, $-10 \leq l \leq 7$. 5419 reflections were found to be symmetry independent with an R_{int} of 0.0528. Indexing and unit cell refinement indicated a primitive, tetragonal lattice. The space group was found to be p-4. The data were integrated using the Bruker SAINT software program and scaled using the SADABS software program. Solution by dual-space method (SHELXT) produced a complete phasing model for refinement.[23–28]

All non-hydrogen atoms were refined anisotropically by full-matrix least-squares (SHELXT-2018). All hydrogens were placed using a riding model. Their positions were constrained relative to their parent atom using the appropriate HFIX command in SHELXL-2019. Due to unmodelable solvent disorder, an OLEX2 solvent mask was used to remove the electron density contribution from the disordered solvent from the lattice. Crystal was refined as a 2-component inversion twin (BASF 0.44(2)). Crystallographic data are summarized in Table S3-S10.

Table S7. Crystal data and structure refinement for [**L**Cu^{III}F][SbF_6].

Report date	2026-02-10	
Identification code	[**L**Cu^{III}F][SbF_6]	
Empirical formula	C17 H14 Cl2 Cu F6.75 N3 S3 Sb	
Molecular formula	C16 H12 Cu F N3 S3, F1.25 Sb0.25, 3[F1.5, Sb0.25], 1[C H2 Cl2]	
Formula weight	740.93	
Temperature	100 K	
Wavelength	0.71073 Å	
Crystal system	Tetragonal	
Space group	P4	
Unit cell dimensions	a = 17.8183(6) Å	α = 90°
	b = 17.8183(6) Å	β = 90°
	c = 7.6008(4) Å	γ = 90°
Volume	2413.2(2) $Å^3$	
Z	4	
Density (calculated)	2.039 g/cm^3	
Absorption coefficient (μ)	2.546 mm^{-1}	
F(000)	1439.0	
Crystal size	0.179 × 0.046 × 0.028 mm^3	
Crystal color, habit	Reddish purple block	
Theta range for data collection	2.556 to 28.325°	
Index ranges	$-23 \leq h \leq 23$, $-23 \leq k \leq 23$, $-10 \leq l \leq 7$	
Reflections collected	45430	
Independent reflections	5419 [R(int) = 0.0528, R(sigma) = 0.0320]	
Completeness to theta = 28.325°	99.8 %	
Absorption correction	Multi-scan	
Max. and min. transmission	0.0962 and 0.0634	
Refinement method	Full-matrix least-squares on F^2	

Data / restraints / parameters	5419 / 1 / 314
Goodness-of-fit on F^2	1.042
Final R indexes [I>2signma(I)]	R1 = 0.0310, wR2 = 0.0735
Final R indexes [all data]	R1 = 0.0358, wR2 = 0.0762
Flack parameter	0.44(2)
Largest diff. peak and hole	2.24 and -0.67 e Å^{-3}

Table S8. Fractional Atomic Coordinates (×10^4) and Equivalent Isotropic Displacement Parameters (Å^2×10^3) for [**L**Cu^{III}F][SbF_6]. U_{eq} is defined as 1/3 of the trace of the orthogonalised U_{IJ} tensor.

	x	*y*	*z*	U(eq)
Sb01	0	10000	2837.7(10)	20.03(15)
Sb03	5000	10000	8611.7(9)	25.71(13)
Cu04	2775.6(4)	8424.5(4)	5071.0(10)	20.60(15)
S05	3181.3(8)	8462.6(8)	8932(2)	23.5(3)
S06	1979.6(8)	7449.7(8)	7842(2)	24.0(3)
S07	3532.7(8)	7087.7(8)	6846(2)	23.1(3)
Cl08	3495.5(11)	6772.4(10)	1688(3)	39.9(4)
Cl09	2862.1(14)	5538.3(12)	-339(3)	52.2(5)
F0A	2682.0(19)	8998(2)	3058(4)	26.9(8)
F00B	0	10000	350(10)	30.4(16)
F00C	604(2)	9133(2)	2855(6)	32.7(8)
F00D	4706(2)	11014(2)	8635(7)	46.4(10)
F00E	0	10000	5318(10)	28.7(16)
F00F	4282(3)	9800(3)	10357(7)	49.7(11)
N00G	3836(3)	8166(3)	4579(7)	21.8(10)
F00H	4290(3)	9785(2)	6863(7)	45.6(11)
N00I	1936(3)	7672(3)	4417(7)	22.4(10)
N00J	2360(3)	9216(3)	6659(7)	20.7(9)

Table S8. Fractional Atomic Coordinates (×10[4]) and Equivalent Isotropic Displacement Parameters (Å[2]×10[3]) for [**L**Cu^{III}F][SbF_6]. U_{eq} is defined as 1/3 of the trace of the orthogonalised U_{IJ} tensor.

	x	*y*	*z*	U(eq)
900K	1540(3)	10234(3)	7280(8)	21.9(12)
F1	4253(5)	5732(5)	5142(13)	35.3(18)
C00M	2530(3)	9174(3)	8377(8)	21.1(11)
C00N	1680(3)	7576(3)	2757(9)	28.1(12)
C00O	4155(3)	7600(3)	5494(8)	21.5(11)
C00P	1714(3)	10191(3)	9069(8)	22.5(12)
C00Q	1100(4)	7084(4)	2384(9)	32.9(15)
C00R	4902(3)	7393(3)	5282(9)	26.5(12)
C00S	4257(3)	8548(3)	3419(9)	23.6(11)
C00T	1637(3)	7268(3)	5703(9)	23.3(11)
C00U	781(3)	6690(3)	3762(10)	31.5(14)
C00V	2215(3)	9644(3)	9653(8)	23.2(12)
C00W	2878(3)	7827(3)	7280(7)	21.4(11)
C00X	1865(3)	9742(3)	6125(8)	21.1(11)
C00Y	5003(3)	8366(3)	3112(9)	27.9(13)
C00Z	5325(3)	7786(3)	4054(9)	28.2(13)
C010	1051(3)	6766(3)	5438(10)	30.9(14)
C011	3655(4)	5878(4)	769(12)	44.9(18)
Sb	5000	5000	5663.8(10)	21.14(16)
F3	4819(13)	4970(50)	3230(20)	15(7)
F4	4768(11)	4970(30)	8100(30)	22(7)
F2	4275(5)	5688(5)	6110(15)	48(2)

Table S9. Anisotropic Displacement Parameters (Å^2×10^3) for [**L**Cu^{III}F][SbF$_6$]. The Anisotropic displacement factor exponent takes the form: $-2\pi^2[h^2a^{*2}U_{11}+2hka^*b^*U_{12}+\ldots]$.

	U_{11}	U_{22}	U_{33}	U_{23}	U_{13}	U_{12}
Sb01	20.0(2)	20.0(2)	20.1(4)	0	0	0
Sb03	28.5(3)	21.4(2)	27.2(3)	0	0	1.8(2)
Cu04	22.0(3)	23.0(3)	16.8(3)	2.7(3)	0.3(3)	2.7(3)
S05	28.1(7)	21.6(6)	20.9(7)	-1.0(6)	-5.1(6)	4.4(5)
S06	24.3(6)	24.9(6)	22.7(7)	0.4(6)	4.0(6)	0.2(5)
S07	26.4(7)	21.4(6)	21.4(7)	3.3(6)	2.2(6)	4.6(5)
Cl08	52.5(10)	36.6(8)	30.7(9)	-6.5(7)	2.1(8)	-5.3(7)
Cl09	78.9(15)	47.2(11)	30.4(9)	-2.4(8)	0.2(10)	-25.9(10)
F0A	29.9(17)	34.0(18)	16.8(18)	9.5(14)	1.1(14)	4.4(14)
F00B	36(2)	36(2)	18(4)	0	0	0
F00C	35.8(19)	29.7(18)	32(2)	-4.1(17)	-2.6(18)	8.1(15)
F00D	64(3)	22.1(18)	53(3)	-6(2)	3(3)	8.9(17)
F00E	29(2)	29(2)	28(4)	0	0	0
F00F	60(3)	49(3)	40(3)	-2(2)	17(2)	-5(2)
N00G	21(2)	23(2)	21(2)	-0.3(19)	1.4(19)	0.8(19)
F00H	49(3)	38(2)	49(3)	-2(2)	-17(2)	1.0(19)
N00I	20(2)	25(2)	23(3)	-1(2)	0.3(19)	3.6(19)
N00J	22(2)	21(2)	19(2)	3(2)	-1(2)	0.1(17)
C00K	21(3)	19(3)	26(3)	4(2)	-4(2)	1(2)
C00M	25(3)	19(2)	19(3)	5(2)	-4(2)	-2(2)
C00N	26(3)	31(3)	26(3)	4(3)	-8(3)	1(2)
C00O	24(3)	21(2)	19(3)	-1(2)	1(2)	-1(2)
C00P	27(3)	20(3)	21(3)	-2(2)	2(2)	0(2)
C00Q	27(3)	36(3)	35(4)	-6(3)	-13(3)	8(3)
C00R	24(3)	23(3)	33(3)	0(3)	-3(2)	2(2)

Table S9. Anisotropic Displacement Parameters ($Å^2×10^3$) for [**L**Cu^{III}F][SbF_6]. The Anisotropic displacement factor exponent takes the form: $-2\pi^2[h^2a^{*2}U_{11}+2hka^*b^*U_{12}+\ldots]$.

	U_{11}	U_{22}	U_{33}	U_{23}	U_{13}	U_{12}
C00S	23(3)	24(3)	24(3)	1(2)	2(2)	0(2)
C00T	22(3)	18(2)	29(3)	-2(2)	0(2)	3.1(19)
C00U	23(3)	28(3)	43(4)	-9(3)	-3(3)	0(2)
C00V	31(3)	22(3)	16(3)	2(2)	-2(2)	-1(2)
C00W	25(3)	22(3)	18(3)	4(2)	-1(2)	2(2)
C00X	20(2)	19(2)	24(3)	5(2)	-1(2)	1(2)
C00Y	22(3)	31(3)	31(4)	1(2)	2(2)	-8(2)
C00Z	18(3)	34(3)	32(3)	-3(3)	1(2)	-1(2)
C010	23(3)	29(3)	41(4)	-1(3)	6(3)	-3(2)
C011	48(4)	46(4)	41(4)	-3(4)	-11(4)	14(3)
Sb	19.5(2)	19.5(2)	24.4(4)	0	0	0

Table S10. Bond Lengths for [**L**Cu^{III}F][SbF_6].

		Length/Å			Length/Å
Sb01	F00B	1.891(7)	C00K	C00X	1.369(8)
Sb01	F00C	1.883(3)	F1	Sb	1.905(8)
Sb01	F00C[1]	1.883(3)	F1	F2	0.741(11)
Sb01	F00C[2]	1.883(3)	C00M	C00V	1.399(8)
Sb01	F00C[3]	1.883(3)	C00N	C00Q	1.385(9)
Sb01	F00E	1.885(8)	C00O	C00R	1.391(8)
Sb03	F00D[4]	1.881(4)	C00P	C00V	1.394(8)
Sb03	F00D	1.881(4)	C00Q	C00U	1.383(10)
Sb03	F00F	1.877(5)	C00R	C00Z	1.389(9)
Sb03	F00F[4]	1.877(5)	C00S	C00Y	1.387(8)

Table S10. Bond Lengths for [**L**Cu^{III}F][SbF_6].

		Length/Å			Length/Å
Sb03	F00H[4]	1.875(5)	C00T	C010	1.390(8)
Sb03	F00H	1.875(5)	C00U	C010	1.368(10)
Cu04	F0A	1.847(3)	C00Y	C00Z	1.382(9)
Cu04	N00G	1.980(5)	Sb	F3[5]	1.875(17)
Cu04	N00I	2.069(5)	Sb	F3[6]	1.875(17)
Cu04	N00J	1.999(5)	Sb	F3[7]	1.875(17)
Cu04	C00W	1.996(6)	Sb	F3	1.875(17)
S05	C00M	1.769(6)	Sb	F4[7]	1.90(2)
S05	C00W	1.775(6)	Sb	F4[5]	1.90(2)
S06	C00T	1.766(7)	Sb	F4[6]	1.90(2)
S06	C00W	1.788(6)	Sb	F4	1.90(2)
S07	C00O	1.765(6)	Sb	F2[6]	1.812(10)
S07	C00W	1.791(6)	Sb	F2	1.812(10)
Cl08	C011	1.764(8)	Sb	F2[5]	1.812(10)
Cl09	C011	1.753(8)	Sb	F2[7]	1.812(10)
N00G	C00O	1.351(7)	F3	F3[6]	0.46(3)
N00G	C00S	1.343(7)	F3	F3[7]	0.65(4)
N00I	C00N	1.353(8)	F3	F3[5]	0.46(3)
N00I	C00T	1.326(8)	F4	F4[5]	0.59(3)
N00J	C00M	1.343(8)	F4	F4[6]	0.59(3)
N00J	C00X	1.349(7)	F4	F4[7]	0.83(4)
C00K	C00P	1.397(8)			

[1]1-Y,1+X,+Z; [2]-X,2-Y,+Z; [3]-1+Y,1-X,+Z; [4]1-X,2-Y,+Z; [5]1-X,1-Y,+Z; [6]1-Y,+X,+Z; [7]+Y,1-X,+Z

Table S11. Bond Angles for **[L**Cu^{III}F][SbF$_6$].

			Angle/°				Angle/°
F00C^1	Sb01	F00B	90.41(14)	Cl09	C011	Cl08	111.8(4)
F00C^2	Sb01	F00B	90.41(14)	F3^5	Sb	F3	14.1(9)
F00C^3	Sb01	F00B	90.41(14)	F3^6	Sb	F3^5	20.0(12)
F00C	Sb01	F00B	90.41(14)	F3^6	Sb	F3	14.1(9)
F00C^2	Sb01	F00C^1	89.997(4)	F3^5	Sb	F3^7	14.1(9)
F00C	Sb01	F00C^1	179.2(3)	F3^6	Sb	F3^7	14.1(9)
F00C^3	Sb01	F00C^1	89.997(3)	F3^7	Sb	F3	20.0(12)
F00C	Sb01	F00C^2	89.997(4)	F3^6	Sb	F4	164(3)
F00C^2	Sb01	F00C^3	179.2(3)	F3^7	Sb	F4^7	157.3(8)
F00C	Sb01	F00C^3	89.997(5)	F3^7	Sb	F4	177.3(9)
F00C^3	Sb01	F00E	89.59(14)	F3	Sb	F4^5	164(3)
F00C^1	Sb01	F00E	89.59(14)	F3^5	Sb	F4^6	177.3(9)
F00C	Sb01	F00E	89.59(14)	F3	Sb	F4^6	164(3)
F00C^2	Sb01	F00E	89.59(14)	F3^5	Sb	F4	164(3)
F00E	Sb01	F00B	180.0	F3^5	Sb	F4^7	164(3)
F00D^4	Sb03	F00D	178.9(3)	F3^6	Sb	F4^5	177.3(9)
F00F^4	Sb03	F00D	90.1(2)	F3	Sb	F4	157.3(8)
F00F^4	Sb03	F00D^4	89.2(2)	F3^7	Sb	F4^5	164(3)
F00F	Sb03	F00D	89.2(2)	F3	Sb	F4^7	177.3(9)
F00F	Sb03	F00D^4	90.1(2)	F3^6	Sb	F4^6	157.3(8)
F00F	Sb03	F00F^4	90.1(3)	F3^6	Sb	F4^7	164(3)
F00H	Sb03	F00D^4	89.9(2)	F3^7	Sb	F4^6	164(3)
F00H^4	Sb03	F00D	89.9(2)	F3^5	Sb	F4^5	157.3(8)
F00H^4	Sb03	F00D^4	90.9(2)	F4	Sb	F4^7	25.3(11)
F00H	Sb03	F00D	90.9(2)	F4^5	Sb	F4^6	25.3(11)

Table S11. Bond Angles for [**L**Cu^{III}F][SbF_6].

			Angle/°				Angle/°
F00H	Sb03	F00F	90.1(2)	F4	Sb	F4^{5}	17.8(8)
F00H^{4}	Sb03	F00F^{4}	90.1(2)	F4^{7}	Sb	F4^{5}	17.8(8)
F00H^{4}	Sb03	F00F	179.1(2)	F4^{7}	Sb	F4^{6}	17.8(8)
F00H	Sb03	F00F^{4}	179.1(2)	F4	Sb	F4^{6}	17.8(8)
F00H	Sb03	F00H^{4}	89.7(3)	F2	Sb	F3^{5}	92.9(18)
F0A	Cu04	N00G	93.34(18)	F2^{5}	Sb	F3^{5}	94(2)
F0A	Cu04	N00I	95.42(19)	F2^{6}	Sb	F3^{7}	108.5(19)
F0A	Cu04	N00J	94.38(18)	F2	Sb	F3^{6}	108.5(19)
F0A	Cu04	C00W	178.6(2)	F2	Sb	F3	94(2)
N00G	Cu04	N00I	119.6(2)	F2^{5}	Sb	F3	108.5(19)
N00G	Cu04	N00J	129.2(2)	F2^{5}	Sb	F3^{7}	92.9(18)
N00G	Cu04	C00W	87.0(2)	F2^{7}	Sb	F3^{7}	94(2)
N00J	Cu04	N00I	109.54(19)	F2^{6}	Sb	F3	92.9(18)
C00W	Cu04	N00I	85.5(2)	F2	Sb	F3^{7}	107(2)
C00W	Cu04	N00J	84.4(2)	F2^{7}	Sb	F3^{5}	108.5(19)
C00M	S05	C00W	95.1(3)	F2^{7}	Sb	F3	107(2)
C00T	S06	C00W	99.1(3)	F2^{6}	Sb	F3^{6}	94(2)
C00O	S07	C00W	97.8(3)	F2^{5}	Sb	F3^{6}	107(2)
C00O	N00G	Cu04	118.5(4)	F2^{7}	Sb	F3^{6}	92.9(18)
C00S	N00G	Cu04	122.7(4)	F2^{6}	Sb	F3^{5}	107(2)
C00S	N00G	C00O	118.7(5)	F2^{7}	Sb	F4	87.4(15)
C00N	N00I	Cu04	123.4(4)	F2^{7}	Sb	F4^{5}	89.1(13)
C00T	N00I	Cu04	117.7(4)	F2	Sb	F4	71.4(16)
C00T	N00I	C00N	118.9(5)	F2^{5}	Sb	F4^{6}	87.4(15)
C00M	N00J	Cu04	117.7(4)	F2	Sb	F4^{7}	87.4(15)

Table S11. Bond Angles for **[L**Cu^{III}F][SbF$_6$].

			Angle/°				Angle/°
C00M	N00J	C00X	118.6(5)	F2	Sb	F4^6	89.1(13)
C00X	N00J	Cu04	123.4(4)	F2^6	Sb	F4	69.6(14)
C00X	C00K	C00P	119.6(5)	F2^7	Sb	F4^7	71.4(16)
F2	F1	Sb	71.5(11)	F2^6	Sb	F4^6	71.4(16)
N00J	C00M	S05	114.8(4)	F2^5	Sb	F4^7	69.6(14)
N00J	C00M	C00V	123.4(5)	F2^6	Sb	F4^7	89.1(13)
C00V	C00M	S05	121.8(4)	F2^7	Sb	F4^6	69.6(14)
N00I	C00N	C00Q	121.5(6)	F2^5	Sb	F4	89.1(13)
N00G	C00O	S07	115.0(4)	F2	Sb	F4^5	69.6(14)
N00G	C00O	C00R	122.7(5)	F2^5	Sb	F4^5	71.4(16)
C00R	C00O	S07	122.2(4)	F2^6	Sb	F4^5	87.4(15)
C00V	C00P	C00K	119.4(5)	F2^5	Sb	F2^7	87.99(14)
C00U	C00Q	C00N	118.2(6)	F2^6	Sb	F2	87.99(14)
C00Z	C00R	C00O	117.7(6)	F2^7	Sb	F2	158.4(7)
N00G	C00S	C00Y	121.9(6)	F2^6	Sb	F2^7	87.99(13)
N00I	C00T	S06	116.1(4)	F2^5	Sb	F2	87.99(14)
N00I	C00T	C010	123.0(6)	F2^5	Sb	F2^6	158.4(7)
C010	C00T	S06	120.7(5)	F3^5	F3	Sb	82.9(4)
C010	C00U	C00Q	120.7(6)	F3^7	F3	Sb	80.0(6)
C00P	C00V	C00M	117.0(5)	F3^6	F3	Sb	82.9(4)
S05	C00W	Cu04	106.4(3)	F3^6	F3	F3^5	90.01(13)
S05	C00W	S06	110.1(3)	F3^6	F3	F3^7	45.00(6)
S05	C00W	S07	113.7(3)	F3^5	F3	F3^7	45.00(3)
S06	C00W	Cu04	108.6(3)	F4^7	F4	Sb	77.3(6)
S06	C00W	S07	110.5(3)	F4^5	F4	Sb	81.1(4)

Table S11. Bond Angles for [**L**$Cu^{III}F$][SbF_6].

			Angle/°				Angle/°
S07	C00W	Cu04	107.3(3)	F4[6]	F4	Sb	81.1(4)
N00J	C00X	C00K	121.9(5)	F4[5]	F4	F4[7]	45.00(2)
C00Z	C00Y	C00S	119.0(6)	F4[6]	F4	F4[7]	45.00(2)
C00Y	C00Z	C00R	120.0(6)	F4[6]	F4	F4[5]	90.00(6)
C00U	C010	C00T	117.6(6)	F1	F2	Sb	85.6(12)

[1]-X,2-Y,+Z; [2]-1+Y,1-X,+Z; [3]1-Y,1+X,+Z; [4]1-X,2-Y,+Z; [5]+Y,1-X,+Z; [6]1-X,1-Y,+Z; [7]1-Y,+X,+Z

Table S12. Torsion Angles for [**L**$Cu^{III}F$][SbF_6].

				Angle/°					Angle/°
Cu04	N00G	C00O	S07	7.2(6)	F3[1]	Sb	F4	F4[1]	95(8)
Cu04	N00G	C00O	C00R	-177.9(5)	F3[2]	Sb	F4	F4[3]	-141(8)
Cu04	N00G	C00S	C00Y	179.0(5)	F3[2]	Sb	F4	F4[1]	173(8)
Cu04	N00I	C00N	C00Q	-177.8(4)	F3[2]	Sb	F4	F4[2]	-96(8)
Cu04	N00I	C00T	S06	2.0(6)	F3	Sb	F4	F4[2]	-135(9)
Cu04	N00I	C00T	C010	177.4(4)	F3[1]	Sb	F4	F4[3]	141(8)
Cu04	N00J	C00M	S05	5.9(6)	F3[2]	Sb	F2	F1	4(3)
Cu04	N00J	C00M	C00V	-173.1(4)	F3	Sb	F2	F1	-10(2)
Cu04	N00J	C00X	C00K	174.1(4)	F3[1]	Sb	F2	F1	-9(3)
S05	C00M	C00V	C00P	179.4(4)	F3[3]	Sb	F2	F1	6(2)
S06	C00T	C010	C00U	175.7(4)	F4	Sb	F3	F3[2]	134(11)
S07	C00O	C00R	C00Z	173.6(5)	F4[2]	Sb	F3	F3[1]	173(12)
N00G	C00O	C00R	C00Z	-1.0(9)	F4	Sb	F3	F3[3]	180(11)
N00G	C00S	C00Y	C00Z	-1.4(9)	F4[2]	Sb	F3	F3[2]	82(12)
N00I	C00N	C00Q	C00U	0.2(9)	F4[2]	Sb	F3	F3[3]	127(12)

Table S12. Torsion Angles for [$\mathbf{L}Cu^{III}F$][SbF_6].

				Angle/°					Angle/°
N00I	C00T	C010	C00U	0.5(9)	$F4^1$	Sb	F3	$F3^3$	-128(12)
N00J	C00M	C00V	C00P	-1.7(9)	F4	Sb	F3	$F3^1$	-135(11)
C00K	C00P	C00V	C00M	1.4(8)	$F4^1$	Sb	F3	$F3^2$	-174(12)
C00M	S05	C00W	Cu04	45.7(3)	$F4^1$	Sb	F3	$F3^1$	-83(12)
C00M	S05	C00W	S06	-71.9(3)	$F4^3$	Sb	F4	$F4^1$	-45.70(8)
C00M	S05	C00W	S07	163.5(3)	$F4^1$	Sb	F4	$F4^3$	45.70(6)
C00M	N00J	C00X	C00K	0.6(8)	$F4^3$	Sb	F4	$F4^2$	45.70(7)
C00N	N00I	C00T	S06	-177.9(4)	$F4^2$	Sb	F4	$F4^1$	-91.41(13)
C00N	N00I	C00T	C010	-2.4(9)	$F4^2$	Sb	F4	$F4^3$	-45.70(6)
C00N	C00Q	C00U	C010	-2.1(9)	$F4^1$	Sb	F4	$F4^2$	91.41(13)
C00O	S07	C00W	Cu04	37.7(3)	$F4^3$	Sb	F2	F1	168.0(17)
C00O	S07	C00W	S05	-79.6(3)	$F4^2$	Sb	F2	F1	169(2)
C00O	S07	C00W	S06	156.0(3)	F4	Sb	F2	F1	-172.0(18)
C00O	N00G	C00S	C00Y	1.3(9)	$F4^1$	Sb	F2	F1	-174.3(19)
C00O	C00R	C00Z	C00Y	0.9(9)	$F2^2$	Sb	F3	$F3^1$	86(17)
C00P	C00K	C00X	N00J	-0.8(9)	$F2^3$	Sb	F3	$F3^3$	-53(16)
C00Q	C00U	C010	C00T	1.8(9)	F2	Sb	F3	$F3^3$	130(16)
C00S	N00G	C00O	S07	-175.0(4)	F2	Sb	F3	$F3^1$	175(16)
C00S	N00G	C00O	C00R	-0.1(9)	$F2^2$	Sb	F3	$F3^2$	-5(17)
C00S	C00Y	C00Z	C00R	0.2(9)	$F2^2$	Sb	F3	$F3^3$	40(16)
C00T	S06	C00W	Cu04	35.8(3)	F2	Sb	F3	$F3^2$	84(16)
C00T	S06	C00W	S05	152.0(3)	$F2^1$	Sb	F3	$F3^2$	172(16)
C00T	S06	C00W	S07	-81.6(3)	$F2^3$	Sb	F3	$F3^1$	-8(16)
C00T	N00I	C00N	C00Q	2.1(9)	$F2^1$	Sb	F3	$F3^1$	-97(16)
C00W	S05	C00M	N00J	-32.9(5)	$F2^3$	Sb	F3	$F3^2$	-99(16)

Table S12. Torsion Angles for [**L**$Cu^{III}F$][SbF_6].

				Angle/°					Angle/°
C00W	S05	C00M	C00V	146.1(5)	F2[1]	Sb	F3	F3[3]	-142(16)
C00W	S06	C00T	N00I	-24.0(5)	F2	Sb	F4	F4[2]	-81(9)
C00W	S06	C00T	C010	160.4(5)	F2[1]	Sb	F4	F4[3]	138(9)
C00W	S07	C00O	N00G	-28.9(5)	F2[3]	Sb	F4	F4[3]	49(8)
C00W	S07	C00O	C00R	156.2(5)	F2[1]	Sb	F4	F4[1]	93(9)
C00X	N00J	C00M	S05	179.7(4)	F2	Sb	F4	F4[1]	-172(9)
C00X	N00J	C00M	C00V	0.7(8)	F2[1]	Sb	F4	F4[2]	-176(9)
C00X	C00K	C00P	C00V	-0.2(9)	F2[3]	Sb	F4	F4[1]	4(8)
F3[1]	Sb	F3	F3[2]	-90.88(14)	F2	Sb	F4	F4[3]	-127(9)
F3[2]	Sb	F3	F3[3]	45.44(6)	F2[2]	Sb	F4	F4[2]	7(8)
F3[2]	Sb	F3	F3[1]	90.88(13)	F2[2]	Sb	F4	F4[3]	-39(8)
F3[1]	Sb	F3	F3[3]	-45.44(9)	F2[3]	Sb	F4	F4[2]	95(8)
F3[3]	Sb	F3	F3[1]	45.44(10)	F2[2]	Sb	F4	F4[1]	-84(8)
F3[3]	Sb	F3	F3[2]	-45.44(6)	F2[3]	Sb	F2	F1	177.7(12)
F3	Sb	F4	F4[1]	134(9)	F2[2]	Sb	F2	F1	98.3(12)
F3[1]	Sb	F4	F4[2]	-173(8)	F2[1]	Sb	F2	F1	-102.9(12)
F3	Sb	F4	F4[3]	180(9)					

[1]1-Y,+X,+Z; [2]+Y,1-X,+Z; [3]1-X,1-Y,+Z

Table S13. Hydrogen Atom Coordinates (Å×10^4) and Isotropic Displacement Parameters (Å^2×10^3) for [**L**$Cu^{III}F$][SbF_6].

	x	*y*	*z*	U(eq)
H00K	1197.87	10603.5	6866.88	26
H00N	1903.62	7851.94	1824.75	34
H00P	1492.91	10530.51	9880.2	27

Table S13. Hydrogen Atom Coordinates (Å×10^4) and Isotropic Displacement Parameters (Å^2×10^3) for [**L**Cu^{III}F][SbF_6].

	x	*y*	*z*	U(eq)
H00Q	924.42	7019.81	1213.37	39
H00R	5115.59	6997.76	5952.76	32
H00S	4038.48	8953.53	2791.37	28
H00U	371.17	6362.65	3543.05	38
H00V	2336.66	9592.41	10864.35	28
H00X	1737.8	9771.41	4913.41	25
H00Y	5287.46	8636.84	2266.33	33
H00Z	5835.12	7656.05	3860.86	34
H010	844.91	6486.39	6386.1	37
H01A	4081.51	5906.64	-63.54	54
H01B	3793.27	5522.42	1716.74	54

Table S14. Atomic Occupancy for [**L**Cu^{III}F][SbF_6].

	Occupancy		*Occupancy*		*Occupancy*
F1	0.5	F3	0.125	F4	0.125
F2	0.5				

[**L**Cu^{III}Cl][SbF_6]

The single crystal X-ray diffraction studies were carried out on a Bruker Kappa Photon III CPAD diffractometer equipped with Mo K_α radiation (λ = 0.71073 Å). A 0.048 × 0.081 × 0.202 mm deep purple needle was mounted on a Cryoloop with Paratone 24EX oil. Data were collected in a nitrogen gas stream at 100 K using φ and ω scans. Crystal-to-detector distance was 60 mm using variable exposure time (3s-5s) with a scan width of 0.500º. Data collection was 99.4% complete to 27.652º in θ (0.7657 Å). A total of 160335 reflections were collected covering the indices, -14 ≤ h ≤ 14, -16 ≤ k ≤ 16, -25 ≤ l ≤ 25. 12128 reflections were found to be symmetry independent with an R_{int} of 0.0327. Indexing and unit cell refinement indicated a primitive, triclinic lattice. The space group was found to be P−1. The data were integrated using the Bruker SAINT software program and scaled using the SADABS software program. Solution by dual-space method (SHELXT) produced a complete phasing model for refinement.

All non-hydrogen atoms were refined anisotropically by full-matrix least-squares (SHELXT-2018). All hydrogens were placed using a riding model. Their positions were constrained relative to their parent atom using the appropriate HFIX command in SHELXL-2019.

Table S15. Crystal data and structure refinement for [**L**Cu^{III}Cl][SbF_6]

Report date	2026-02-10	
Identification code	[**L**Cu^{III}Cl][SbF_6]	
Empirical formula	C18 H16 Cl3 Cu F6 N3 S3 Sb	
Molecular formula	C16 H12 Cl Cu N3 S3, F6 Sb, 1[C2 H4 Cl2]	
Formula weight	776.16	
Temperature	100 K	
Wavelength	0.71073 Å	
Crystal system	Triclinic	
Space group	P-1	
Unit cell dimensions	11.4583(9) Å	α = 99.088(3)º
	12.3181(9) Å	β = 93.739(3)º
	19.5463(16) Å	γ = 104.533(2)º
Volume	2621.1(4) $Å^3$	
Z	2	
Density (calculated)	1.967 g/cm^3	
Absorption coefficient (μ)	2.44 mm^{-1}	
F(000)	1512.0	

Crystal size	0.048 × 0.081 × 0.202 mm^3
Crystal color, habit	Blueish purple block
Theta range for data collection	1.847 to 27.652º
Index ranges	$-14 \leq h \leq 14$, $-16 \leq k \leq 16$, $-25 \leq l \leq 25$
Reflections collected	160335
Independent reflections	12128 [R(int) = 0.0327, R(sigma) = 0.0127]
Completeness to theta = 27.652º	99.4 %
Absorption correction	Multi-scan
Max. and min. transmission	0.0950 and 0.0659
Refinement method	Multi-scan
Data / restraints / parameters	12128 / 0 / 631
Goodness-of-fit on F^2	1.065
Final R indexes [I>2sigma (I)]	R1 = 0.0212, wR2 = 0.0513
Final R indexes [all data]	R1 = 0.0231, wR2 = 0.0524
Largest diff. peak/hole	1.54 and -1.24 e Å^{-3}

Table S16. Fractional Atomic Coordinates (×10^4) and Equivalent Isotropic Displacement Parameters (Å^2×10^3) for [**L**Cu^{III}Cl][SbF$_6$]. U_{eq} is defined as 1/3 of the trace of the orthogonalised U_{IJ} tensor

	x	*y*	*z*	U(eq)
Sb01	2523.9(2)	4656.3(2)	8487.8(2)	17.99(3)
Sb02	6766.1(2)	8743.4(2)	4666.9(2)	24.55(4)
Cu03	-1684.7(2)	5028.3(2)	7081.6(2)	14.33(5)
Cu04	7582.5(2)	10162.5(2)	8441.8(2)	13.94(5)
Cl05	-465.3(4)	6619.7(4)	6827.0(3)	19.12(9)
S006	-3074.5(5)	2549.3(4)	6553.6(3)	20.6(1)
S007	7622.8(5)	8181.4(4)	7306.5(3)	18.52(10)
S008	-2051.4(5)	3242.1(4)	8020.7(3)	19.41(10)
Cl09	8674.6(5)	11604.3(4)	9278.9(3)	22.14(10)

Table S16. Fractional Atomic Coordinates (×10^4) and Equivalent Isotropic Displacement Parameters (Å^2×10^3) for [**L**Cu^{III}Cl][SbF$_6$]. U$_{eq}$ is defined as 1/3 of the trace of the orthogonalised U$_{IJ}$ tensor

	x	*y*	*z*	U(eq)
S00A	5478.0(4)	7975.5(4)	8047.0(3)	18.31(10)
S00B	5957.7(5)	9619.7(4)	7080.3(3)	20.71(10)
S00C	-4142.1(5)	4017.5(5)	7533.7(3)	20.63(10)
Cl0D	2940.3(5)	7086.9(5)	6843.0(3)	29.07(11)
Cl0E	1059.1(7)	9422.6(5)	8604.3(3)	37.69(14)
Cl0F	3778.3(6)	9336.8(5)	9404.3(3)	34.26(13)
Cl0G	1991.7(7)	6126.8(6)	5133.1(3)	38.89(15)
F00H	5293.7(12)	7662.2(13)	4657.2(8)	35.5(3)
F00I	3500.7(14)	5915.7(11)	8186.8(7)	32.8(3)
F00J	7011.7(15)	8013.0(13)	3792.1(8)	38.2(4)
F00K	2787.9(16)	3719.9(12)	7690.7(8)	43.2(4)
F00L	1613.1(15)	3367.0(13)	8795.4(8)	41.9(4)
F00M	3921.2(15)	4465.6(16)	8956.7(10)	48.7(4)
F00N	2310.6(19)	5587.6(14)	9291.7(9)	50.9(5)
N00O	8881.4(15)	10303.9(14)	7772.0(9)	15.5(3)
F00P	5985.0(18)	9628.3(15)	4201.8(10)	52.9(5)
F00Q	8257.4(15)	9824.6(14)	4682.3(9)	51.2(5)
N00R	-1274.8(15)	5468.0(14)	8136.0(9)	16.2(3)
N00S	-882.3(15)	3903.2(14)	6548.4(9)	17.0(3)
N00T	-3240.4(15)	5225.3(14)	6600.7(9)	16.2(3)
N00U	6107.1(15)	10811.0(14)	8342.5(9)	18.6(3)
N00V	7286.1(15)	8919.8(14)	9048.0(9)	16.1(3)
F00W	7571.4(15)	7825.0(18)	5088.6(10)	54.0(5)
F00X	6571.5(17)	9502.4(19)	5530.9(9)	63.0(6)
F00Y	1151.7(15)	4824(2)	8016.8(10)	63.8(6)

Table S16. Fractional Atomic Coordinates (×10^4) and Equivalent Isotropic Displacement Parameters (Å^2×10^3) for [**L**Cu^{III}Cl][SbF_6]. U_{eq} is defined as 1/3 of the trace of the orthogonalised U_{IJ} tensor

	x	*y*	*z*	U(eq)
C00Z	8792.3(18)	9402.0(17)	7262.7(11)	17.7(4)
C010	6587.7(18)	8899.8(16)	7666.7(10)	16.1(4)
C011	-1495.1(18)	4627.3(17)	8513.3(11)	17.8(4)
C012	-1560.7(19)	2819.3(17)	6349.8(11)	19.5(4)
C013	9791.7(18)	11244.7(17)	7782.6(11)	18.6(4)
C014	-2818.6(18)	3608.5(16)	7313.1(10)	16.6(4)
C015	-806.2(18)	6543.3(17)	8485.9(11)	18.6(4)
C016	-4292.0(19)	4722.0(17)	6828.5(10)	18.0(4)
C017	10516(2)	10357.2(19)	6779.1(12)	25.7(5)
C018	6385.8(18)	7964.7(17)	8809.2(10)	17.3(4)
C019	9574(2)	9391.4(18)	6754.3(11)	23.1(4)
C01A	6118(2)	7045.5(18)	9153.9(12)	22.1(4)
C01B	-1151(2)	1967.2(19)	5966.3(12)	25.6(5)
C01C	5443.5(19)	10518.5(17)	7716.3(12)	21.1(4)
C01D	-774(2)	5912(2)	9580.5(11)	24.0(4)
C01E	-525.5(19)	6786.2(19)	9201.9(11)	21.5(4)
C01F	10626.6(19)	11294.8(18)	7300.3(12)	23.2(4)
C01G	-1280.3(19)	4803.5(19)	9234.1(11)	22.4(4)
C01H	-5423(2)	4754.4(19)	6549.5(12)	22.5(4)
C01I	255(2)	4169.3(19)	6372.0(11)	22.6(4)
C01J	6800(2)	7129.1(19)	9781.7(12)	23.4(4)
C01K	-3298(2)	5782.4(17)	6066.1(10)	19.3(4)
C01L	-4390(2)	5868.2(18)	5761.9(11)	23.7(4)
C01M	7944(2)	8978.2(18)	9654.6(11)	20.2(4)
C01N	720(2)	3371(2)	5972.1(13)	28.2(5)

Table S16. Fractional Atomic Coordinates (×10[4]) and Equivalent Isotropic Displacement Parameters (Å[2]×10[3]) for [**L**Cu[III]Cl][SbF6]. U_{eq} is defined as 1/3 of the trace of the orthogonalised U_{IJ} tensor

	x	*y*	*z*	U(eq)
C01O	7718(2)	8108.1(19)	10038.7(11)	23.9(4)
C01P	-5458(2)	5347.1(19)	6003.9(12)	25.3(4)
C01Q	4451(2)	10934(2)	7563.9(15)	30.4(5)
C01R	5(2)	2264(2)	5766.7(12)	28.9(5)
C01S	5780(2)	11515.7(18)	8847.2(13)	25.2(5)
C01T	2466(2)	7878(2)	6236.3(13)	28.6(5)
C01U	4144(2)	11677(2)	8086.4(16)	36.9(6)
C01V	4806(2)	11966(2)	8733.0(15)	33.3(6)
C01W	2626(2)	8178(2)	8894.5(18)	40.9(7)
C01X	2825(2)	7554(2)	5528.3(13)	32.7(5)
C01Y	2003(3)	8532(2)	8309.6(14)	40.0(7)

Table S17. Anisotropic Displacement Parameters (Å[2]×10[3]) for [**L**Cu[III]Cl][SbF6]. The Anisotropic displacement factor exponent takes the form: $-2\pi^2[h^2a^{*2}U_{11}+2hka^*b^*U_{12}+\ldots]$

	U_{11}	U_{22}	U_{33}	U_{23}	U_{13}	U_{12}
Sb01	17.91(7)	16.88(7)	18.84(7)	5.68(5)	3.80(5)	2.02(5)
Sb02	24.04(7)	25.54(8)	20.83(7)	3.27(5)	8.55(6)	-0.28(6)
Cu03	15.34(11)	11.32(10)	16.23(11)	3.58(8)	2.46(9)	2.51(8)
Cu04	14.73(11)	11.92(11)	13.61(11)	1.01(8)	1.82(8)	1.44(8)
Cl05	19.2(2)	14.6(2)	23.3(2)	5.50(17)	5.83(18)	1.96(17)
S006	21.3(2)	14.0(2)	23.2(2)	2.26(18)	-2.43(19)	0.58(18)
S007	22.9(2)	12.1(2)	18.8(2)	0.86(17)	6.22(19)	1.42(18)
S008	22.6(2)	15.6(2)	20.9(2)	7.13(18)	0.09(19)	4.88(18)
Cl09	27.2(3)	17.2(2)	16.9(2)	-1.18(17)	-0.11(18)	-0.32(18)

Table S17. Anisotropic Displacement Parameters (Å^2×10^3) for [$\mathbf{L}Cu^{III}Cl$][SbF_6]. The Anisotropic displacement factor exponent takes the form: $-2\pi^2[h^2a^{*2}U_{11}+2hka^*b^*U_{12}+\ldots]$

	U_{11}	U_{22}	U_{33}	U_{23}	U_{13}	U_{12}
S00A	15.2(2)	17.2(2)	20.8(2)	7.24(18)	0.73(18)	-0.83(17)
S00B	22.8(2)	19.2(2)	18.3(2)	6.87(18)	-1.99(19)	1.01(19)
S00C	17.2(2)	23.8(2)	23.3(2)	11.0(2)	4.47(19)	4.99(19)
Cl0D	29.4(3)	36.5(3)	21.1(2)	3.6(2)	0.6(2)	10.0(2)
Cl0E	52.1(4)	25.5(3)	32.1(3)	2.6(2)	-12.7(3)	10.2(3)
Cl0F	30.3(3)	34.9(3)	41.2(3)	13.7(3)	11.1(2)	9.1(2)
Cl0G	55.5(4)	34.1(3)	26.9(3)	0.2(2)	-12.7(3)	19.3(3)
F00H	21.8(7)	40.6(8)	43.4(9)	20.8(7)	1.6(6)	-1.0(6)
F00I	46.0(9)	18.8(6)	31.9(7)	11.1(5)	10.4(6)	0.1(6)
F00J	45.8(9)	30.7(8)	30.7(8)	-6.9(6)	13.5(7)	2.1(7)
F00K	61.8(11)	24.1(7)	37.1(8)	-1.9(6)	25.0(8)	-1.1(7)
F00L	48.2(9)	31.7(8)	32.3(8)	8.0(6)	9.6(7)	-16.9(7)
F00M	32.8(8)	61.2(11)	59.4(11)	37.7(9)	-4.2(8)	11.3(8)
F00N	89.3(14)	31.0(8)	33.6(9)	2.9(7)	31.4(9)	13.7(9)
N00O	15.7(8)	13.6(7)	17.3(8)	3.6(6)	2.3(6)	3.3(6)
F00P	68.3(12)	40.3(9)	66.4(12)	30.5(9)	26.2(10)	26.6(9)
F00Q	42.4(9)	43.0(9)	46.0(10)	-17.7(7)	27.2(8)	-18.9(7)
N00R	15.6(8)	16.5(8)	16.7(8)	3.2(6)	2.2(6)	4.6(6)
N00S	19.0(8)	16.2(8)	15.8(8)	2.4(6)	-0.5(6)	5.4(6)
N00T	18.5(8)	12.7(7)	16.8(8)	2.4(6)	0.9(6)	3.4(6)
N00U	17.5(8)	15.3(8)	25.0(9)	8.2(7)	6.3(7)	4.0(6)
N00V	19.1(8)	14.3(8)	15.6(8)	3.4(6)	4.1(6)	4.7(6)
F00W	29.4(8)	84.7(14)	58.1(11)	38.2(10)	6.5(8)	17.9(9)
F00X	50.5(11)	82.7(14)	35.3(9)	-21.1(9)	25.1(8)	-5.3(10)
F00Y	27.9(9)	125.6(19)	54.6(11)	43.7(12)	7.1(8)	33.4(10)

Table S17. Anisotropic Displacement Parameters (Å^2×10^3) for [**L**Cu^{III}Cl][SbF$_6$]. The Anisotropic displacement factor exponent takes the form: $-2\pi^2[h^2a^{*2}U_{11}+2hka^*b^*U_{12}+\ldots]$

	U_{11}	U_{22}	U_{33}	U_{23}	U_{13}	U_{12}
C00Z	19.7(10)	14.1(9)	20.0(9)	4.9(7)	4.7(8)	3.8(7)
C010	16.3(9)	13.4(9)	15.8(9)	2.2(7)	0.6(7)	-0.3(7)
C011	15.6(9)	18.4(9)	20.5(10)	4.8(7)	2.0(7)	5.8(7)
C012	22.8(10)	17.9(9)	17.9(9)	2.8(7)	-1.1(8)	6.8(8)
C013	17.5(9)	14.3(9)	23.6(10)	4.8(7)	0.8(8)	2.8(7)
C014	16.7(9)	13.2(9)	19.6(9)	5.6(7)	0.8(7)	2.0(7)
C015	16.8(9)	17.3(9)	21.1(10)	2.1(7)	2.6(7)	4.3(7)
C016	21.1(10)	15.5(9)	17.8(9)	3.5(7)	1.3(7)	5.7(7)
C017	26.5(11)	25.7(11)	30.9(12)	12.7(9)	15.5(9)	10.2(9)
C018	16.4(9)	18.8(9)	17.5(9)	5.8(7)	3.8(7)	4.4(7)
C019	31.3(12)	18.5(10)	23.2(10)	5.6(8)	12.0(9)	10.0(9)
C01A	21.6(10)	20.2(10)	25.9(11)	9.5(8)	6.2(8)	4.2(8)
C01B	36.1(12)	17.7(10)	22.6(11)	0.5(8)	-2.2(9)	9.7(9)
C01C	18.3(10)	16.6(9)	29.4(11)	11.2(8)	4.1(8)	2.1(8)
C01D	22.1(10)	34.1(12)	16.3(10)	2.2(8)	1.3(8)	10.1(9)
C01E	18.5(10)	23.1(10)	20.7(10)	-2.0(8)	0.6(8)	5.4(8)
C01F	18.2(10)	19.6(10)	33.2(12)	10.8(9)	6.4(8)	2.7(8)
C01G	21.5(10)	28.7(11)	19.9(10)	8.8(8)	2.3(8)	9.2(8)
C01H	18.9(10)	24.1(10)	25.0(11)	3.1(8)	2.2(8)	7.3(8)
C01I	21.7(10)	22.4(10)	23.9(10)	2.5(8)	2.5(8)	7.5(8)
C01J	28.2(11)	23.3(10)	24.1(11)	12.1(8)	8.9(9)	10.1(9)
C01K	26.1(10)	14.6(9)	15.9(9)	1.9(7)	0.9(8)	4.1(8)
C01L	32.0(12)	20.4(10)	18.9(10)	3.6(8)	-3.3(8)	9.0(9)
C01M	24.0(10)	17.6(9)	18.7(10)	1.0(7)	1.2(8)	6.5(8)
C01N	27.2(12)	31.6(12)	29.5(12)	3.7(9)	7.5(9)	14.6(10)

Table S17. Anisotropic Displacement Parameters (Å^2×10^3) for [**L**Cu^{III}Cl][SbF$_6$]. The Anisotropic displacement factor exponent takes the form: $-2\pi^2[h^2a^{*2}U_{11}+2hka^*b^*U_{12}+\ldots]$

	U_{11}	U_{22}	U_{33}	U_{23}	U_{13}	U_{12}
C01O	32.2(12)	24.9(11)	17.6(10)	4.7(8)	1.6(8)	12.7(9)
C01P	24.6(11)	25.7(11)	26.4(11)	2.7(9)	-5.1(9)	11.5(9)
C01Q	20.3(11)	25.0(11)	48.3(15)	18.3(10)	-0.8(10)	4.1(9)
C01R	38.2(13)	27.5(12)	25.5(11)	-0.1(9)	3.6(10)	20.1(10)
C01S	29.6(11)	19.6(10)	30.8(12)	10.0(9)	15.5(9)	8.2(9)
C01T	29.9(12)	25.3(11)	30.9(12)	6.0(9)	2.9(9)	7.5(9)
C01U	24.9(12)	28.2(12)	67.7(19)	24.6(12)	13.1(12)	13.5(10)
C01V	34.8(13)	25.1(12)	50.0(16)	15.6(11)	24.1(12)	15.8(10)
C01W	28.9(13)	17.6(11)	78(2)	8.2(12)	24.0(13)	5.5(9)
C01X	35.7(13)	34.9(13)	26.8(12)	8.2(10)	2.2(10)	7.0(10)
C01Y	39.2(14)	32.0(13)	35.9(14)	-11.0(11)	17.7(11)	-7.1(11)

Table S18. Bond Lengths for [**L**Cu^{III}Cl][SbF$_6$].

		Length/Å			Length/Å
Sb01	F00I	1.8731(13)	N00O	C00Z	1.348(3)
Sb01	F00K	1.8759(15)	N00O	C013	1.347(3)
Sb01	F00L	1.8774(14)	N00R	C011	1.347(3)
Sb01	F00M	1.8773(15)	N00R	C015	1.349(3)
Sb01	F00N	1.8617(15)	N00S	C012	1.347(3)
Sb01	F00Y	1.8431(16)	N00S	C01I	1.343(3)
Sb02	F00H	1.8656(14)	N00T	C016	1.348(3)
Sb02	F00J	1.8752(14)	N00T	C01K	1.345(3)
Sb02	F00P	1.8741(17)	N00U	C01C	1.345(3)
Sb02	F00Q	1.8797(15)	N00U	C01S	1.344(3)

Table S18. Bond Lengths for [**L**Cu^{III}Cl][SbF_6].

		Length/Å			Length/Å
Sb02	F00W	1.8787(17)	N00V	C018	1.349(3)
Sb02	F00X	1.8523(16)	N00V	C01M	1.345(3)
Cu03	Cl05	2.2511(5)	C00Z	C019	1.381(3)
Cu03	N00R	2.0384(17)	C011	C01G	1.387(3)
Cu03	N00S	2.0397(17)	C012	C01B	1.389(3)
Cu03	N00T	2.0471(17)	C013	C01F	1.383(3)
Cu03	C014	2.0341(19)	C015	C01E	1.383(3)
Cu04	Cl09	2.2577(5)	C016	C01H	1.385(3)
Cu04	N00O	2.0385(17)	C017	C019	1.383(3)
Cu04	N00U	2.0536(17)	C017	C01F	1.388(3)
Cu04	N00V	2.0561(16)	C018	C01A	1.387(3)
Cu04	C010	2.0382(19)	C01A	C01J	1.386(3)
S006	C012	1.766(2)	C01B	C01R	1.382(4)
S006	C014	1.770(2)	C01C	C01Q	1.393(3)
S007	C00Z	1.763(2)	C01D	C01E	1.383(3)
S007	C010	1.768(2)	C01D	C01G	1.385(3)
S008	C011	1.761(2)	C01H	C01P	1.388(3)
S008	C014	1.768(2)	C01I	C01N	1.391(3)
S00A	C010	1.769(2)	C01J	C01O	1.384(3)
S00A	C018	1.764(2)	C01K	C01L	1.385(3)
S00B	C010	1.776(2)	C01L	C01P	1.382(3)
S00B	C01C	1.763(2)	C01M	C01O	1.386(3)
S00C	C014	1.772(2)	C01N	C01R	1.384(4)
S00C	C016	1.762(2)	C01Q	C01U	1.385(4)
Cl0D	C01T	1.789(2)	C01S	C01V	1.386(3)

Table S18. Bond Lengths for [**L**Cu^{III}Cl][SbF_6].

		Length/Å			Length/Å
Cl0E	C01Y	1.784(3)	C01T	C01X	1.492(3)
Cl0F	C01W	1.791(3)	C01U	C01V	1.377(4)
Cl0G	C01X	1.798(3)	C01W	C01Y	1.488(4)

Table S19. Bond Angles for [**L**Cu^{III}Cl][SbF_6].

			Angle/°				Angle/°
F00I	Sb01	F00K	88.82(6)	C01K	N00T	Cu03	125.40(14)
F00I	Sb01	F00L	177.16(8)	C01K	N00T	C016	117.77(18)
F00I	Sb01	F00M	88.71(7)	C01C	N00U	Cu04	116.53(14)
F00K	Sb01	F00L	90.02(7)	C01S	N00U	Cu04	125.02(16)
F00K	Sb01	F00M	88.98(9)	C01S	N00U	C01C	118.43(19)
F00M	Sb01	F00L	88.67(8)	C018	N00V	Cu04	117.44(13)
F00N	Sb01	F00I	90.79(7)	C01M	N00V	Cu04	124.75(14)
F00N	Sb01	F00K	178.19(9)	C01M	N00V	C018	117.79(17)
F00N	Sb01	F00L	90.29(7)	N00O	C00Z	S007	114.94(15)
F00N	Sb01	F00M	89.24(9)	N00O	C00Z	C019	123.85(19)
F00Y	Sb01	F00I	91.52(8)	C019	C00Z	S007	121.16(16)
F00Y	Sb01	F00K	90.15(9)	S007	C010	Cu04	105.80(10)
F00Y	Sb01	F00L	91.09(9)	S007	C010	S00A	110.71(10)
F00Y	Sb01	F00M	179.10(10)	S007	C010	S00B	113.74(11)
F00Y	Sb01	F00N	91.63(10)	S00A	C010	Cu04	107.96(10)
F00H	Sb02	F00J	91.33(7)	S00A	C010	S00B	113.19(11)
F00H	Sb02	F00P	90.84(8)	S00B	C010	Cu04	104.82(9)
F00H	Sb02	F00Q	179.42(9)	N00R	C011	S008	114.94(15)

Table S19. Bond Angles for [**L**Cu^{III}Cl][SbF_6].

			Angle/°				Angle/°
F00H	Sb02	F00W	89.58(7)	N00R	C011	C01G	124.05(19)
F00J	Sb02	F00Q	88.75(7)	C01G	C011	S008	120.98(16)
F00J	Sb02	F00W	89.10(8)	N00S	C012	S006	115.70(15)
F00P	Sb02	F00J	87.96(8)	N00S	C012	C01B	123.5(2)
F00P	Sb02	F00Q	89.74(9)	C01B	C012	S006	120.68(17)
F00P	Sb02	F00W	177.04(9)	N00O	C013	C01F	122.12(19)
F00W	Sb02	F00Q	89.84(9)	S006	C014	Cu03	106.18(10)
F00X	Sb02	F00H	90.60(8)	S006	C014	S00C	113.37(11)
F00X	Sb02	F00J	178.07(8)	S008	C014	Cu03	106.43(10)
F00X	Sb02	F00P	91.93(10)	S008	C014	S006	112.09(11)
F00X	Sb02	F00Q	89.32(8)	S008	C014	S00C	112.80(11)
F00X	Sb02	F00W	91.00(10)	S00C	C014	Cu03	105.24(9)
N00R	Cu03	Cl05	96.21(5)	N00R	C015	C01E	122.06(19)
N00R	Cu03	N00S	118.44(7)	N00T	C016	S00C	115.12(15)
N00R	Cu03	N00T	121.17(7)	N00T	C016	C01H	123.97(19)
N00S	Cu03	Cl05	96.77(5)	C01H	C016	S00C	120.90(16)
N00S	Cu03	N00T	117.10(7)	C019	C017	C01F	119.2(2)
N00T	Cu03	Cl05	95.20(5)	N00V	C018	S00A	115.94(14)
C014	Cu03	Cl05	178.65(6)	N00V	C018	C01A	123.32(19)
C014	Cu03	N00R	83.78(7)	C01A	C018	S00A	120.61(16)
C014	Cu03	N00S	84.40(8)	C00Z	C019	C017	117.9(2)
C014	Cu03	N00T	83.67(7)	C01J	C01A	C018	117.9(2)
N00O	Cu04	Cl09	95.21(5)	C01R	C01B	C012	117.7(2)
N00O	Cu04	N00U	123.37(7)	N00U	C01C	S00B	115.36(15)
N00O	Cu04	N00V	122.14(7)	N00U	C01C	C01Q	122.9(2)

Table S19. Bond Angles for [**L**Cu^{III}Cl][SbF_6].

			Angle/°				Angle/°
N00U	Cu04	Cl09	95.79(5)	C01Q	C01C	S00B	121.72(19)
N00U	Cu04	N00V	111.24(7)	C01E	C01D	C01G	119.4(2)
N00V	Cu04	Cl09	97.05(5)	C015	C01E	C01D	119.6(2)
C010	Cu04	Cl09	178.09(6)	C013	C01F	C017	119.4(2)
C010	Cu04	N00O	84.25(7)	C01D	C01G	C011	117.5(2)
C010	Cu04	N00U	83.01(8)	C016	C01H	C01P	117.2(2)
C010	Cu04	N00V	84.77(7)	N00S	C01I	C01N	121.8(2)
C012	S006	C014	97.30(10)	C01O	C01J	C01A	119.6(2)
C00Z	S007	C010	97.24(9)	N00T	C01K	C01L	122.0(2)
C011	S008	C014	97.40(9)	C01P	C01L	C01K	119.2(2)
C018	S00A	C010	98.02(9)	N00V	C01M	C01O	122.5(2)
C01C	S00B	C010	96.50(10)	C01R	C01N	C01I	119.2(2)
C016	S00C	C014	96.88(10)	C01J	C01O	C01M	118.9(2)
C00Z	N00O	Cu04	117.37(13)	C01L	C01P	C01H	119.8(2)
C013	N00O	Cu04	125.00(14)	C01U	C01Q	C01C	117.8(2)
C013	N00O	C00Z	117.57(17)	C01B	C01R	C01N	119.6(2)
C011	N00R	Cu03	117.83(14)	N00U	C01S	C01V	121.9(2)
C011	N00R	C015	117.42(17)	C01X	C01T	Cl0D	111.89(17)
C015	N00R	Cu03	124.75(14)	C01V	C01U	C01Q	119.7(2)
C012	N00S	Cu03	116.92(14)	C01U	C01V	C01S	119.3(2)
C01I	N00S	Cu03	124.92(14)	C01Y	C01W	Cl0F	112.40(18)
C01I	N00S	C012	118.14(18)	C01T	C01X	Cl0G	111.11(18)
C016	N00T	Cu03	116.80(13)	C01W	C01Y	Cl0E	112.40(18)

Table S20. Torsion Angles for [$\mathbf{L}Cu^{III}Cl$][SbF_6].

				Angle/°					Angle/°
Cu03	N00R	C011	S008	2.8(2)	C011	S008	C014	S006	159.34(11)
Cu03	N00R	C011	C01G	-178.89(16)	C011	S008	C014	S00C	-71.25(12)
Cu03	N00R	C015	C01E	-178.76(15)	C011	N00R	C015	C01E	1.8(3)
Cu03	N00S	C012	S006	2.4(2)	C012	S006	C014	Cu03	43.28(11)
Cu03	N00S	C012	C01B	178.04(16)	C012	S006	C014	S008	-72.54(12)
Cu03	N00S	C01I	C01N	-176.17(17)	C012	S006	C014	S00C	158.34(11)
Cu03	N00T	C016	S00C	2.4(2)	C012	N00S	C01I	C01N	2.5(3)
Cu03	N00T	C016	C01H	-178.65(16)	C012	C01B	C01R	C01N	2.2(3)
Cu03	N00T	C01K	C01L	179.22(15)	C013	N00O	C00Z	S007	177.21(14)
Cu04	N00O	C00Z	S007	-5.4(2)	C013	N00O	C00Z	C019	-0.2(3)
Cu04	N00O	C00Z	C019	177.16(17)	C014	S006	C012	N00S	-29.77(17)
Cu04	N00O	C013	C01F	-178.22(15)	C014	S006	C012	C01B	154.51(18)
Cu04	N00U	C01C	S00B	-0.03(19)	C014	S008	C011	N00R	-30.05(17)
Cu04	N00U	C01C	C01Q	-177.06(16)	C014	S008	C011	C01G	151.60(18)
Cu04	N00U	C01S	C01V	176.75(16)	C014	S00C	C016	N00T	-31.53(17)
Cu04	N00V	C018	S00A	-6.3(2)	C014	S00C	C016	C01H	149.48(18)
Cu04	N00V	C018	C01A	177.68(16)	C015	N00R	C011	S008	-177.68(14)
Cu04	N00V	C01M	C01O	-178.98(16)	C015	N00R	C011	C01G	0.6(3)
S006	C012	C01B	C01R	173.78(17)	C016	S00C	C014	Cu03	46.15(11)
S007	C00Z	C019	C017	-176.00(17)	C016	S00C	C014	S006	-69.47(12)
S008	C011	C01G	C01D	176.19(16)	C016	S00C	C014	S008	161.78(11)
S00A	C018	C01A	C01J	-174.56(16)	C016	N00T	C01K	C01L	1.4(3)
S00B	C01C	C01Q	C01U	-177.11(17)	C016	C01H	C01P	C01L	0.2(3)
S00C	C016	C01H	C01P	178.73(16)	C018	S00A	C010	Cu04	-39.91(11)
Cl0D	C01T	C01X	Cl0G	68.3(2)	C018	S00A	C010	S007	75.47(12)

Table S20. Torsion Angles for [**L**Cu^{III}Cl][SbF$_6$].

				Angle/°					Angle/°
Cl0F	C01W	C01Y	Cl0E	-70.4(2)	C018	S00A	C010	S00B	-155.47(11)
N00O	C00Z	C019	C017	1.3(3)	C018	N00V	C01M	C01O	-0.8(3)
N00O	C013	C01F	C017	1.2(3)	C018	C01A	C01J	C01O	-0.4(3)
N00R	C011	C01G	C01D	-2.0(3)	C019	C017	C01F	C013	-0.1(3)
N00R	C015	C01E	C01D	-2.7(3)	C01A	C01J	C01O	C01M	-0.9(3)
N00S	C012	C01B	C01R	-1.6(3)	C01C	S00B	C010	Cu04	-47.15(11)
N00S	C01I	C01N	C01R	-1.9(3)	C01C	S00B	C010	S007	-162.25(11)
N00T	C016	C01H	C01P	-0.2(3)	C01C	S00B	C010	S00A	70.26(12)
N00T	C01K	C01L	C01P	-1.4(3)	C01C	N00U	C01S	C01V	-1.7(3)
N00U	C01C	C01Q	C01U	-0.3(3)	C01C	C01Q	C01U	C01V	-0.8(3)
N00U	C01S	C01V	C01U	0.6(3)	C01E	C01D	C01G	C011	1.0(3)
N00V	C018	C01A	C01J	1.2(3)	C01F	C017	C019	C00Z	-1.1(3)
N00V	C01M	C01O	C01J	1.6(3)	C01G	C01D	C01E	C015	1.2(3)
C00Z	S007	C010	Cu04	-44.61(11)	C01I	N00S	C012	S006	-176.35(15)
C00Z	S007	C010	S00A	-161.33(11)	C01I	N00S	C012	C01B	-0.8(3)
C00Z	S007	C010	S00B	69.90(12)	C01I	C01N	C01R	C01B	-0.5(4)
C00Z	N00O	C013	C01F	-1.1(3)	C01K	N00T	C016	S00C	-179.59(14)
C010	S007	C00Z	N00O	32.50(17)	C01K	N00T	C016	C01H	-0.6(3)
C010	S007	C00Z	C019	-150.00(18)	C01K	C01L	C01P	C01H	0.5(3)
C010	S00A	C018	N00V	29.76(17)	C01M	N00V	C018	S00A	175.35(15)
C010	S00A	C018	C01A	-154.15(18)	C01M	N00V	C018	C01A	-0.6(3)
C010	S00B	C01C	N00U	30.70(17)	C01Q	C01U	C01V	C01S	0.7(4)
C010	S00B	C01C	C01Q	-152.23(18)	C01S	N00U	C01C	S00B	178.55(15)
C011	S008	C014	Cu03	43.67(11)	C01S	N00U	C01C	C01Q	1.5(3)

Table S21. Hydrogen Atom Coordinates (Å×10^4) and Isotropic Displacement Parameters (Å^2×10^3) for [**L**Cu^{III}Cl][SbF_6].

	x	*y*	*z*	U(eq)
H013	9862.7	11894.78	8132.44	22
H015	-664.64	7152.97	8232.44	22
H017	11081.31	10378.47	6443.38	31
H019	9468.36	8741.6	6398.92	28
H01A	5487.06	6379.9	8965.88	26
H01B	-1648.1	1206.78	5845.31	31
H01D	-599.16	6070.25	10073.49	29
H01E	-164.31	7548.27	9432.3	26
H01F	11269.1	11964.53	7325.72	28
H01G	-1473.07	4187.42	9480.74	27
H01H	-6144.07	4386.56	6724.2	27
H01I	755.48	4922.89	6524.56	27
H01J	6638.81	6518.3	10033.89	28
H01K	-2567.73	6127.79	5891.47	23
H01L	-4404.68	6280.65	5391.2	28
H01M	8587.34	9641.2	9826.27	24
H01N	1518.9	3583.8	5841.49	34
H01O	8185.49	8182.25	10471.34	29
H01P	-6212.95	5394.36	5797.53	30
H01Q	4000.41	10715.57	7116.09	37
H01R	307.26	1711.12	5490.13	35
H01S	6230.33	11710.7	9295.06	30
H01C	1573.33	7740.23	6205.83	34
H01T	2832.7	8702.94	6407.57	34
H01U	3479.09	11985.75	7999.33	44

Table S21. Hydrogen Atom Coordinates (Å×10^4) and Isotropic Displacement Parameters (Å^2×10^3) for [**L**Cu^{III}Cl][SbF_6].

	x	*y*	*z*	U(eq)
H01V	4597.69	12468.38	9096.61	40
H01Y	2996.87	7566.56	8703.54	49
H	2019.04	7862.52	9199.02	49
H01W	3704.11	7609.47	5564.7	39
H01X	2668.85	8092.84	5229.77	39
H01Z	1501.6	7842.93	7990.46	48
HA	2619.27	8945.81	8043.85	48

[**L**Cu^{III}Br][SbF_6]

The single crystal X-ray diffraction studies were carried out on a Bruker Kappa Photon III CPAD diffractometer equipped with Mo K_α radiation (λ = 0.71073 Å). A 0.246 × 0.038 × 0.03 mm deep purple needle was mounted on a Cryoloop with Fomblin oil. Data were collected in a nitrogen gas stream at 100 K using φ and ω scans. Crystal-to-detector distance was 60 mm using variable exposure time (2-5s) depending on θ with a scan width of 0.500º. Data collection was 99.9% complete to 25.392º in θ (0.8287 Å). A total of 104923 reflections were collected covering the indices, $-13 \leq h \leq 13$, $-28 \leq k \leq 28$, $-14 \leq l \leq 14$. 5790 reflections were found to be symmetry independent with an R_{int} of 0.0741. Indexing and unit cell refinement indicated a primitive, monoclinic lattice. The space group was found to be $P2_1/c$. The data were integrated using the Bruker SAINT software program and scaled using the SADABS software program. Solution by dual-space method (SHELXT) produced a complete phasing model for refinement.

All non-hydrogen atoms were refined anisotropically by full-matrix least-squares (SHELXT-2018). All hydrogens were placed using a riding model. Their positions were constrained relative to their parent atom using the appropriate HFIX command in SHELXL-2019. Due to unmodelable solvent disorder, an OLEX2 solvent mask was used to remove the electron density contribution from the disordered solvent from the lattice. Solvent appeared to be DCM, which is consistent with modeled solvent in the lattice. 1 void was found with approximately 162 electrons equating to approximately 1 DCM in the void.

Table S22. Crystal data and structure refinement for [**L**Cu^{III}Br][SbF_6].

Report date	2026-02-10	
Identification code	[**L**Cu^{III}Br][SbF_6]	
Empirical formula	C19 H18 Br Cl6 Cu F6 N3 S3 Sb	
Molecular formula	F6 Sb, C16 H12 Br Cu N3 S3, 2[C H2 Cl2]	
Formula weight	976.44	
Temperature	100 K	
Wavelength	0.71073 Å	
Crystal system	Monoclinic	
Space group	$P2_1/c$	
Unit cell dimensions	a = 11.2630(7)	α = 90°
	b = 23.3253(13)	β = 100.394(2)°
	c = 12.1709(6)	γ = 90°
Volume	3145.0(3) $Å^3$	
Z	4	
Density (calculated)	2.062 g/cm^3	
Absorption coefficient (μ)	3.569 mm^{-1}	
F(000)	1888.0	
Crystal size	0.246 × 0.038 × 0.03 mm^3	
Crystal color, habit	Blueish purple needle	
Theta range for data collection	1.838 to 25.392°	
Index ranges	$-13 \leq h \leq 13$, $-28 \leq k \leq 28$, $-14 \leq l \leq 14$	
Reflections collected	104923	
Independent reflections	5790 [R(int) = 0.0741, R(sigma) = 0.0284]	
Completeness to theta = 25.392°	99.9 %	
Absorption correction	Multi-scan	
Max. and min. transmission	0.0916 and 0.0639	

Refinement method	Full-matrix least-squares on F^2
Data / restraints / parameters	5790 / 0 / 334
Goodness-of-fit on F^2	1.089
Final R indexes [I>2sigma (I)]	R1 = 0.0396, wR2 = 0.0816
Final R indexes [all data]	R_1 = 0.0496, wR_2 = 0.0850
Largest diff. peak and hole	0.66 and -0.53 e Å^{-3}

Table S23. Fractional Atomic Coordinates (×10^4) and Equivalent Isotropic Displacement Parameters (Å^2×10^3) for [**L**Cu^{III}Br][SbF_6]. U_{eq} is defined as 1/3 of the trace of the orthogonalised U_{IJ} tensor.

	x	*y*	*z*	U(eq)
Sb01	13286.5(3)	9164.1(2)	10488.9(3)	24.92(10)
Br02	7635.1(5)	6299.1(2)	10297.0(4)	27.75(13)
Cu03	8898.3(5)	6861.6(3)	9311.4(5)	22.25(14)
S004	9003.2(12)	7785.2(6)	7556.2(10)	25.6(3)
S005	10781.8(11)	6846.1(6)	7813.9(10)	25.5(3)
S006	10914.7(11)	7737.5(6)	9553.0(11)	27.2(3)
Cl07	10549.3(13)	4604.6(6)	6877.2(12)	38.4(3)
Cl08	5405.4(13)	7265.1(7)	7337.4(12)	41.4(4)
Cl09	4013.2(14)	7216.5(7)	9124.7(14)	48.1(4)
Cl0A	10620.6(16)	5240.9(7)	8936.6(13)	47.4(4)
F00C	12656(3)	8907.3(15)	11736(3)	40.8(8)
F00E	14852(3)	9116.8(15)	11321(3)	40.8(8)
F00F	11697(3)	9196.4(15)	9681(3)	41.1(8)
F00G	13153(3)	9924.6(13)	10937(3)	41.9(8)
F00H	13401(3)	8398.5(14)	10067(3)	47.3(9)
F00I	13869(3)	9400.4(18)	9221(3)	57.3(11)
N00J	10455(4)	6749.3(19)	10466(3)	24.4(9)

Table S23. Fractional Atomic Coordinates (×10^4) and Equivalent Isotropic Displacement Parameters (Å^2×10^3) for [**L**Cu^{III}Br][SbF_6]. U_{eq} is defined as 1/3 of the trace of the orthogonalised U_{IJ} tensor.

	x	*y*	*z*	U(eq)
N00K	7903(4)	7602.0(18)	9264(3)	22.1(9)
N00L	8673(4)	6366.4(18)	7896(3)	23.1(9)
C00M	9545(4)	6402(2)	7269(4)	23.0(11)
C00N	8008(4)	7985(2)	8461(4)	23.9(11)
C00O	7121(4)	7739(2)	9942(4)	22.2(10)
C00P	7741(4)	6013(2)	7539(4)	26.8(11)
C00Q	11301(5)	7167(2)	10513(4)	27.8(12)
C00R	9543(5)	6083(2)	6313(4)	25.2(11)
C00S	9974(4)	7339(2)	8497(4)	24.5(11)
C00T	6430(5)	8229(2)	9823(4)	27.6(11)
C00U	7676(5)	5671(2)	6594(4)	28.6(12)
C00V	10705(5)	6311(2)	11169(4)	30.6(12)
C00W	7354(5)	8489(2)	8288(4)	27.2(11)
C00X	6536(5)	8608(2)	8978(4)	28.0(11)
C00Y	11740(5)	6279(3)	11969(4)	35.7(13)
C00Z	8590(5)	5706(2)	5974(4)	27.7(11)
C010	12364(5)	7175(3)	11273(4)	31.8(13)
C011	12582(5)	6715(3)	12026(5)	36.1(14)
C012	11467(5)	5001(3)	7937(5)	36.7(13)
C013	4874(5)	6818(3)	8321(5)	36.1(13)

Table S24. Anisotropic Displacement Parameters (Å^2×10^3) for [**L**Cu^{III}Br][SbF$_6$]. The Anisotropic displacement factor exponent takes the form: $-2\pi^2[h^2a^{*2}U_{11}+2hka^*b^*U_{12}+\ldots]$.

	U_{11}	U_{22}	U_{33}	U_{23}	U_{13}	U_{12}
Sb01	21.86(17)	26.90(19)	27.05(18)	2.90(15)	7.19(13)	4.14(15)
Br02	29.4(3)	31.9(3)	23.3(3)	5.4(2)	8.6(2)	-0.3(2)
Cu03	23.0(3)	26.6(3)	18.0(3)	0.6(2)	6.2(2)	-0.8(3)
S004	27.5(7)	29.6(7)	21.2(6)	3.2(5)	8.2(5)	1.6(6)
S005	22.9(6)	32.0(7)	23.1(6)	-3.2(5)	8.2(5)	-0.8(5)
S006	25.6(6)	29.2(7)	27.0(7)	-3.6(5)	5.5(5)	-3.4(5)
Cl07	37.8(8)	39.9(8)	38.2(8)	2.4(6)	8.8(6)	5.0(6)
Cl08	31.4(7)	55.7(10)	37.2(8)	-15.3(7)	6.6(6)	-6.4(7)
Cl09	38.1(8)	57.1(10)	54.0(9)	-16.1(8)	21.4(7)	-0.9(7)
Cl0A	66.1(11)	43.0(9)	36.7(8)	2.2(7)	18.7(7)	10.0(8)
F00C	37.0(19)	48(2)	40.1(19)	13.7(16)	15.4(15)	5.0(16)
F00E	23.3(16)	42(2)	56(2)	5.1(16)	3.0(15)	4.0(15)
F00F	30.4(17)	53(2)	37.2(18)	2.7(16)	-2.5(14)	11.3(16)
F00G	43.3(19)	26.8(17)	55(2)	-3.5(15)	8.2(16)	7.2(15)
F00H	42(2)	29.2(18)	69(2)	-12.7(17)	6.0(18)	9.2(15)
F00I	58(2)	72(3)	51(2)	22(2)	32.8(19)	15(2)
N00J	26(2)	32(2)	18(2)	-0.9(18)	8.9(17)	0.5(19)
N00K	22(2)	25(2)	18(2)	0.9(17)	2.8(16)	0.7(18)
N00L	25(2)	22(2)	22(2)	-4.2(17)	4.4(17)	-0.7(18)
C00M	25(3)	26(3)	17(2)	6(2)	3(2)	3(2)
C00N	23(3)	28(3)	21(2)	-6(2)	3(2)	-7(2)
C00O	23(2)	26(3)	17(2)	-1(2)	2.4(19)	-4(2)
C00P	20(3)	30(3)	31(3)	3(2)	8(2)	4(2)
C00Q	27(3)	35(3)	23(3)	-4(2)	7(2)	5(2)
C00R	27(3)	28(3)	21(3)	1(2)	6(2)	6(2)

Table S24. Anisotropic Displacement Parameters ($Å^2 \times 10^3$) for [**L**Cu^{III}Br][SbF_6]. The Anisotropic displacement factor exponent takes the form: $-2\pi^2[h^2a^{*2}U_{11}+2hka^*b^*U_{12}+\ldots]$.

	U_{11}	U_{22}	U_{33}	U_{23}	U_{13}	U_{12}
C00S	21(2)	29(3)	23(3)	1(2)	5(2)	-3(2)
C00T	23(3)	30(3)	31(3)	-4(2)	7(2)	2(2)
C00U	24(3)	27(3)	34(3)	-3(2)	2(2)	1(2)
C00V	38(3)	31(3)	24(3)	0(2)	8(2)	7(2)
C00W	29(3)	29(3)	24(3)	0(2)	7(2)	-7(2)
C00X	28(3)	26(3)	31(3)	-2(2)	9(2)	2(2)
C00Y	36(3)	50(4)	20(3)	6(2)	3(2)	10(3)
C00Z	32(3)	25(3)	26(3)	-4(2)	3(2)	3(2)
C010	25(3)	46(4)	23(3)	-7(2)	2(2)	4(2)
C011	31(3)	51(4)	25(3)	-5(3)	-1(2)	8(3)
C012	39(3)	34(3)	40(3)	4(3)	16(3)	4(3)
C013	31(3)	48(4)	30(3)	-10(3)	7(2)	0(3)

Table S25. Bond Lengths (Å) for [**L**Cu^{III}Br][SbF_6].

		Length/Å			Length/Å
Sb01	F00C	1.885(3)	Cl0A	C012	1.767(6)
Sb01	F00E	1.871(3)	N00J	C00Q	1.357(7)
Sb01	F00F	1.882(3)	N00J	C00V	1.330(7)
Sb01	F00G	1.870(3)	N00K	C00N	1.346(6)
Sb01	F00H	1.869(3)	N00K	C00O	1.350(6)
Sb01	F00I	1.866(3)	N00L	C00M	1.351(6)
Br02	Cu03	2.4084(8)	N00L	C00P	1.344(6)
Cu03	N00J	2.056(4)	C00M	C00R	1.382(7)
Cu03	N00K	2.054(4)	C00N	C00W	1.382(7)

Table S25. Bond Lengths (Å) for [**L**Cu^{III}Br][SbF$_6$].

		Length/Å			Length/Å
Cu03	N00L	2.051(4)	C00O	C00T	1.377(7)
Cu03	C00S	2.030(5)	C00P	C00U	1.390(7)
S004	C00N	1.770(5)	C00Q	C010	1.375(7)
S004	C00S	1.772(5)	C00R	C00Z	1.391(7)
S005	C00M	1.765(5)	C00T	C00X	1.377(7)
S005	C00S	1.764(5)	C00U	C00Z	1.385(7)
S006	C00Q	1.772(5)	C00V	C00Y	1.379(8)
S006	C00S	1.774(5)	C00W	C00X	1.383(7)
Cl07	C012	1.762(6)	C00Y	C011	1.385(8)
Cl08	C013	1.771(6)	C010	C011	1.403(8)
Cl09	C013	1.762(6)			

Table S26. Bond Angles (º) for [**L**Cu^{III}Br][SbF$_6$].

			Angle/˚				Angle/˚
F00E	Sb01	F00C	90.02(15)	C00O	N00K	Cu03	126.5(3)
F00E	Sb01	F00F	178.33(15)	C00M	N00L	Cu03	116.5(3)
F00F	Sb01	F00C	88.44(15)	C00P	N00L	Cu03	126.1(3)
F00G	Sb01	F00C	90.26(15)	C00P	N00L	C00M	117.4(4)
F00G	Sb01	F00E	90.90(15)	N00L	C00M	S005	115.4(4)
F00G	Sb01	F00F	89.74(15)	N00L	C00M	C00R	123.4(5)
F00H	Sb01	F00C	88.40(16)	C00R	C00M	S005	120.9(4)
F00H	Sb01	F00E	89.28(15)	N00K	C00N	S004	115.2(4)
F00H	Sb01	F00F	90.05(15)	N00K	C00N	C00W	124.1(5)
F00H	Sb01	F00G	178.65(16)	C00W	C00N	S004	120.6(4)

Table S26. Bond Angles (º) for [$\mathbf{L}Cu^{III}Br$][SbF_6].

			Angle/°				Angle/°
F00I	Sb01	F00C	177.86(18)	N00K	C00O	C00T	123.5(5)
F00I	Sb01	F00E	91.56(16)	N00L	C00P	C00U	122.8(5)
F00I	Sb01	F00F	89.97(16)	N00J	C00Q	S006	115.1(4)
F00I	Sb01	F00G	91.16(17)	N00J	C00Q	C010	123.8(5)
F00I	Sb01	F00H	90.18(18)	C010	C00Q	S006	121.0(4)
N00J	Cu03	Br02	95.62(11)	C00M	C00R	C00Z	118.4(5)
N00K	Cu03	Br02	95.94(11)	S004	C00S	Cu03	106.5(2)
N00K	Cu03	N00J	121.34(16)	S004	C00S	S006	112.2(3)
N00L	Cu03	Br02	96.69(12)	S005	C00S	Cu03	106.1(3)
N00L	Cu03	N00J	117.59(16)	S005	C00S	S004	112.9(3)
N00L	Cu03	N00K	117.75(16)	S005	C00S	S006	113.0(3)
C00S	Cu03	Br02	179.37(15)	S006	C00S	Cu03	105.4(2)
C00S	Cu03	N00J	83.85(18)	C00O	C00T	C00X	119.2(5)
C00S	Cu03	N00K	84.04(18)	C00Z	C00U	C00P	119.0(5)
C00S	Cu03	N00L	83.87(18)	N00J	C00V	C00Y	123.4(6)
C00N	S004	C00S	98.0(2)	C00N	C00W	C00X	118.3(5)
C00S	S005	C00M	96.9(2)	C00T	C00X	C00W	118.8(5)
C00Q	S006	C00S	97.3(2)	C00V	C00Y	C011	118.6(5)
C00Q	N00J	Cu03	116.3(3)	C00U	C00Z	C00R	119.0(5)
C00V	N00J	Cu03	126.3(4)	C00Q	C010	C011	117.3(5)
C00V	N00J	C00Q	117.4(5)	C00Y	C011	C010	119.4(5)
C00N	N00K	Cu03	117.4(3)	Cl07	C012	Cl0A	110.6(3)
C00N	N00K	C00O	116.1(4)	Cl09	C013	Cl08	110.6(3)

Table S27. Torsion Angles for [**L**Cu^{III}Br][SbF$_6$].

				Angle/°					Angle/°
Cu03	N00J	C00Q	S006	-0.6(5)	C00N	S004	C00S	S005	158.6(3)
Cu03	N00J	C00Q	C010	176.5(4)	C00N	S004	C00S	S006	-72.3(3)
Cu03	N00J	C00V	C00Y	-176.0(4)	C00N	N00K	C00O	C00T	1.9(7)
Cu03	N00K	C00N	S004	0.1(5)	C00N	C00W	C00X	C00T	1.6(8)
Cu03	N00K	C00N	C00W	176.7(4)	C00O	N00K	C00N	S004	-178.1(3)
Cu03	N00K	C00O	C00T	-176.1(4)	C00O	N00K	C00N	C00W	-1.5(7)
Cu03	N00L	C00M	S005	3.1(5)	C00O	C00T	C00X	C00W	-1.3(8)
Cu03	N00L	C00M	C00R	177.9(4)	C00P	N00L	C00M	S005	-177.0(4)
Cu03	N00L	C00P	C00U	-177.1(4)	C00P	N00L	C00M	C00R	-2.2(7)
S004	C00N	C00W	C00X	176.2(4)	C00P	C00U	C00Z	C00R	-0.2(8)
S005	C00M	C00R	C00Z	174.8(4)	C00Q	S006	C00S	Cu03	45.5(3)
S006	C00Q	C010	C011	178.0(4)	C00Q	S006	C00S	S004	161.1(3)
N00J	C00Q	C010	C011	1.1(8)	C00Q	S006	C00S	S005	-69.9(3)
N00J	C00V	C00Y	C011	-2.0(8)	C00Q	N00J	C00V	C00Y	3.4(7)
N00K	C00N	C00W	C00X	-0.2(8)	C00Q	C010	C011	C00Y	0.5(8)
N00K	C00O	C00T	C00X	-0.5(8)	C00S	S004	C00N	N00K	-27.6(4)
N00L	C00M	C00R	C00Z	0.3(8)	C00S	S004	C00N	C00W	155.7(4)
N00L	C00P	C00U	C00Z	-1.8(8)	C00S	S005	C00M	N00L	-31.2(4)
C00M	S005	C00S	Cu03	45.3(3)	C00S	S005	C00M	C00R	153.8(4)
C00M	S005	C00S	S004	-71.0(3)	C00S	S006	C00Q	N00J	-29.1(4)
C00M	S005	C00S	S006	160.3(3)	C00S	S006	C00Q	C010	153.6(4)
C00M	N00L	C00P	C00U	3.0(7)	C00V	N00J	C00Q	S006	179.9(4)
C00M	C00R	C00Z	C00U	1.0(7)	C00V	N00J	C00Q	C010	-3.0(7)
C00N	S004	C00S	Cu03	42.5(3)	C00V	C00Y	C011	C010	-0.1(8)

Table S28. Hydrogen Atom Coordinates (Å×10^4) and Isotropic Displacement Parameters (Å^2×10^3) for [**L**Cu^{III}Br][SbF_6].

	x	*y*	*z*	U(eq)
H00O	7044.28	7488.25	10519.89	27
H00P	7113.1	5995.66	7940.01	32
H00R	10162.77	6118.86	5905.58	30
H00T	5897.75	8304.1	10308.12	33
H00U	7028.67	5423.09	6382.07	34
H00V	10153.65	6011.39	11118.7	37
H00W	7461.66	8740.7	7721.52	33
H00X	6065.46	8938.01	8873.94	34
H00Y	11868.7	5970.14	12459.97	43
H00Z	8567.64	5480.05	5340.08	33
H010	12916.29	7472.46	11287.66	38
H011	13286.15	6703.71	12559.43	43
H01A	11805.64	5327.91	7609.54	44
H01B	12129.43	4762.64	8299.44	44
H01C	4382.85	6513.43	7932.04	43
H01D	5555.07	6644.01	8808.72	43

Table S29. Solvent masks information for [**L**Cu^{III}Br][SbF_6].

Number	X	Y	Z	Volume	Electron count	Content
1	0.500	0.000	0.500	238.0	81.2	2 DCM
2	0.500	0.500	0.000	238.0	81.2	2 DCM

References

(1) Queener, J.; Asor, A.; Ball, M.; Fang, J.; Tang, J.; Zhang, S. Distinct Hydrogen Atom Transfer and Radical Capture Reactivity of Copper (III) OH/F Complexes Enables Site-Selective C (Sp^3)-H ^{18}F-Fluorination. *Chem. Sci.* **2026**, *17*, 299–306.

(2) Kinoshita, I.; Hashimoto, H.; Nishioka, T.; Miyamoto, R.; Kuwamura, N.; Yoshida, Y. Mimicking the Photosynthetic System with Strong Hydrogen Bonds to Promote Proton Electron Concerted Reactions. *Photosynth Res* **2008**, *95*, 363–371.

(3) Stevens, M. P.; Spray, E.; Vitorica-yrezabal, I. J.; Singh, K.; Timmermann, V. M.; Sotorrios, L.; Ortu, F. Structural Investigation of Magnesium Complexes Supported by a Thiopyridyl Scorpionate Ligand. *molecules* **2022**, *27*, 4564.

(4) Miyamoto, R.; Santo, R.; Matsushita, T.; Nishioka, T.; Ichimura, A.; Teki, Y.; Kinoshita, I. A Complete Series of Copper(II) Halide Complexes (X = F, Cl, Br, I) with a Novel Cu(II)-C(Sp^3) Bond. *Dalt. Trans.* **2005**, 3179–3186.

(5) Lielpetere, A.; Jirgensons, A. Carbenium Ion Formation of Electrochemically Generated Oxonium Ions†. *Org. Biomol. Chem.* **2018**, *16*, 5094.

(6) Bietti, M.; Gente, G.; Salamone, M. Structural Effects on the β-Scission Reaction of Tertiary Arylcarbinyloxyl Radicals . The Role of α-Cyclopropyl and α-Cyclobutyl Groups. *J. Org. Chem.* **2005**, *70*, 6820–6826.

(7) Bloom, S.; McCann, M.; Lectka, T. Photocatalyzed Benzylic Fluorination: Shedding "Light" on the Involvement of Electron Transfer. *Org. Lett.* **2014**, *16*, 6338–6341.

(8) Lu, Z.; Zeng, X.; Hammond, G. B.; Xu, B. Widely Applicable Hydro Fl Uorination of Alkenes via Bifunctional Activation of Hydrogen Fluoride. *J. Am. Chem. Soc.* **2017**, *139*, 18202–18205.

(9) Ortalli, S.; Ford, J.; Trabanco, A. A.; Tredwell, M.; Gouverneur, V. Photoredox Nucleophilic (Radio)Fluorination of Alkoxyamines. *J. Am. Chem. Soc.* **2024**, *146*, 11599–11604.

(10) Hintz, H.; Bower, J.; Tang, J.; LaLama, M.; Sevov, C.; Zhang, S. Copper-Catalyzed Electrochemical C–H Fluorination. *Chem Catal.* **2023**, *3*, 100491.

(11) Moon, H. W.; Lavagnino, M. N.; Lim, S.; Palkowitz, M. D.; Mandler, M. D.; Beutner, G. L.; Drance, M. J.; Lipshultz, J. M.; Scola, P. M.; Radosevich, A. T. Deoxyfluorination of 1°, 2°, and 3° Alcohols by Nonbasic O–H Activation and Lewis Acid-Catalyzed Fluoride Shuttling. *J. Am. Chem. Soc.* **2023**, *145*, 22735–22744.

(12) Kirihara, M.; Takuwa, T.; Kambayashi, T.; Momose, T.; Takeuchi, Y. Novel Fluorination of Small-Ring Tertiary Cycloalkanols : Reaction of Diethylaminosulfur Trifluoride with Tertiary Cyclobutanols. *J. Chem. Res.* **1998**, 652–653.

(13) Zhang, Y.; Zhu, S.; Zuo, Y.; Liu, H.; Jin, R.; Wang, X. Visible Light-Induced Photocatalytic Deoxyfluorination of Benzyl Alcohol Using SF_6 as a Fluorinating Reagent. *Green Chem.* **2024**, *26*, 10324.

(14) Kanemoto, S.; Shimizu, M.; Yoshioka, H. Ring-Opening Fluorination and Ring-Expansion Fluorination of Cyclopropanemethanols with Amine/Metal Fluoride/Poly(Hydrogen Fluoride)-Pyridine Complex. *Bull. Chem. Soc. Jpn.* **1989**, *62*, 2024–2031.

(15) Sunagatullina, A. S.; Hess, A.; Kremsmair, A.; Li, Y.; Chen, Y. H.; Knochel, P. Transition-Metal Free Electrophilic Aminations of Polyfunctional O-2,4,6-Trimethylbenzoyl Hydroxylamines with

Zinc and Magnesium Organometallics. *Angew. Chemie - Int. Ed.* **2023**, *62*, 1–5.

(16) Lang, S.; Kennedy, A. R.; Murphy, J. A.; Payne, A. H. Amination of Arenes through Electron-Deficient Reaction Cascades of Aryl Epoxyazides. *Org. Lett.* **2003**, *5*, 3655–3658.

(17) Musacchio, A. J.; Nguyen, L. Q.; Beard, G. H.; Knowles, R. R. Photoredox Method for Direct C − N Bond Formation. *J. Am. Chem. Soc.* **2014**, 15–18.

(18) Xing, X.; He, Y. T.; Song, J. G.; Lan, P.; White, L. V.; Tan, S.; Pham, L. N.; Coote, M. L.; Wu, X.; Banwell, M. G. Synthesis and Ring-Closing Reactions of Aminocyclohexane Derivatives Bearing Unsaturated Side Chains at C2: Stereocontrolled Approaches to Cis- and Trans-Fused Perhydro-Indoles and -Quinolines. *European J. Org. Chem.* **2024**, *27*, 1–8.

(19) Kinoshita, I.; James Wright, L.; Kubo, S.; Kimura, K.; Sakata, A.; Yano, T.; Miyamoto, R.; Nishioka, T.; Isobe, K. Design and Synthesis of Copper Complexes of Novel Ligands Based on the Pyridine Thiolate Group. *Dalt. Trans.* **2003**, No. 10, 1993–2003.

(20) Neese, F. Software Update: The ORCA Program System—Version 5.0. *Wiley Interdiscip. Rev. Comput. Mol. Sci.* **2022**, *12*, 1–15.

(21) Neese, F.; Wennmohs, F.; Becker, U.; Riplinger, C. The ORCA Quantum Chemistry Program Package. *J. Chem. Phys.* **2020**, *152*, 224108.

(22) Santo, R.; Miyamoto, R.; Tanaka, R.; Nishioka, T.; Sato, K.; Toyota, K.; Obata, M.; Yano, S.; Kinoshita, I.; Ichimura, A.; Takui, T. Diamagnetic–Paramagnetic Conversion of Tris(2-Pyridylthio)Methylcopper(III) through a Structural Change from Trigonal Bipyramidal to Octahedral. *Angew. Chemie Int. Ed.* **2006**, *45*, 7611–7614.

(23) Bourhis, L. J.; Dolomanov, O. V.; Gildea, R. J.; Howard, J. A. K.; Puschmann, H. The Anatomy of a Comprehensive Constrained, Restrained Refinement Program for the Modern Computing Environment - Olex2 Dissected. *Acta Crystallogr. Sect. A Found. Crystallogr.* **2015**, *71*, 59–75.

(24) Dolomanov, O. V.; Bourhis, L. J.; Gildea, R. J.; Howard, J. A. K.; Puschmann, H. OLEX2: A Complete Structure Solution, Refinement and Analysis Program. *J. Appl. Crystallogr.* **2009**, *42*, 339–341.

(25) Kratzert, D.; Holstein, J. J.; Krossing, I. DSR: Enhanced Modelling and Refinement of Disordered Structures with SHELXL. *J. Appl. Crystallogr.* **2015**, *48*, 933–938.

(26) Kratzert, D.; Krossing, I. Recent Improvements in DSR. *J. Appl. Crystallogr.* **2018**, *51*, 928–934.

(27) Sheldrick, G. M. A Short History of SHELX. *Acta Crystallogr. Sect. A Found. Crystallogr.* **2008**, *64*, 112–122.

(28) Sheldrick, G. M. SHELXT - Integrated Space-Group and Crystal-Structure Determination. *Acta Crystallogr. Sect. A Found. Crystallogr.* **2015**, *71*, 3–8.
